# Prognostic factors for the development and progression of proliferative diabetic retinopathy in people with diabetic retinopathy

**DOI:** 10.1002/14651858.CD013775.pub2

**Published:** 2023-02-22

**Authors:** Jennifer Perais, Ridhi Agarwal, Jennifer R Evans, Emma Loveman, Jill L Colquitt, David Owens, Ruth E Hogg, John G Lawrenson, Yemisi Takwoingi, Noemi Lois

**Affiliations:** The Wellcome-Wolfson Institute for Experimental MedicineQueen's University BelfastBelfastUK; Institute of Applied Health ResearchUniversity of BirminghamBirminghamUK; Cochrane Eyes and VisionQueen's University BelfastBelfastUK; Effective Evidence LLPWaterloovilleUK; Cardiff UniversityCardiffUK; Centre for Public HealthQueen's University BelfastBelfastUK; Centre for Applied Vision Research, School of Health SciencesCity University of LondonLondonUK; Wellcome-Wolfson Institute for Experimental MedicineQueen's University BelfastBelfastUK

**Keywords:** Adult, Female, Humans, Male, Diabetes Mellitus, Type 1, Diabetes Mellitus, Type 1/complications, Diabetes Mellitus, Type 2, Diabetes Mellitus, Type 2/complications, Diabetic Retinopathy, Diabetic Retinopathy/complications, Glycated Hemoglobin, Prognosis, Prospective Studies, Retinal Hemorrhage, Retrospective Studies, Triglycerides, Vitreous Hemorrhage, Vitreous Hemorrhage/complications

## Abstract

**Background:**

Diabetic retinopathy (DR) is characterised by neurovascular degeneration as a result of chronic hyperglycaemia. Proliferative diabetic retinopathy (PDR) is the most serious complication of DR and can lead to total (central and peripheral) visual loss. PDR is characterised by the presence of abnormal new blood vessels, so‐called “new vessels,” at the optic disc (NVD) or elsewhere in the retina (NVE). PDR can progress to high‐risk characteristics (HRC) PDR (HRC‐PDR), which is defined by the presence of NVD more than one‐fourth to one‐third disc area in size plus vitreous haemorrhage or pre‐retinal haemorrhage, or vitreous haemorrhage or pre‐retinal haemorrhage obscuring more than one disc area. In severe cases, fibrovascular membranes grow over the retinal surface and tractional retinal detachment with sight loss can occur, despite treatment. Although most, if not all, individuals with diabetes will develop DR if they live long enough, only some progress to the sight‐threatening PDR stage.

**Objectives:**

To determine risk factors for the development of PDR and HRC‐PDR in people with diabetes and DR.

**Search methods:**

We searched the Cochrane Central Register of Controlled Trials (CENTRAL; which contains the Cochrane Eyes and Vision Trials Register; 2022, Issue 5), Ovid MEDLINE, and Ovid Embase. The date of the search was 27 May 2022. Additionally, the search was supplemented by screening reference lists of eligible articles. There were no restrictions to language or year of publication.

**Selection criteria:**

We included prospective or retrospective cohort studies and case‐control longitudinal studies evaluating prognostic factors for the development and progression of PDR, in people who have not had previous treatment for DR. The target population consisted of adults (≥18 years of age) of any gender, sexual orientation, ethnicity, socioeconomic status, and geographical location, with non‐proliferative diabetic retinopathy (NPDR) or PDR with less than HRC‐PDR, diagnosed as per standard clinical practice. Two review authors independently screened titles and abstracts, and full‐text articles, to determine eligibility; discrepancies were resolved through discussion. We considered prognostic factors measured at baseline and any other time points during the study and in any clinical setting. Outcomes were evaluated at three and eight years (± two years) or lifelong.

**Data collection and analysis:**

Two review authors independently extracted data from included studies using a data extraction form that we developed and piloted prior to the data collection stage. We resolved any discrepancies through discussion. We used the Quality in Prognosis Studies (QUIPS) tool to assess risk of bias. We conducted meta‐analyses in clinically relevant groups using a random‐effects approach. We reported hazard ratios (HR), odds ratios (OR), and risk ratios (RR) separately for each available prognostic factor and outcome, stratified by different time points. Where possible, we meta‐analysed adjusted prognostic factors. We evaluated the certainty of the evidence with an adapted version of the GRADE framework.

**Main results:**

We screened 6391 records. From these, we identified 59 studies (87 articles) as eligible for inclusion. Thirty‐five were prospective cohort studies, 22 were retrospective studies, 18 of which were cohort and six were based on data from electronic registers, and two were retrospective case‐control studies. Twenty‐three studies evaluated participants with type 1 diabetes (T1D), 19 with type 2 diabetes (T2D), and 17 included mixed populations (T1D and T2D). Studies on T1D included between 39 and 3250 participants at baseline, followed up for one to 45 years. Studies on T2D included between 100 and 71,817 participants at baseline, followed up for one to 20 years. The studies on mixed populations of T1D and T2D ranged from 76 to 32,553 participants at baseline, followed up for four to 25 years.

We found evidence indicating that higher glycated haemoglobin (haemoglobin A1c (HbA1c)) levels (adjusted OR ranged from 1.11 (95% confidence interval (CI) 0.93 to 1.32) to 2.10 (95% CI 1.64 to 2.69) and more advanced stages of retinopathy (adjusted OR ranged from 1.38 (95% CI 1.29 to 1.48) to 12.40 (95% CI 5.31 to 28.98) are independent risk factors for the development of PDR in people with T1D and T2D. We rated the evidence for these factors as of moderate certainty because of moderate to high risk of bias in the studies.

There was also some evidence suggesting several markers for renal disease (for example, nephropathy (adjusted OR ranged from 1.58 (95% CI not reported) to 2.68 (2.09 to 3.42), and creatinine (adjusted meta‐analysis HR 1.61 (95% CI 0.77 to 3.36)), and, in people with T1D, age at diagnosis of diabetes (< 12 years of age) (standardised regression estimate 1.62, 95% CI 1.06 to 2.48), increased triglyceride levels (adjusted RR 1.55, 95% CI 1.06 to 1.95), and larger retinal venular diameters (RR 4.28, 95% CI 1.50 to 12.19) may increase the risk of progression to PDR. The certainty of evidence for these factors, however, was low to very low, due to risk of bias in the included studies, inconsistency (lack of studies preventing the grading of consistency or variable outcomes), and imprecision (wide CIs). There was no substantial and consistent evidence to support duration of diabetes, systolic or diastolic blood pressure, total cholesterol, low‐ (LDL) and high‐ (HDL) density lipoproteins, gender, ethnicity, body mass index (BMI), socioeconomic status, or tobacco and alcohol consumption as being associated with incidence of PDR. There was insufficient evidence to evaluate prognostic factors associated with progression of PDR to HRC‐PDR.

**Authors' conclusions:**

Increased HbA1c is likely to be associated with progression to PDR; therefore, maintaining adequate glucose control throughout life, irrespective of stage of DR severity, may help to prevent progression to PDR and risk of its sight‐threatening complications. Renal impairment in people with T1D or T2D, as well as younger age at diagnosis of diabetes mellitus (DM), increased triglyceride levels, and increased retinal venular diameters in people with T1D may also be associated with increased risk of progression to PDR. Given that more advanced DR severity is associated with higher risk of progression to PDR, the earlier the disease is identified, and the above systemic risk factors are controlled, the greater the chance of reducing the risk of PDR and saving sight.

## Plain language summary

**Risk factors for the development and progression of proliferative diabetic retinopathy (a diabetes complication affecting eyes)**

**Review question**

We wanted to find out which factors may increase or reduce the chance that people with diabetes develop proliferative diabetic retinopathy and high‐risk proliferative diabetic retinopathy, both sight‐threatening complications of diabetes.

**Background**

In diabetes, over time, raised blood sugar levels damage fine blood vessels in the retina, the layer at the back of the eye that gives people sight. This is called ‘diabetic retinopathy’. In some people with diabetes and diabetic retinopathy, abnormal and fragile blood vessels grow in the retina: so‐called 'new vessels'. When new vessels are present, we say there is 'proliferative diabetic retinopathy', also called 'PDR'. These new vessels are weak and can bleed inside the eye, causing what is known as a 'vitreous haemorrhage'. The blood inside the eye takes away the vision, although, if it clears on its own (which sometimes happens) or with surgery, vision most often recovers. Scar tissue can also grow over the new vessels. Scarring can pull on the retina and cause what is known as a tractional retinal detachment, the most severe sight‐threatening complication of diabetic retinopathy. Tractional retinal detachment can cause total blindness if not treated with surgery promptly.

While most people with diabetes develop diabetic retinopathy, only a few progress to these severe complications. It is unclear why this is the case. Sight loss is usually preventable if treatment is done early. Therefore, it is essential to know who is at risk of progressing to PDR, so that these people can be followed closely and treated in a timely way. We did this review to find out the risk factors which may determine why some people develop PDR.

**Study characteristics**

We included studies in which people with diabetes, who had never been treated for diabetic retinopathy, were followed up over time to determine who developed PDR and who progressed to severe stages of PDR (called 'high‐risk characteristics PDR' (HRC‐PDR)). To be included in our review, these studies had to investigate risk factors for PDR and HRC‐PDR: for example, blood sugar, blood pressure, cholesterol, and kidney disease, amongst others. We included studies looking at adults (18 years of age and older) of any gender, ethnicity, sexual orientation, socioeconomic status, and nation, written in any language, in this review.

**Key results**

Of the 6391 articles we found, 59 studies (87 articles) were eligible, and we included them in our review. We found that higher blood sugar (which means poorer diabetes control) and more advanced diabetic retinopathy (more changes from diabetes in the retina) put people at higher risk of having PDR. People with kidney disease seemed also to be at higher risk of progressing to PDR. It is also possible that people with type 1 diabetes who were diagnosed at a young age, and those with higher triglyceride levels (triglycerides are a type of fat in the blood, like cholesterol) or who have retinal veins with larger diameters, are more at risk of developing PDR. Other risk factors studied ‐ for example, duration of diabetes, blood pressure, and cholesterol ‐ did not seem to be risk factors for PDR. There was not enough information from the included studies for us to analyse risk factors for HRC‐PDR.

**Authors' conclusions**

People living with type 1 or type 2 diabetes who have poor blood sugar control are likely to be at increased risk of developing PDR. Evidence suggests that better blood sugar control, even in people who already have the earlier stages of diabetic retinopathy, may help to prevent it from progressing to PDR. Those with kidney disease may also be at increased risk of progressing to PDR. Additionally, people with type 1 diabetes, who were diagnosed at a younger age, or who have higher triglyceride levels or larger retinal veins, may be more susceptible to developing PDR.

**How up to date is this evidence?**

The evidence is up to date to 27 May 2022.

## Summary of findings

**Summary of findings 1:** Prognostic factors for the development and progression of PDR in people with diabetic retinopathy: demographic factors

Population: people with diabetes Outcome: progression to PDR			
**Prognostic factors**	**Study results: effect estimates (95% confidence interval (CI))**	**Certainty of evidence**	**Plain text summary**
**Gender** (males versus females) (Refer to Table 2 for adjustment factors)	**T1D and T2D** (follow‐up 4 to 6 years)** ** **Adjusted HR** ranged from 0.92 (0.71 to 1.19) to 1.08 (0.94 to 1.22) Data from 93,246 participants in 4 studies **Adjusted RR** 1.5 (0.70 to 3.40) Data from 953 participants in 1 study	Moderate^a^	Gender is not likely to increase risk of developing PDR
**Ethnicity** (Refer to Table 3 for adjustment factors)	**T1D** (follow‐up 7 years) **Adjusted OR** 0.73 (0.30 to 1.78) (African American vs. White) Data from 312 participants in 1 study **T2D** (follow‐up 5 to 10 years) **Adjusted HR** 0.94 (0.89 to 1.00) (Non‐White vs. White ) **Adjusted OR** 4.4 (2.0 to 9.7) (Ashkenazi Jews vs. Non‐Ashkenazi Jews) Data from 32,883 participants in 2 studies **Mixed T1D and T2D** (follow‐up 5 years) **Adjusted HR** 1.29 (0.92 to 1.82; P > 0.05) (Black); 1.12 (0.76 to 1.65; P > 0.05) (Latino); 1.35 (0.73 to 2.49; P > 0.05) (Asian) Data from 4617 participants in 1 study	Very low^a,b,c ^	The evidence is very uncertain about the effect of ethnicity on risk of developing PDR
**Age at diagnosis of DM** (Refer to Table 4 for adjustment factors)	**T1D** (follow‐up 7 years) **Adjusted standardised regression estimate** 1.62 (1.06 to 2.48; P = 0.038) (< 12 years) Data from 2013 participants in 1 study **T2D** (follow‐up 5 years) Adjusted OR 0.46 (0.29 to 0.74) (18 to 34 years vs. 45 to 54 years); 1.25 (1.05 to 1.48) (55 to 64 vs. 45 to 54 years); 1.62 (1.28 to 2.03) (65 to 74 vs. 45 to 54 years); 1.30 (1.00 to 1.68) (≥ 75 vs. 45 to 54 years)	Low^a,b^	Evidence from one study in T1D, suggesting age of diagnosis < 12 years may be associated with progression to PDR in T1D Evidence from one study in T2D, suggesting age of diagnosis between 18 to 34 vs. 45 to 54 years may decrease risk of progression to PDR, and age of diagnosis between 55 to 74 vs. 45 to 54 years may increase risk of progression to PDR
**Duration of DM** (Refer to Table 5 for adjustment factors)	**T1Dand T2D** (follow‐up 2 to 25 years) **Adjusted OR** ranged from 0.69 (0.35 to 1.36) to 1.20 (1.10 to 1.30). Data from 5591 participants in 4 studies **Adjusted RR** ranged from 1.03 (0.94 to 1.12) to 1.95 (1.58 to 2.39). Data from 4206 participants in 3 studies **Adjusted HR** 1.21 (1.10 to 1.79). Data from 452 participants in 1 study	Very low^a,b,c ^	Evidence is very uncertain about the effect of duration of DM on progression to PDR (duration of DM was not independently associated with development of PDR when correcting for other important risk factors, including HbA1C and DR severity at baseline)
**Type of DM**	**T1D and T2D** (follow‐up 5 to 8 years) **Adjusted RR** 0.62 (0.50 to 0.76) (T1D) 0.91 (0.72 to 1.13) (insulin‐treated T2D) **Adjusted HR** 0.86 (95% CI not reported; P value not statistically significant) (T1D)	Very low^a,b,c ^	Evidence is very uncertain about the effect of type of DM on progression to PDR, but T1D may have a protective effect
**Socioeconomic status** (Refer to Table 6 for adjustment factors)	**T1D** (follow‐up 4 years) **Adjusted OR** 0.78 (0.52 to 1.18) (males, per 10‐point increase); 0.79 (0.46 to 1.37) (females, per 10‐point increase) Data from 996 participants in 1 study **T2D** (follow‐up 4 years) **Adjusted OR** 0.84 (0.58 to 1.23) (males, per 10‐point increase); 0.88 (0.55 to 1.41) (females, per 10‐point increase). Data from 1370 participants in 1 study	Very low^a,b,c ^	Evidence is very uncertain about the effect of socioeconomic status on progression to PDR
**Education level** (Refer to Table 7 for adjustment factors)	**T1D** (follow‐up 4 years) **Adjusted OR** 0.59 (0.20 to 1.78) (males, per 10‐point increase); 0.26 (0.07 to 0.99) (females, per 10‐point increase) Data from 996 participants in 1 study **T2D** (follow‐up 4 years) **Adjusted OR** 0.50 (0.21 to 1.16) (males, per 10‐point increase); 0.90 (0.33 to 2.48) (females, per 10‐point increase) Data from 1370 participants in 1 study	Very low^a,b,c ^	Evidence is very uncertain about the effect of education level on progression to PDR

**CI:** confidence interval; **DM:** diabetes mellitus; **HR:** hazard ratio; **OR:** odds ratio; **PDR:** proliferative diabetic retinopathy; **RR:** risk ratio; **T1D:** type 1 diabetes; **T2D:** type 2 diabetes ^a^Downgraded by one level for risk of bias: more than 80% of studies at high or unclear risk of bias ^b^Downgraded by one level for inconsistency: significant differences in effect estimates reported by studies ^c^Downgraded by one level for imprecision: wide 95% CIs

**1:** Gender ‐ Studies undertaking multivariable regression analyses to determine the effect of gender on progression to PDR

**Study**	**Study type**	**Time years **	**N at baseline**	**Adjustment factors**	**Effect estimate**			**P value**	**Comments**
					Type	Value	95% CI		
**Type 1 diabetes**									
No multivariable regression analyses									
**Type 2 diabetes**									
Nelson 1989	Prospective cohort	4	953	DM duration, age	RR	1.5	0.7 to 3.4		Male vs female
Gange 2021	Retrospective cohort (electronic database)	5	718	Maximum HbA1c, gender, smoking, comorbidities, obesity, insulin use, education, hypertension, dyslipidaemia, diabetic ketoacidosis	Gender reported narratively in text as being non‐significant.				
Lee 2021	Retrospective cohort	6	2623	HbA1c, DR severity at baseline, age, BMI					Male vs female
**Type 1 and type 2 diabetes**									
Lee 2017	Retrospective cohort (electronic database)	5	32,553	DR severity at baseline, age, ethnicity, features of DR	HR	0.92^a^	0.71 to 1.19	0.53	
Harris 2013	Retrospective cohort (electronic database)	5	4617	HbA1c, age, ethnicity, comorbidities, medications	HR	1.08	0.94 to 1.22		
Jeng 2016	Retrospective cohort (electronic database)	5	53,453	Age, comorbidities, medications	HR	0.99	0.85 to 1.15		Female vs male

**BMI:** body mass index; **CI:** confidence interval; **DM:** diabetes mellitus; **DR:** diabetic retinopathy; **HbA1c:** glycated haemoglobin/haemoglobin A1c; **HR:** hazard ratio; **NPDR:** non‐proliferative diabetic retinopathy; **OR:** odds ratio; **PDR:** proliferative diabetic retinopathy; **RR:** risk ratio; **vs:** versus ^a^ Reference gender not reported: authors contacted but unable to confirm

**2:** Ethnicity ‐ Studies undertaking multivariable regression analyses to determine the effect of ethnicity on progression to PDR

**Study**	**Study type**	**Time years **	**N at baseline**	**Adjustment factors**	**Effect estimate**			**P value**	**Comments**
					Type	Value	95% CI		
**Type 1 diabetes**									
Arfken 1998	Retrospective cohort	7	312	HbA1c, DR severity at baseline, follow‐up period	OR	0.73^a^	0.30 to 1.78	0.49	
**Type 2 diabetes**									
Lee 2017	Retrospective cohort (electronic database)	5	32553	DR severity at baseline, age, sex, VA, DR features	HR	0.94^b^	0.89 to 1.00	0.65	
Kalter‐Leibovici 1991	Prospective cohort	10	330	HbA1c, DM duration, socioeconomic status	OR	4.4^c^	2.00 to 9.70		
**Type 1 and type 2 diabetes**									
Harris 2013	Prospective cohort (electronic database)	5	4617	Age, sex, comorbidities, medications	HR	1.00^d^ 1.29^e^ 1.12^f^ 1.35^g^	0.92 to 1.82 0.76 to 1.65 0.73 to 2.49	> 0.05 > 0.05 > 0.05	

**BMI:** body mass index; **CI:** confidence interval; **DM:** diabetes mellitus; **DR:** diabetic retinopathy; **HbA1c:** glycated haemoglobin/haemoglobin A1c; **HR:** hazard ratio; **NPDR:** non‐proliferative diabetic retinopathy; **OR:** odds ratio; **PDR:** proliferative diabetic retinopathy; **RR:** risk ratio; **VA:** visual acuity ^a^African American versus Caucasian (understood to be White) ^b^Non‐Caucasian versus Caucasian (understood to be Non‐White versus White) ^c^Ashkenazi Jews versus non‐Ashkenazi Jews ^d^White ^e^Black ^f^Latino ^g^Asian

**3:** Age at diagnosis of diabetes ‐ Studies undertaking multivariable regression analyses to determine the effect of age at diagnosis of diabetes on progression to PDR

**Study**	**Study type**	**Time years **	**N at baseline**	**Adjustment factors**	**Effect estimate**			**P value**
					Type	Value	95% CI	
**Type 1 diabetes**								
Porta 2001	Prospective cohort	7	2013	HbA1c, DM duration, severity at baseline, DBP > 83 mmg DR, waist‐to‐hip ratio	Standardised regression estimate	1.62	1.06 to 2.48	0.038^a^
**Type 2 diabetes**								
Gange 2021	Prospective cohort (electronic database)	5	718	Maximum HbA1c, gender, smoking, comorbidities, obesity, insulin use, education, hypertension, dyslipidaemia, diabetic ketoacidosis	OR	0.46^b^ 1.25^c^ 1.62^d^ 1.30^e^	0.29 to 0.74 1.05 to 1.48 1.28 to 2.03 1.00 to 1.68	0.001 0.012 < 0.001 0.048

**BMI:** body mass index; **CI:** confidence interval; **DBP:** diastolic blood pressure; **DM:** diabetes mellitus; **DR:** diabetic retinopathy; **HbA1c:** glycated haemoglobin/haemoglobin A1c; **HR:** hazard ratio; **NPDR:** non‐proliferative diabetic retinopathy; **OR:** odds ratio; **PDR:** proliferative diabetic retinopathy; **RR:** risk ratio; **vs:** versus ^a^Effect did not remain significant when albumin excretion rate included as a covariate ^b^18 to 34 years vs 45 to 54 years ^c^55 to 64 years vs 45 to 54 years  ^d^65 to 74 years vs 45 to 54 years ^e^≥75 years vs 45 to 54 years

**4:** Duration of diabetes ‐ Studies undertaking multivariable regression analyses to determine the effect of duration of diabetes on progression to PDR

**Study**	**Study type**	**Time years **	**N at baseline**	**Adjustment factors**	**Effect estimate**			**P value**	**Comments**
					Type	Value	95% CI		Per increase in one year
**Type 1 diabetes**									
Lloyd 1995	Prospective cohort	2	496	HbA1c, DR severity at baseline, follow‐up period	RR	1.03	0.94 to 1.12		
Janghorbani 2000	Retrospective cohort	5	1349	HbA1c, SBP	RR	1.00^a^ 0.78^b^ 1.95^c^ 3.05^d^	0.43 to 1.41 1.23 to 3.09 2.09 to 4.45	Nonsignificant < 0.01 < 0.001	
Porta 2001	Prospective cohort	7	2013	HbA1c, age, DM diagnosis < 12 years, DBP, albumin excretion rate, waist‐to‐height ratio	Regression estimate	1.71 1.12^e^	1.42 to 2.06 0.89 to 1.42	0.0001 0.3	Increasing duration of diabetes
Kalter‐Leibovici 1991	Prospective cohort	10	330	HbA1c, age, sex, race, socioeconomic status	OR	1.20	1.1 to 1.3		Increasing duration of diabetes
Grauslund 2009	Prospective cohort	25	573	HbA1c, DR severity at baseline, age, sex, SBP, DBP, proteinuria, BMI, smoking status, maculopathy	OR	0.69	0.35 to 1.36		Per 10 years
**Type 2 diabetes**									
Gui 2013	Retrospective cohort	2	205	Age, hypertension, smoking status, C‐peptide	OR	1.18	1.13 to 1.25	< 0.05	Mean
Janghorbani 2000	Retrospective cohort	5	2133	Unclear	RR	1.77^f^ 1.37^g^	1.15 to 2.72 0.83 to 2.26	< 0.05 > 0.05	Insulin Non‐insulin
Kim 1998	Prospective cohort	5	228	HbA1c, age, albumin excretion rate, change in BMI	RR	1.15	0.99 to 1.32		
Kim 2014	Retrospective cohort	5	452	HbA1c	HR	1.21	1.10 to 1.79	0.17	Per unit increase
Lee 1992	Prospective cohort	12	354	Fasting plasma glucose, age, SBP, cholesterol, DM treatment	Regression estimate	0.09	Standard error: 0.03	< 0.001	Per unit increase
**Type 1 and type 2 diabetes**									
Janghorbani 2000	Retrospective cohort	5	3482	HbA1c, SBP, proteinuria, type of DM	RR	1.42^c^ 1.95^d^	1.10‐1.83 1.58‐2.39	< 0.01 < 0.001	8 to 11 years ≥ 12 years
Keen 2001	Prospective cohort	8	4483	Age, sex, SBP, DBP, cholesterol, BMI, smoking status, insulin, type of DM, comorbidities	OR	1.16		< 0.01	Per 5 years

**BMI:** body mass index; **CI:** confidence interval; **DBP:** diastolic blood pressure; **DM:** diabetes mellitus; **DR:** diabetic retinopathy; **HbA1c:** glycated haemoglobin/haemoglobin A1c; **HR:** hazard ratio; **NPDR:** non‐proliferative diabetic retinopathy; **OR:** odds ratio; **PDR:** proliferative diabetic retinopathy; **RR:** risk ratio; **SBP:** systolic blood pressure; **vs:** versus ^a^< 4 years ^b^4 to 7 years vs. < 4 years ^c^8 to 11 years vs. < 4 years ^d^≥ 12 years vs. < 4 years ^e^Model also included DR severity at baseline ^f^≥ 12 years vs. < 4 years, taking insulin ^g^≥ 12 years vs. < 4 years, not taking insulin

**5:** Socioeconomic status ‐ Studies undertaking multivariable regression analyses to determine the effect of socioeconomic status on progression to PDR

**Study**	**Study type**	**Time years **	**N at baseline**	**Adjustment factors**	**Effect estimate**			**P value**	**Comments**
					Type	Value	95% CI		
**Type 1 diabetes**									
WESDR Klein 1994	Prospective cohort	4	996	HbA1c, DR severity at baseline	OR	0.78^a^ 0.79^b^	0.52 to 1.18 0.46 to 1.37		Per 10‐point increase
**Type 2 diabetes**									
WESDR Klein 1994	Prospective cohort	4	1370	HbA1c, DR severity at baseline	OR	0.84^a^ 0.88^b^	0.58 to 1.23 0.55 to 1.41		Per 10‐point increase

**BMI:** body mass index; **CI:** confidence interval; **DBP:** diastolic blood pressure; **DM:** diabetes mellitus; **DR:** diabetic retinopathy; **HbA1c:** glycated haemoglobin/haemoglobin A1c; **HR:** hazard ratio; **NPDR:** non‐proliferative diabetic retinopathy; **OR:** odds ratio; **PDR:** proliferative diabetic retinopathy; **RR:** risk ratio; **SBP:** systolic blood pressure; **vs:** versus ^a^Males ^b^Females

**6:** Educational level ‐ Studies undertaking multivariable regression analyses to determine the effect of educational level on progression to PDR

**Study**	**Study type**	**Time years **	**N at baseline**	**Adjustment factors**	**Effect estimate**			**P value**	**Comments**
					Type	Value	95% CI		
**Type 1 diabetes**									
WESDR Klein 1994	Prospective cohort	4	996	HbA1c, DR severity at baseline	OR	0.59^a^ 0.26^b^	0.2 to 1.78 0.07 to 0.99		Per ≥ 5 years of education
**Type 2 diabetes**									
WESDR Klein 1994	Prospective cohort	4	1370	HbA1c, DR severity at baseline	OR	0.50^a^ 0.90^b^	0.21 to 1.16 0.33 to 2.48		Per ≥ 5 years of education

**BMI:** body mass index; **CI:** confidence interval; **DBP:** diastolic blood pressure; **DM:** diabetes mellitus; **DR:** diabetic retinopathy; **HbA1c:** glycated haemoglobin/haemoglobin A1c; **HR:** hazard ratio; **NPDR:** non‐proliferative diabetic retinopathy; **OR:** odds ratio; **PDR:** proliferative diabetic retinopathy; **RR:** risk ratio; **SBP:** systolic blood pressure; **vs:** versus ^a^Males ^b^Females

**Summary of findings 2:** Prognostic factors for the development and progression of PDR in people with diabetic retinopathy: systemic factors

Population: people with diabetes Outcome: progression to PDR			
** ** **Prognostic factors**	**Study results: effect estimates (95% confidence interval (CI))**	**Certainty of evidence**	**Plain text summary**
**HbA1c** (Refer to Table 9 for adjustment factors)	**T1Dand T2D** (follow‐up 2 to 24 years) **Adjusted OR** ranged from 1.11 (0.93 to 1.32) to 2.10 (1.64 to 2.69) Data from 77,075 participants in 7 studies **Adjusted RR** ranged from 1.30 (1.04 to 1.61) to 5.75 (1.54 to 21.4) Data from 5,574 participants in 4 studies **Adjusted HR** ranged from 1.09 (0.97 to 1.22; P = 0.164) to 1.43 (1.23 to 1.67) Data from 8,219 participants in 4 studies	Moderate^a^	Increased HbA1c is likely to be associated with progression to PDR
**Fasting plasma glucose** (Refer to Table 10 for adjustment factors)	**T1Dand T2D** (follow‐up 6 to 13 years) **Adjusted OR** 1.38 (95% CI not reported) Data from 4483 participants in 1 study **Adjusted HR** 0.93^§^ (0.82 to 1.06) Data from 2623 participants in 1 study **Adjusted standardised regression estimate** 0.007 (SE 0.002). Data from 927 participants in 1 study	Very low^a,b,c^	Evidence is very uncertain about the effect of fasting plasma glucose on risk of developing PDR
**Diastolic blood pressure** (Refer to Table 11 for adjustment factors)	**T1Dand T2D** (follow‐up 4 to 25 years) **Adjusted OR** ranged from 1.02 (0.93 to 1.05) to 2.50 (1.04 to 6.00) Data from 6777 participants in 4 studies. **Adjusted HR** ranged from 1.03 (1.00 to 1.05) to 1.15 (1.01 to 1.31) **Adjusted meta‐analysis HR** 1.07 (0.96 to 1.18; Tau^2^ =0.00) Data from 2724 participants in 2 studies	Very low^a,b,c^	Evidence suggesting that DBP is associated with progression to PDR is very uncertain (DBP was not an independent predictor for development of PDR when correcting for other important risk factors, including HbA1C and DR severity at baseline)
**Systolic blood pressure** (Refer to Table 12 for adjustment factors)	**T1Dand T2D** (follow‐up 4 to 25 years) **Adjusted OR** ranged from 0.91 (0.69 to 1.20) to 1.05 (95% CI not reported) Data from 6777 participants in 4 studies **Adjusted RR** 1.41 (1.17 to 1.70)^Ϯ^ Data from 3482 participants in 1 study **Adjusted HR** ranged from 1.11 (0.98 to 1.25) to 1.14 (1.04 to 1.25)^§§^	Very low^a,b,c^	Evidence suggesting that SBP is associated with progression to PDR is very uncertain (SBP was not an independent predictor for the development of PDR when correcting for other important risk factors, including HbA1C and DR severity at baseline)
**Mean arterial pressure **	**T1D** (follow‐up 6 years) **Adjusted OR** (adjusted for HbA1c, age, sex, socioeconomic status, BMI, central retinal arterial equivalent, ocular perfusion pressure) 1.35 (0.91 to 2.00) Data from 725 participants in 1 study	Very low^a,b,c^	Evidence is very uncertain about the effect of mean arterial pressure on risk of developing PDR
**Dyslipidemia** (Refer to Table 13 for adjustment factors)	**T1Dand T2D** (follow‐up 5 years) **Adjusted HR** ranged from 0.83 (0.47 to 1.47) to 0.86 (0.71 to 1.03) Data from 58,070 participants in 2 studies	Low^a,b^	Evidence suggests dyslipidaemia may not be associated with progression to PDR
**Total cholesterol** (Refer to Table 14 for adjustment factors)	**T1Dand T2D** (follow‐up 4 to 12 years) **Adjusted OR** 1.03 (95% CI not reported) Data from 4483 participants in 1 study **Adjusted RR** 1.8 (1.20 to 2.70)^*^ Data from 953 participants in 1 study **Adjusted HR** 0.93 (0.81 to 1.07). Data from 2623 participants in 1 study	Very low^a,b,c^	Evidence suggesting that total cholesterol is associated with progression to PDR is very uncertain (total cholesterol was not an independent predictor for development of PDR when correcting for other important risk factors, including HbA1C and DR severity at baseline)
**Triglycerides** (Refer to Table 15 for adjustment factors)	**T1Dand T2D** (follow‐up 7 to 24 years) **Adjusted RR (T1D)** 1.55 (1.06 to 1.95) Data from 368 participants in 1 study **Adjusted HR (T2D)** 1.01 (0.91 to 1.12) Data from 2623 participants in 1 study	Low^a,b^	Evidence suggests triglycerides may be associated with progression to PDR in T1D
**LDL** (Refer to Table 16 for adjustment factors)	**T1Dand T2D** (follow‐up 6 to 7 years) **Adjusted HR (T2D)** 0.89 (0.78 to 1.03) Data from 2623 participants in 1 study	Very low^a,b,c^	Evidence is very uncertain about the effect of LDL on risk of developing PDR
**HDL** (Refer to Table 17 for adjustment factors)	**T1Dand T2D** (follow‐up 6 to 7 years) **Adjusted HR (T2D)** 0.88 (0.76 to 1.01) Data from 2623 participants in 1 study	Very low^a,b,c^	Evidence is very uncertain about the effect of HDL on risk of developing PDR
**Nephropathy** (biomarker of renal function) (Refer to Table 18 for adjustment factors)	**T1Dand T2D** (follow‐up 5 to 8 years) **Adjusted OR** ranged from 1.58 (95% CI not reported) to 2.68 (2.09 to 3.42) Data from 76,300 participants in 2 studies **Adjusted HR** ranged from 1.29 (0.99 to 1.67) to 9.7 (8.15 to 11.5) Data from 58,070 participants in 2 studies	Very low^a,b,c^	Evidence is very uncertain about the effect of nephropathy on risk of developing PDR
**Proteinuria** (biomarker of renal function) (Refer to Table 19 for adjustment factors)	**T1Dand T2D** (follow‐up 4 to 25 years) **Adjusted OR** ranged from 0.90 (0.25 to 3.32) to 5.17 (0.49 to 54.3) Data from 3664 participants in 3 studies **Adjusted RR** 2.50 (1.1 to 5.8) Data from 953 participants in 1 study	Very low^a,b,c^	Evidence is very uncertain about the effect of proteinuria on risk of developing PDR
**Albumin excretion rate** (biomarker of renal function) (Refer to Table 20 for adjustment factors)	**T1Dand T2D** (follow‐up 5 to 7 years) **Adjusted OR (T1D)** 2.40 (1.09 to 5.29) Data from 725 participants in 1 study **Adjusted RR (T2D)** 1.34 (0.31 to 5.82) Data from 56 participants in 1 study	Low^a,b^	Evidence suggests albumin excretion rate may be associated with progression to PDR in T1D
**Albumin creatinine ratio** (biomarker of renal function) (Refer to Table 21 for adjustment factors)	**T2D** (follow‐up 6 to 8 years) **Adjusted HR** ranged from 1.22 (1.20 to 1.78) to 6.65 (3.92 to 11.29) Data from 2327 participants in 2 studies	Moderate^a^	Evidence suggests albumin creatinine ratio is likely associated with increased risk of progression to PDR in T2D
**Estimated glomular filtration rate** (biomarker of renal function) (Refer to Table 22 for adjustment factors)	**T2D** (follow‐up 4 to 8 years) **Adjusted HR** ranged from 2.55 (1.22 to 5.35) to 4.22 (1.27 to 14.07) Data from 2501 participants in 2 studies	Moderate^a^	Evidence suggests estimated glomerular filtration rate is likely associated with progression to PDR in T2D
**Creatinine** (Refer to Table 23 for adjustment factors)	**T2D** (follow‐up 4 to 8 years) **Adjusted RR** 4.8 (95% CI not reported) Data from 953 participants in 1 study **Adjusted HR** ranged from 1.11 (0.99 to 1.23) to 2.37 (1.70 to 3.29) Data from 4719 participants in 2 studies **Adjusted meta‐analysis HR** 1.61 (0.77 to 3.36; Tau^2^ = 0.28) Data from 4660 participants in 2 studies	Very low^a,b,c^	The evidence is very uncertain about the effect of creatinine on risk of developing PDR

**BMI:** body mass index; **CI:** confidence interval; **HDL:** high‐density lipoprotetin; **HR:** hazard ratio; **LDL:** low‐density lipoprotein;**OR:** odds ratio; **PDR:** proliferative diabetic retinopathy; **RR:** risk ratio; **T1D:** type 1 diabetes; **T2D:** type 2 diabetes ^a^Downgraded by one level for risk of bias: more than 80% of studies at high or unclear risk of bias ^b^Downgraded by one level for inconsistency: significant differences in effect estimates reported by studies  ^c^Downgraded by one level for imprecision: wide 95% CIs ^§^Study did not adjust for duration of DM or DR severity at baseline (Roy 2006) ^Ϯ^Study did not adjust for DR severity at baseline (Janghorbani 2000) ^§§^Study did not adjust for duration of DM or DR severity at baseline (WESDR (Report XXII)) ^*^Study did not adjust for HbA1c or DR severity at baseline (Nelson 1989)

**7:** HbA1c ‐ Studies undertaking multivariable regression analyses to determine the effect of HbA1c on progression to PDR

**Study**	**Study type**	**Time years **	**N at baseline**	**Adjustment factors**	**Effect estimate**			**P value**	**Comments**
					Type	Value	95% CI		
**Type 1 diabetes**									
Lloyd 1995	Prospective cohort	2	496	DM duration, DR severity at baseline	RR	5.75	1.54 to 21.4		Top quartile compared to other three quartiles
Klein 1984	Prospective cohort	4	996	DM duration, DR severity at baseline, age, sex	RR	1.5	1.4 to 1.8	< 0.0001	Per 1 % increase
Janghorbani 2000	Retrospective cohort	5	1349	DM duration, SBP	RR	1.83	1.4 to 2.39		≥ 11 relative to < 11%
Roy 2006	Prospective cohort	6	725	Age, hypertension, proteinuria	OR	1.32	1.22 to 1.43	< 0.001	Per 1 % increase
Porta 2001	Prospective cohort	7	2013	DM duration, DR severity at baseline, age DM diagnosis, SBP, albumin excretion rate, waist‐to‐height	Regression estimate	3.03	2.49 to 3.69	0.0001	Comparator unclear
Arfken 1998	Retrospective cohort	7	312	DR severity at baseline, race, follow‐up period	OR	1.92	1.36‐ to 2.7	0.0002	Per 2 % increase
Kullberg 1993	Prospective cohort	8	172	DM duration, age DM diagnosis, sex, hypertension					Described narratively, data not reported.
WESDR Klein 1994	Prospective cohort	10	334	DM duration, DR severity at baseline, age, sex	OR	1.9	1.7 to 2.2	< 0.0001	Per 1 % increase
WESDR Klein XVII	Prospective cohort	14	996	DR severity at baseline, hypertension, smoking, aspirin	OR	1.81	1.6 to 2.05	< 0.001	Per 1 % increase
Skrivarhaug 2006	Prospective cohort	24	368	DM duration, DR severity at baseline, age, age at DM diagnosis, hypertension, cholesterol, albumin excretion rate, smoking	RR	2.05	1.44 to 2.93	< 0.001	Unclear
WESDR Klein XXII	Prospective cohort	24	955	SBP, proteinuria, BMI	HR	1.38	1.31 to 1.46	< 0.001	Per 1 % increase
**Type 2 diabetes**									
WESDR Klein 1988	Prospective cohort	4	1370	DM duration, DR severity at baseline, age, sex	OR	1.30	1.00 to 1.60	< 0.05	Older‐onset taking insulin Per 1 % increase
Cho 2019	Retrospective cohort	4	1527	Age, estimated glomerular filtration rate	OR	1.11	0.93 to 1.32	Nonsignificant	Per 1 % increase
Kim 1998	Prospective cohort	5	228	DM duration, age, albumin excretion rate, BMI	RR	1.30	1.04 to 1.61	< 0.05	Mean HbA1c during follow‐up
Gange 2021	Retrospective cohort (electronic database)	5	71815	Age DM diagnosis, race, BMI, smoking, socioeconomic status, insulin use, comorbidities	OR	2.10	1.64 to 2.69	< 0.001	Maximum > 9% vs < 6.5%
Okudaira 2000	Prospective cohort	6	527	DBP	HR	1.43	1.23 to 1.67	0.00001	Mean HbA1c
Lee 2021	Retrospective cohort	6	2623	DR severity at baseline, age, sex, BMI	HR	1.09	0.97 to 1.22	0.164	Per one standard deviation
Kim 2014	Retrospective	6	452	DM duration, BMI	HR	1.19	1.10 to 1.46	0.005	Per unit increase
WESDR Klein 94	Prospective cohort	10	1370	DM duration, DR severity at baseline	OR	1.2^a^ 1.9^b^	1.00 to 1.50 1.50 to 2.50	0.07 < 0.0001	Per 1 % increase
Kalter‐Leibovici 1991	Prospective cohort	10	330	DM duration, socioeconomic	OR	1.9	1.4 to 2.5		10‐year HbA1c
**Type 1 and type 2 diabetes**									
Harris 2013	Retrospective cohort (electronic database)	5	4617	Age, sex, race, comorbidities	HR	1.14	1.07 to 1.21	< 0.05	With increasing HbA1c
Janghorbani 2000	Retrospective cohort	5	3482	DM duration, SBP	RR	1.33	1.13 to 1.53		≥ 11

**BMI:** body mass index; **CI:** confidence interval; **DBP:** diastolic blood pressure; **DM:** diabetes mellitus; **DR:** diabetic retinopathy; **HbA1c:** glycated haemoglobin/haemoglobin A1c; **HR:** hazard ratio; **NPDR:** non‐proliferative diabetic retinopathy; **OR:** odds ratio; **PDR:** proliferative diabetic retinopathy; **RR:** risk ratio; **SBP:** systolic blood pressure; **vs:** versus

**8:** Fasting plasma glucose ‐ Studies undertaking multivariable regression analyses to determine the effect of fasting plasma glucose on progression to PDR

**Study**	**Study type**	**Time years **	**N at baseline**	**Adjustment factors**	**Effect estimate**			**P value**	**Comments**
					Type	Value	95% CI		
**Type 2 diabetes**									
Lee 2021	Retrospective cohort	6	2623	HbA1c, DR severity at baseline, gender, and BMI	HR	0.93	0.82 to 1.06	0.26	
Lee 1992	Prospective cohort	13	927	Duration of DM, age, plasma cholesterol, SBP, and initial DM treatment	Regression estimate	0.01	Standard error: 0.002	< 0.001	
**Type 1 and 2 diabetes**									
Keen 2001	Prospective cohort	8	4483	Duration of DM, age, DBP, insulin treatment, renal disease, type of DM	OR	1.38		< 0.01	Change of 2 mmol/l

**BMI:** body mass index; **CI:** confidence interval; **DBP:** diastolic blood pressure; **DM:** diabetes mellitus; **DR:** diabetic retinopathy; **HbA1c:** glycated haemoglobin/haemoglobin A1c; **HR:** hazard ratio; **NPDR:** non‐proliferative diabetic retinopathy; **OR:** odds ratio; **PDR:** proliferative diabetic retinopathy; **RR:** risk ratio; **SBP:** systolic blood pressure; **vs:** versus

**9:** Diastolic blood pressure ‐ Studies undertaking multivariable regression analyses to determine the effect of diastolic blood pressure on progression to PDR

**Study**	**Study type**	**Time years **	**N at baseline**	**Adjustment factors**	**Effect estimate**			**P value**	**Comments**
					Type	Value	95% CI		Per increase in one year
**Type 1 diabetes**									
WESDR Klein 89	Prospective cohort	4	996	HbA1c, DR severity at baseline, age	OR	1.02	0.99 to 1.05	0.2	Higher
Roy 2006	Prospective cohort	6	725	HbA1c, proteinuria	OR	2.5	1.04 to 6.00		79 to ≥ 86 mmHg
Porta 2001	Prospective cohort	7	2013	HbA1c, DM duration, age at DM diagnosis < 12 years, waist‐to‐hip ratio	Regression estimate	1.50 1.40^a^	1.03 to 2.20 0.93 to 2.08	0.04 0.1	Comparator unclear
Grauslund 2009	Prospective cohort	25	573	HbA1c, DM duration, DR severity at baseline, age, sex, proteinuria, SBP, BMI, smoking, maculopathy	OR	1.31	0.86 to 1.99		Per 10 mmHg
**Type 2 diabetes**									
Lee 2021	Retrospective cohort	6	2623	HbA1c, DR severity at baseline, age, sex, BMI	HR	1.15	1.01 to 1.31	0.04	Per one standard deviation
Okudaira 2000	Prospective cohort	7	527	HbA1c	HR	1.03	1.00 to 1.05	0.02	Per unit increase
**Type 1 and type 2 diabetes**									
Keen 2001	Prospective cohort	8	4483	DM duration, age, sex, SBP, cholesterol, comorbidities, BMI, smoking status, insulin treatment, type of DM	OR	1.05		Nonsignificant	Per 5 mmHg increase

**BMI:** body mass index; **CI:** confidence interval; **DBP:** diastolic blood pressure; **DM:** diabetes mellitus; **DR:** diabetic retinopathy; **HbA1c:** glycated haemoglobin/haemoglobin A1c; **HR:** hazard ratio; **NPDR:** non‐proliferative diabetic retinopathy; **OR:** odds ratio; **PDR:** proliferative diabetic retinopathy; **RR:** risk ratio; **SBP:** systolic blood pressure; **vs:** versus ^a^DR severity at baseline included in model

**10:** Systolic blood pressure ‐ Studies undertaking multivariable regression analyses to determine the effect of systolic blood pressure on progression to PDR

**Study**	**Study type**	**Time years **	**N at baseline**	**Adjustment factors**	**Effect estimate**			**P value**	**Comments**
					Type	Value	95% CI		Per increase in one year
**Type 1 diabetes**									
WESDR Klein 89	Prospective cohort	4	996	HbA1c, DR severity at baseline, age	OR	1.01	0.99 to 1.03	0.4	Increasing SBP
Janghorbani 2000	Retrospective cohort	5	1349	HbA1c, DM duration	RR	1.61	1.18 to 2.20	< 0.01	> 160mmHg
WESDR Report XXII	Prospective cohort	25	996	HbA1c, proteinuria, BMI	HR	1.14	1.04 to 1.25	0.01	Per 10 mmHg
Grauslund 2009	Prospective cohort	25	573	HbA1c, DM duration, DR severity at baseline, age, sex, proteinuria, SBP, BMI, smoking, maculopathy	OR	0.91	0.69 to 1.20		Per 10 mmHg
Type 2 diabetes									
WESDR Klein 1989	Prospective cohort	4	1370	HbA1c, DR severity at baseline, age				Nonsignificant	
Lee 2021	Retrospective cohort	6	2623	HbA1c, DR severity at baseline, age, sex, BMI	HR	1.11		0.11	Per one standard deviation
Lee 1992	Prospective cohort	12	354	DM duration, age, fasting plasma glucose, cholesterol, DM treatment	Regression estimate			0.06	Per unit increase
Type 1 and 2 diabetes									
Janghorbani 2000	Retrospective cohort	5	3482	HbA1c, DM duration, proteinuria, type of DM	RR	1.41	1.17 to 1.70	<0.001	> 160mmHg
Keen 2001	Prospective cohort	8	4483	DM duration, age, sex, DBP, cholesterol, comorbidities, BMI, smoking status, insulin treatment, type of DM	OR			Nonsignificant	Per 10 mmHg increase

**BMI:** body mass index; **CI:** confidence interval; **DBP:** diastolic blood pressure; **DM:** diabetes mellitus; **DR:** diabetic retinopathy; **HbA1c:** glycated haemoglobin/haemoglobin A1c; **HR:** hazard ratio; **NPDR:** non‐proliferative diabetic retinopathy; **OR:** odds ratio; **PDR:** proliferative diabetic retinopathy; **RR:** risk ratio; **SBP:** systolic blood pressure; **vs:** versus

**11:** Dyslipidaemia ‐ Studies undertaking multivariable regression analyses to determine the effect of dyslipidaemia on progression to PDR

**Study**	**Study type**	**Time years **	**N at baseline**	**Adjustment factors**	**Effect estimate**			**P value**	**Comments**
					Type	Value	95% CI		
**Type 1 and 2 diabetes**									
Harris 2013	Electronic database	5	4617	HbA1c, age, sex, race, comorbidities, medications	HR	0.83	0.47 to 1.47		Presence of dyslipidaemia
Jeng 2016	Electronic database	5	53,453	Age, gender, hypertension, diabetic nephropathy, comorbidities, medications	HR	0.86	0.71 to 1.03		Presence of dyslipidaemia

**BMI:** body mass index; **CI:** confidence interval; **DBP:** diastolic blood pressure; **DM:** diabetes mellitus; **DR:** diabetic retinopathy; **HbA1c:** glycated haemoglobin/haemoglobin A1c; **HR:** hazard ratio; **NPDR:** non‐proliferative diabetic retinopathy; **OR:** odds ratio; **PDR:** proliferative diabetic retinopathy; **RR:** risk ratio; **SBP:** systolic blood pressure; **vs:** versus

**12:** Total cholesterol ‐ Studies undertaking multivariable regression analyses to determine the effect of total cholesterol on progression to PDR

**Study**	**Study type**	**Time years **	**N at baseline**	**Adjustment factors**	**Effect estimate**			**P value**	**Comments**
					Type	Value	95% CI		Per increase in one year
**Type 1 diabetes**									
Porta 2001	Prospective cohort	7	2013	HbA1c, DM duration				Reported narratively as nonsignificant	
**Type 2 diabetes**									
Nelson 1989	Prospective cohort	4	953	DM duration, age, sex	RR	1.80	1.2 to 2.7		≥ 4.8 vs < 4.8 mM
Lee 2021	Retrospective cohort	6	2623	HbA1c, DR severity at baseline, age, sex, BMI	HR	0.93	0.81 to 1.07	0.31	Per one standard deviation
Lee 1992	Prospective cohort	12	354	HbA1c, DM duration, age, SBP, DM treatment	Regression estimate	0.006	Standard error: 0.003	0.05	Per unit increase
**Type 1 and type 2 diabetes**									
Keen 2001	Prospective cohort	8	4483	Age, sex, SBP, DBP, cholesterol, BMI, smoking status, insulin, type of DM, comorbidities	OR	1.03		< 0.01	Per 10 mg/dL

**BMI:** body mass index; **CI:** confidence interval; **DBP:** diastolic blood pressure; **DM:** diabetes mellitus; **DR:** diabetic retinopathy; **HbA1c:** glycated haemoglobin/haemoglobin A1c; **HR:** hazard ratio; **NPDR:** non‐proliferative diabetic retinopathy; **OR:** odds ratio; **PDR:** proliferative diabetic retinopathy; **RR:** risk ratio; **SBP:** systolic blood pressure; **vs:** versus

**13:** Triglycerides ‐ Studies undertaking multivariable regression analyses to determine the effect of triglycerides on progression to PDR

**Study**	**Study type**	**Time years **	**N at baseline**	**Adjustment factors**	**Effect estimate**			**P value**	**Comments**
					Type	Value	95% CI		
									
**Type 1**									
Porta 2001	Prospective cohort	7	2013	HbA1c, DM duration				Reported narratively as nonsignificant	
Skrivarhaug 2006	Prospective cohort	24	368	HbA1c, DM duration, DR severity at baseline, age, age DM diagnosis, sex, smoking status, hypertension, cholesterol, albumin excretion rate	RR	1.55	1.06 to 1.95	0.02	With increasing triglyceride level
**Type 2 diabetes**									
Lee 2021	Retrospective cohort	6	2623	HbA1c, DR severity at baseline, age, sex, BMI	HR	1.01	0.91 to 1.12	0.88	Per one standard deviation

**BMI:** body mass index; **CI:** confidence interval; **DBP:** diastolic blood pressure; **DM:** diabetes mellitus; **DR:** diabetic retinopathy; **HbA1c:** glycated haemoglobin/haemoglobin A1c; **HR:** hazard ratio; **NPDR:** non‐proliferative diabetic retinopathy; **OR:** odds ratio; **PDR:** proliferative diabetic retinopathy; **RR:** risk ratio; **SBP:** systolic blood pressure; **vs:** versus

**14:** Low‐density lipoprotein (LDL) ‐ Studies undertaking multivariable regression analyses to determine the effect of LDL on progression to PDR

**Study**	**Study type**	**Time years **	**N at baseline**	**Adjustment factors**	**Effect estimate**			**P value**	**Comments**
					Type	Value	95% CI		
									
**Type 1**									
Porta 2001	Prospective cohort	7	2013	HbA1c, DM duration				Reported narratively as nonsignificant	
**Type 2 diabetes**									
Lee 2021	Retrospective cohort	6	2623	HbA1c, DR severity at baseline, age, sex, BMI	HR	0.89	0.78 to 1.03	0.12	Per one standard deviation

**BMI:** body mass index; **CI:** confidence interval; **DBP:** diastolic blood pressure; **DM:** diabetes mellitus; **DR:** diabetic retinopathy; **HbA1c:** glycated haemoglobin/haemoglobin A1c; **HR:** hazard ratio; **NPDR:** non‐proliferative diabetic retinopathy; **OR:** odds ratio; **PDR:** proliferative diabetic retinopathy; **RR:** risk ratio; **SBP:** systolic blood pressure; **vs:** versus

**15:** High‐density lipoprotein (HDL) ‐ Studies undertaking multivariable regression analyses to determine the effect of HDL on progression to PDR

**Study**	**Study type**	**Time years **	**N at baseline**	**Adjustment factors**	**Effect estimate**			**P value**	**Comments**
					Type	Value	95% CI		
									
**Type 1**									
Porta 2001	Prospective cohort	7	2013	HbA1c, DM duration				Reported narratively as nonsignificant	
**Type 2 diabetes**									
Lee 2021	Retrospective cohort	6	2623	HbA1c, DR severity at baseline, age, sex, BMI	HR	0.88	0.76 to 1.01	0.07	Per one standard deviation

**BMI:** body mass index; **CI:** confidence interval; **DBP:** diastolic blood pressure; **DM:** diabetes mellitus; **DR:** diabetic retinopathy; **HbA1c:** glycated haemoglobin/haemoglobin A1c; **HR:** hazard ratio; **NPDR:** non‐proliferative diabetic retinopathy; **OR:** odds ratio; **PDR:** proliferative diabetic retinopathy; **RR:** risk ratio; **SBP:** systolic blood pressure; **vs:** versus

**16:** Nephropathy ‐ Studies undertaking multivariable regression analyses to determine the effect of nephropathy on progression to PDR

**Study**	**Study type**	**Time years **	**N at baseline**	**Adjustment factors**	**Effect estimate**			**P value**	**Comments**
					Type	Value	95% CI		
									
**Type 2 diabetes**									
Gange 2021	Prospective cohort (electronic database)	5	71,817	HbA1c, DM duration	OR	2.68	2.09 to 3.42	< 0.001	
**Type 1 and 2 diabetes**									
Harris 2013	Electronic database	5	4617	Age, sex, race, comorbidities, medications	HR	1.29	0.99 to 1.67	> 0.05	Presence
Jeng 2016	Electronic database	5	53,453	Age, sex, comorbidities, medications	HR	9.7	8.15 to 11.5	< 0.001	Presence
Keen 2001	Prospective cohort	8	4483	Sex, age, duration of DM, SBP, DBP, cholesterol, BMI, smoking status, insulin treatment, vascular disease, type of DM	OR	1.58 1.62^a^		< 0.01 < 0.05	Presence

**BMI:** body mass index; **CI:** confidence interval; **DBP:** diastolic blood pressure; **DM:** diabetes mellitus; **DR:** diabetic retinopathy; **HbA1c:** glycated haemoglobin/haemoglobin A1c; **HR:** hazard ratio; **NPDR:** non‐proliferative diabetic retinopathy; **OR:** odds ratio; **PDR:** proliferative diabetic retinopathy; **RR:** risk ratio; **SBP:** systolic blood pressure; **vs:** versus ^a^Fasting plasma glucose also included as covariate

**17:** Proteinuria ‐ Studies undertaking multivariable regression analyses to determine the effect of proteinuria on progression to PDR

**Study**	**Study type**	**Time years **	**N at baseline**	**Adjustment factors**	**Effect estimate**			**P value**	**Comments**
					Type	Value	95% CI		
									
**Type 1 diabetes**									
WESDR Klein 1993	Prospective cohort	4	996	HbA1, DBP	OR	2.76^a^ 1.51^b^	0.99 to 7.68 0.48 to 4.77	0.05 0.48	Gross proteinuria present
Roy 2006	Prospective cohort	6	725	HbA1c, age, hypertension	OR	1.00^c^ 3.74^d^	1.52 to 9.18	0.01	
WESDR Report XVII	Prospective cohort	14	996	DR severity at baseline,	OR	1.65	1.03 to 2.64		No vs yes
WESDR Report XXII	Prospective cohort	25	996	HbA1c, SBP, BMI	HR	1.83	1.31 to 2.56	< 0.001	No vs yes
**Type 2 diabetes**									
WESDR Klein 1993	Prospective cohort	4	1370	HbA1, DBP	OR	0.90	0.25 to 3.32	0.88	Older‐onset group taking insulin Gross proteinuria present
Nelson 1989	Prospective cohort	4	953	DM duration, age, sex	RR	2.50	Range: 1.1 to 5.8		No vs yes Proteinuria: urine protein‐to‐creatinine ratio ≥ 113 mg/mmol
Grauslund 2009	Prospective cohort	25	573	HbA1c, DR severity at baseline, age, sex, DBP, SBP, BMI, proteinuria, smoking, maculopathy	OR	5.17	0.49 to 54.3		Proteinuria vs no proteinuria
**Type 1 and 2 diabetes**									
Janghorbani 2000	Retrospective cohort	5	3482	HbA1c, DM duration, SBP, type of DM	RR	1.00^e^ 1.27^f^	1.05 to 1.54	< 0.05	

**BMI:** body mass index; **CI:** confidence interval; **DBP:** diastolic blood pressure; **DM:** diabetes mellitus; **DR:** diabetic retinopathy; **HbA1c:** glycated haemoglobin/haemoglobin A1c; **HR:** hazard ratio; **NPDR:** non‐proliferative diabetic retinopathy; **OR:** odds ratio; **PDR:** proliferative diabetic retinopathy; **RR:** risk ratio; **SBP:** systolic blood pressure; **vs:** versus ^a^No/mild NPDR at baseline ^b^Moderate/severe NPDR at baseline ^c^No proteinuria ^d^Overt proteinuria ^e^No proteinuria ^f^Proteinuria

**18:** Albumin excretion rate ‐ Studies undertaking multivariable regression analyses to determine the effect of albumin excretion rate on progression to PDR

**Study**	**Study type**	**Time years **	**N at baseline**	**Adjustment factors**	**Effect estimate**			**P value**	**Comments**
					Type	Value	95% CI		
**Type 1 diabetes**									
Roy 2006	Prospective cohort	6	725	HbA1c, age, hypertension	OR	2.40	1.09 to 5.29	0.009	Microproteinuria
Porta 2001	Prospective cohort	7	2013	HbA1c, DM duration, DR severity at baseline, age of DM diagnosis < 12 years, DBP, waist‐to‐height ratio	Regression estimate	1.33	1.12 to 1.58	0.001	With increasing level
**Type 2 diabetes**									
Kim 1998	Prospective cohort	5	56	HbA1c, DM duration, age, change in BMI	RR	1.34	0.31 to 5.82		With increasing level

**BMI:** body mass index; **CI:** confidence interval; **DBP:** diastolic blood pressure; **DM:** diabetes mellitus; **DR:** diabetic retinopathy; **HbA1c:** glycated haemoglobin/haemoglobin A1c; **HR:** hazard ratio; **NPDR:** non‐proliferative diabetic retinopathy; **OR:** odds ratio; **PDR:** proliferative diabetic retinopathy; **RR:** risk ratio; **SBP:** systolic blood pressure; **vs:** versus

**19:** Albumin creatinine ratio ‐ Studies undertaking multivariable regression analyses to determine the effect of albumin creatinine ratio on progression to PDR

**Study**	**Study type**	**Time years **	**N at baseline**	**Adjustment factors**	**Effect estimate**			**P value**	**Comments**
					Type	Value	95% CI		
**Type 2 diabetes**									
Kim 2014	Retrospective cohort	6	231	HbA1c, DM duration	HR	1.22	1.20 to 1.78	0.004	Per unit increase
Hsieh 2018	Prospective cohort	8	2096	DM duration, age, sex, SBP, BMI, serum fasting glucose, cholesterol, low and high density lipoprotein, triglycerides	HR	3.20^a^ 6.65^b^	2.03 to 5.05 3.92 to 11.29	< 0.001 < 0.001	

**BMI:** body mass index; **CI:** confidence interval; **DBP:** diastolic blood pressure; **DM:** diabetes mellitus; **DR:** diabetic retinopathy; **HbA1c:** glycated haemoglobin/haemoglobin A1c; **HR:** hazard ratio; **NPDR:** non‐proliferative diabetic retinopathy; **OR:** odds ratio; **PDR:** proliferative diabetic retinopathy; **RR:** risk ratio; **SBP:** systolic blood pressure; **vs:** versus ^a^31‐300mg/g vs. <10mg/g ^b^>300mg/g vs. <10mg/g

**20:** Estimated glomerular filtration rate (eGFR) ‐ Studies undertaking multivariable regression analyses to determine the effect of eGFR on progression to PDR

**Study**	**Study type**	**Time years **	**N at baseline**	**Adjustment factors**	**Effect estimate**			**P value**	**Comments**
					Type	Value	95% CI		
**Type 2 diabetes**									
Cho 2019	Retrospective cohort	4	405	HbA1c, age	HR	2.55	1.22 to 5.35	< 0.05	a reduction in eGFR of > 20%
Hsieh 2018	Prospective cohort	8	2096	DM duration, age, sex, SBP, BMI, serum fasting glucose, cholesterol, low‐ and high‐density lipoprotein, triglycerides	HR	1.55^a^ 2.05^b^ 4.22^c^	0.63 to 3.82 0.72 to 5.86 1.27 to 14.07	0.34 0.18 0.02	

**BMI:** body mass index; **CI:** confidence interval; **DBP:** diastolic blood pressure; **DM:** diabetes mellitus; **DR:** diabetic retinopathy; **HbA1c:** glycated haemoglobin/haemoglobin A1c; **HR:** hazard ratio; **NPDR:** non‐proliferative diabetic retinopathy; **OR:** odds ratio; **PDR:** proliferative diabetic retinopathy; **RR:** risk ratio; **SBP:** systolic blood pressure; **vs:** versus ^a^46‐60mL/min/1.73m^2^ ^b^30‐45mL/min/1.73m^2^ c<30 mL/min/1.73m^2^

**21:** Creatinine ‐ Studies undertaking multivariable regression analyses to determine the effect of creatinine on progression to PDR

**Study**	**Study type**	**Time years **	**N at baseline**	**Adjustment factors**	**Effect estimate**			**P value**	**Comments**
					Type	Value	95% CI		
**Type 2 diabetes**									
Nelson 1989	Electronic database	4	953	DM duration, age, sex	RR	4.80	Range: 1.3 to 17.6		serum creatinine concentration of ≥ 177µM (2.0 mg/dL)
Hsieh 2018	Prospective cohort	8	2096	DM duration, age, sex, SBP, BMI, serum fasting glucose, cholesterol, HDL, LDL, triglycerides	HR	2.36^a^ 2.37^b^	1.90 to 2.92 1.70 to 3.29	< 0.001 < 0.001	
Lee 2021	Retrospective cohort	6	2623	HbA1c, DR severity at baseline, age, sex, BMI	HR	1.11	0.99 to 1.23	0.06	Per one standard deviation

**BMI:** body mass index; **CI:** confidence interval; **DBP:** diastolic blood pressure; **DM:** diabetes mellitus; **DR:** diabetic retinopathy; **HbA1c:** glycated haemoglobin/haemoglobin A1c; **HR:** hazard ratio; **NPDR:** non‐proliferative diabetic retinopathy; **OR:** odds ratio; **PDR:** proliferative diabetic retinopathy; **RR:** risk ratio; **SBP:** systolic blood pressure; **vs:** versus ^a^At baseline ^b^During follow‐up

**Summary of findings 3:** Prognostic factors for the development and progression of PDR in people with diabetic retinopathy: ocular factors

Population: people with diabetes Outcome: progression to PDR			
**Prognostic factors**	**Study results: effect estimates (95% confidence interval (CI))**	**Certainty** **of evidence**	**Plain text summary**
**DR severity at baseline** (Refer to Table 25 for adjustment factors)	**T1Dand T2D** (follow‐up 1 to 25 years) **Adjusted OR** ranged from 1.38 (1.29 to 1.48) to 12.40 (5.31 to 28.98) Data from 3321 participants in 3 studies **Adjusted RR** 5.99 (3.03 to 11.9) Data from 322 participants in 1 study **Adjusted HR** ranged from 23.09 (10.68 to 49.91) to 14.80 (12.10 to 18.09). Data from 35,176 participants in 2 studies	Moderate^a^	Evidence suggests DR severity at baseline is likely associated with risk of progression to PDR
**DR features at baseline** (Refer to Table 26 for adjustment factors)	**T1Dand T2D** (follow‐up 4 to 5 years) **Adjusted HR** 1.77^§^ (1.25 to 2.49) 1.47^*^ (0.94 to 2.31) Data from 2823 participants in 1 study **Adjusted OR** 1.04^Ϯ^ (1.02 to 1.07) 1.05^§§^ (1.01 to 1.09) 5.77**(2.24 to 14.89) Data from 236 participants in 1 study	Very low^a,b,c^	Evidence is very uncertain about the effect of DR features at baseline on risk of developing PDR
**Retinal vessel caliber** (Refer to Table 27 for adjustment factors)	**T1Dand T2D** (follow‐up 6 to 14 years) **Adjusted OR (T1D)** 3.49^ϮϮ^ (1.44 to 8.46) **Adjusted RR (T1D)** 4.28^¶^ (1.50 to 12.19) **Adjusted HR (T2)** 1.17^¶^ (0.68 to 2.04)	Low^a,b^	Evidence suggests larger central retinal venular diameter may be associated with increased risk of progression to PDR in T1D
**Intra‐ocular pressure** (Refer to** **Table 28 for adjustment factors)	**T1Dand T2D** (follow‐up 4 years) **Adjusted OR (T1)** 1.04 (0.96 to 1.13); **(T2)** 0.95 (0.85 to 1.08)** **	Very low^a,b,c^	Evidence is very uncertain about the effect of intra‐ocular pressure on risk of developing PDR

**CI:** confidence interval; **HR:** hazard ratio; **OR:** odds ratio; **PDR:** proliferative diabetic retinopathy; **RR:** risk ratio; **T1D:** type 1 diabetes; **T2D:** type 2 diabetes ^a^Downgraded one level for risk of bias: more than 80% of studies at high or unclear risk of bias ^b^Downgraded by one level for inconsistency: significant differences in effect estimates reported by studies ^c^Downgraded by one level for imprecision: wide 95% CIs ^§^Intraretinal microvascular abnormalities (IRMA) vs. venous beading in four quadrants ^*^Dot/blot haemorrhages vs. venous beading in four quadrants ^Ϯ^Difference in number of microaneurysms at baseline and follow‐up ^§§^Ratio between number of microaneurysms at baseline and follow‐up ^**^Difference of ≥ 16 microaneurysms at baseline and follow‐up ^ϮϮ^Central retinal venular equivalent ≥ 272.27 vs ≤ 235.97 ^¶^Larger retinal venular equivalent

**22:** Diabetic retinopathy severity at baseline ‐ Studies undertaking multivariable regression analyses to determine the effect of diabetic retinopathy severity at baseline on progression to PDR

**Study**	**Study Type**	**Time years **	**N at baseline**	**Adjustment factors**	**Effect estimate**			**P value**	**Comments**
					Type	Value	95% CI		
**Type 1 diabetes**									
Lloyd 1995	Prospective cohort	1	322	HbA1c, DM duration	RR	5.99	3.03 to 11.9		Worsening baseline severity, unclear how grouped
Porta 2001	Prospective cohort	7	2013	HbA1c, DM duration, age of DM diagnosis < 12 years, DBP, albumin excretion rate, waist‐to‐heigh ratio	OR	10.1	5.9 to 17.2	< 0.0001	Worsening baseline severity
WESDR Report XVII	Prospective cohort	14	996	HbA1c, hypertension, smoking, aspirin	OR	1.38	1.29 to 1.48	< 0.001	Worsening baseline severity
**Type 2 diabetes**									
Lee 2021	Retrospective cohort	6	2623	HbA1c, age, sex, BMI	HR	13.58^c^ 23.09^d^ 55.24^e^	6.07 to 30.39 10.68 to 49.91 25.54 to 119.46	< 0.001 < 0.001 < 0.001	Mean
Arfken 1998	Retrospective cohort	7	312	Race, follow‐up schedule	OR	12.4	5.31 to 28.98	0.0001	
**Type 1 and type 2 diabetes**									
Lee 2017	Registry database	5	32553	Age, sex, race, baseline visual acuity	HR	1.00^a^ 4.02^b^ 6.71^c^ 14.80^d^ 28.19^e^ 58.42^f^	3.25 to 4.96 5.46 to 8.24 12.10 to 18.09 22.92 to 34.67 46.95 to 72.70	< 2x10^‐16^ < 2x10^‐16^ < 2x10^‐16^ < 2x10^‐16^ < 2x10^‐16^	

**BMI:** body mass index; **CI:** confidence interval; **DBP:** diastolic blood pressure; **DM:** diabetes mellitus; **DR:** diabetic retinopathy; **HbA1c:** glycated haemoglobin/haemoglobin A1c; **HR:** hazard ratio; **NPDR:** non‐proliferative diabetic retinopathy; **OR:** odds ratio; **PDR:** proliferative diabetic retinopathy; **RR:** risk ratio; **SBP:** systolic blood pressure; **vs:** versus ^a^No NPDR ^b^Very mild NPDR ^c^Mild NPDR ^d^Mod NPDR ^e^Sev NPDR ^f^Very sev

**23:** Diabetic retinopathy features at baseline ‐ Studies undertaking multivariable regression analyses to determine the effect of diabetic retinopathy features at baseline on progression to PDR

**Study**	**Study type**	**Time years **	**N at baseline**	**Adjustment factors**	**Effect estimate**			**P value**	**Comments**
					Type	Value	95% CI		
**Type 1 and 2 diabetes**									
Lee 2017	Electronic database	5	2823	Age, sex, race, initial visual acuity	HR	1.77^a^ 1.47^b^	1.25 to 2.49 0.94 to 2.31	0.001 0.88	
WESDR Klein 1995	Prospective cohort	4	236	HbA1c, duration of DM, age, sex, age at DM diagnosis, SBP, DBP, BMI, proteinuria and type of DM	OR	1.04^c^ 1.05^d^ 5.77^e^	1.02 to 1.07 1.01 to 1.09 2.24 to 14.89	< 0.001 0.006 < 0.001	

**BMI:** body mass index; **CI:** confidence interval; **DBP:** diastolic blood pressure; **DM:** diabetes mellitus; **DR:** diabetic retinopathy; **HbA1c:** glycated haemoglobin/haemoglobin A1c; **HR:** hazard ratio; **NPDR:** non‐proliferative diabetic retinopathy; **OR:** odds ratio; **PDR:** proliferative diabetic retinopathy; **RR:** risk ratio; **SBP:** systolic blood pressure; **vs:** versus ^a^IRMA vs venous beading in four quadrants ^b^Dot/blot haemorrhages vs venous beading in four quadrants ^c^Difference in number of microaneurysms at baseline and follow‐up ^d^Ratio between number of microaneurysms at baseline and follow‐up ^e^Difference of ≥ 16 microaneurysms at baseline and follow‐up

**24:** Retinal vessel caliber ‐ Studies undertaking multivariable regression analyses to determine the effect of retinal vessel caliber at baseline on progression to PDR

**Study**	**Study type**	**Time years **	**N at baseline**	**Adjustment factors**	**Effect estimate**			**P value**	**Comments**
					Type	Value	95% CI		
**Type 1 diabetes**									
Roy 2006	Prospective cohort	6	725	HbA1c, age, sex, BMI, socioeconomic status, proteinuria, central retinal artery equivalent, ocular perfusion pressure, refractive error.	OR	3.49	1.44 to 8.46	0.03	Central retinal vein equivalent ≥ 272.27 vs ≤ 235.97
WESDR Report XIX	Prospective cohort	10, 14	996	HbA1c, DM duration, DR severity at baseline, sex, mean arterial pressure, anti‐BP medication	RR	4.28	1.50 to 12.19	0.006	Larger venular diameters
**Type 2 diabetes**									
WESDR Report XXI	Prospective cohort	10	962	HbA1c, DR severity at baseline, age	HR	1.17^a^ 0.91^b^	0.68 to 2.04 0.46 to 1.80	0.57 0.78	

**BMI:** body mass index; **CI:** confidence interval; **DBP:** diastolic blood pressure; **DM:** diabetes mellitus; **DR:** diabetic retinopathy; **HbA1c:** glycated haemoglobin/haemoglobin A1c; **HR:** hazard ratio; **NPDR:** non‐proliferative diabetic retinopathy; **OR:** odds ratio; **PDR:** proliferative diabetic retinopathy; **RR:** risk ratio; **SBP:** systolic blood pressure; **vs:** versus ^a^Larger central retinal vein equivalent ^b^Larger central retinal artery equivalent

**25:** Intra‐ocular pressure ‐ Studies undertaking multivariable regression analyses to determine the effect of intra‐ocular pressure at baseline on progression to PDR

**Study**	**Study type**	**Time years **	**N at baseline**	**Adjustment factors**	**Effect estimate**			**P value**	**Comments**
					Type	Value	95% CI		
**Type 1 diabetes**									
WESDR Moss 1993	Prospective cohort	4	996	HbA1c, DR severity, and age at baseline.	OR	1.04	0.96 to 1.13		
**Type 2 diabetes**									
WESDR Moss 1993	Prospective cohort	4	674	HbA1c, duration of DM, and DR severity at baseline	OR	0.95	0.83 to 1.08		

**BMI:** body mass index; **CI:** confidence interval; **DBP:** diastolic blood pressure; **DM:** diabetes mellitus; **DR:** diabetic retinopathy; **HbA1c:** glycated haemoglobin/haemoglobin A1c; **HR:** hazard ratio; **NPDR:** non‐proliferative diabetic retinopathy; **OR:** odds ratio; **PDR:** proliferative diabetic retinopathy; **RR:** risk ratio; **SBP:** systolic blood pressure; **vs:** versus

**Summary of findings 4:** Prognostic factors for the development and progression of PDR in people with diabetic retinopathy: lifestyle factors

Population: people with diabetes Outcome: progression to PDR			
**Prognostic factors**	**Study results: effect estimates (95% confidence interval (CI))**	**Certainty** **of evidence**	**Plain text summary**
**Body mass index** (Refer to Table 30 for adjustment factors)	**T1Dand T2D** (follow‐up 4 to 25 years) **Adjusted OR** ranged from 1.01 (0.86 to 1.20) to 1.05 (95% CI not reported) Data from 5056 participants in 2 studies **Adjusted RR** ranged from 1.00 (95% CI not reported) to 1.41 (0.76 to 2.62). Data from 2379 participants in 2 studies **Adjusted HR** ranged from 0.91 (0.79 to 1.03) to 1.21 (1.07 to 1.36). Data from 3619 participants in 2 studies	Very low^a,b,c^	Evidence is very uncertain about the effect of body mass index on risk of developing PDR
**Smoking status** (Refer to Table 31 for adjustment factors)	**T1Dand T2D** (follow‐up 4 to 14 years) **Adjusted OR** ranged from 0.25 (0.03 to 2.06) to 1.90 (0.88 to 4.11) Data from 79,247 participants in 2 studies **Adjusted RR** 0.70 (0.20 to 1.90) Data from 953 participants in 1 study	Very low^a,b,c^	Evidence is very uncertain about the effect of smoking status on risk of developing PDR
**Alcohol consumption** (Refer to Table 32 for adjustment factors)	**T1Dand T2D** (follow‐up 4 years) **Adjusted OR (T1)** 0.72 (0.38 to 1.35) Data from 996 participants in 1 study **Adjusted OR (T2)** 1.10 (0.56 to 3.41) Data from 1370 participants in 1 study	Very low^a,b,c^	Evidence is very uncertain about the effect of alcohol consumption on risk of developing PDR

**CI:** confidence interval; **HR:** hazard ratio; **OR:** odds ratio; **PDR:** proliferative diabetic retinopathy; **RR:** risk ratio; **T1D:** type 1 diabetes; **T2D:** type 2 diabetes ^a^Downgraded by one level for risk of bias: more than 80% of studies at high or unclear risk of bias ^b^Downgraded by one level for inconsistency: significant differences in effect estimates reported by studies ^c^Downgraded by one level for imprecision: wide 95% CIs

**26:** Body mass index (BMI) ‐ Studies undertaking multivariable regression analyses to determine the effect of BMI on progression to PDR

**Study**	**Study type**	**Time years **	**N at baseline**	**Adjustment factors**	**Effect estimate**			**P value**	**Comments**
					Type	Value	95% CI		Per increase in one year
**Type 1 diabetes**									
Grauslund 2009	Prospective cohort	25	573	HbA1c, DR severity at baseline, age, sex, SBP, DBP, proteinuria, maculopathy	OR	1.01	0.86‐1.20		per increase in one kg/m^2^
WESDR Report XXII	Prospective cohort	25	996	HbA1c, SBP, proteinuria	HR	1.21	1.07 to 1.36	0.002	per increase in four kg/m^2^
**Type 2 diabetes**									
WESDR Klein 1997	Prospective cohort	4	1370	DR severity at baseline, insulin use	RR	1.41	0.76 to 2.62		BMI = obesity at baseline (men: > 31.0 kg/m^2^; women: > 32.2 kg/m^2^
Nelson 1989	Prospective cohort	4	953	DM duration, age, sex	RR	1.0	Range: 0.6 to 1.6		≥34 vs. < 34 kg/m^2^
Kim 1998	Prospective cohort	5	56	HbA1c, DM duration, age	RR	1.33	0.87 to 1.50		Change in BMI during follow‐up
Lee 2021	Retrospective cohort	6	2623	HbA1c, DR severity at baseline, age, sex	HR	0.91	0.79 to 1.03		Per one standard deviation
**Type 1 and type 2 diabetes**									
Keen 2001	Prospective cohort	8	4483	DM duration, age, sex, SBP, DBP, insulin use, cholesterol, BMI, fasting plasma glucose, smoking status, comorbidities, type of DM	OR	1.05		Nonsignificant	8 to 11 years vs ≥ 12 years

**BMI:** body mass index; **CI:** confidence interval; **DBP:** diastolic blood pressure; **DM:** diabetes mellitus; **DR:** diabetic retinopathy; **HbA1c:** glycated haemoglobin/haemoglobin A1c; **HR:** hazard ratio; **NPDR:** non‐proliferative diabetic retinopathy; **OR:** odds ratio; **PDR:** proliferative diabetic retinopathy; **RR:** risk ratio; **SBP:** systolic blood pressure; **vs:** versus

**27:** Smoking ‐ Studies undertaking multivariable regression analyses to determine the effect of smoking on progression to PDR

**Study**	**Study Type**	**Time years **	**N at baseline**	**Adjustment factors**	**Effect estimate**			**P value**	**Comments**
					Type	Value	95% CI		
									
**Type 1 diabetes**									
WESDR Moss 1991	Prospective cohort	4	799	HbA1c, DM duration, DR severity at baseline, age, sex	OR	1.15	0.6 to 2.2		Ever vs never
WESDR Moss 1996	Prospective cohort	10	799	HbA1c, DM duration, DR severity at baseline, age, sex	OR	0.86^a^ 0.94^b^	0.54 to 1.36 0.51 to 1.75		
Grauslund 2009 (Thorlund)	Prospective cohort	5	573	HbA1c, DM duration, age, sex, SBP, DBP, comorbidities	OR	1.9^a^ 0.87^b^	0.88 to 4.11 0.28 to 2.67		
WESDR WESDR XVII	Prospective cohort	14	996	HbA1c, DR severity at baseline, BP, aspirin	OR	0.79	0.66 to 0.95	< 0.05	Diabetic pack years smoked per 10 years
**Type 2 diabetes**									
Gui 2013	Retrospective cohort	2	205	DM duration, age, BP, C‐peptide	OR	1.07	1.04 to 1.11	< 0.05	% smokers vs non‐smokers
Nelson 1989	Prospective cohort	4	953	DM duration, age, sex	RR	0.70	0.2 to 1.9		Smoking: yes vs no
WESDR Moss 1991	Prospective cohort	4	1370	HbA1c, DM duration, DR severity at baseline	OR	1.13	0.45 to 7.83		Ever vs never
Gange 2021	Electronic database	5	71,817	HbA1c, age at DM diagnosis, race, comorbidities, income, insulin use	OR	0.84	0.7 to 1.0		Smoking
WESDR Moss 1996	Prospective cohort	10	1370	HbA1c, DM duration, DR severity at baseline	OR	Insulin 1.04^b^ 1.15^a^ Non‐insulin 0.8^b^ 0.25^a^	0.49 to 2.22 0.47 to 2.8 0.23 to 2.8 0.03 to 2.06		
Keen 2001	Prospective cohort	8	4483	DM duration, age, sex, SBP, DBP, co‐morbidities, insulin use, type of DM	OR	0.67		< 0.01	No vs yes

**BMI:** body mass index; **CI:** confidence interval; **DBP:** diastolic blood pressure; **DM:** diabetes mellitus; **DR:** diabetic retinopathy; **HbA1c:** glycated haemoglobin/haemoglobin A1c; **HR:** hazard ratio; **NPDR:** non‐proliferative diabetic retinopathy; **OR:** odds ratio; **PDR:** proliferative diabetic retinopathy; **RR:** risk ratio; **SBP:** systolic blood pressure; **vs:** versus ^a^Current smoker ^b^Ex‐smoker

**28:** Alcohol consumption ‐ Studies undertaking multivariable regression analyses to determine the effect of alcohol consumption on progression to PDR

**Study**	**Study type**	**Time years **	**N at baseline**	**Adjustment factors**	**Effect estimate**			**P value**	**Comments**
					Type	Value	95% CI		
**Type 1 diabetes**									
WESDR Moss 1994	Prospective cohort	4	996	HbA1c, DR severity at baseline.	OR	0.72^a^ 1.02^b^	0.38 to 1.35 0.56 to 1.86		
**Type 2 diabetes**									
WESDR Moss 1994	Prospective cohort	4	1370	HbA1c, DR severity at baseline.	OR	1.10^a^ 1.55^b^	0.36 to 3.41 0.73 to 3.30		

**BMI:** body mass index; **CI:** confidence interval; **DBP:** diastolic blood pressure; **DM:** diabetes mellitus; **DR:** diabetic retinopathy; **HbA1c:** glycated haemoglobin/haemoglobin A1c; **HR:** hazard ratio; **NPDR:** non‐proliferative diabetic retinopathy; **OR:** odds ratio; **PDR:** proliferative diabetic retinopathy; **RR:** risk ratio; **SBP:** systolic blood pressure; **vs:** versus ^a^Average ^b^Recent

## Background

### Description of the health condition and context

#### Health condition

Diabetes mellitus (DM) is a chronic metabolic disease characterised by elevated blood glucose levels which, over time, lead to multiorgan dysfunction. In 2021, the International Diabetes Federation estimated that 537 million adults globally were living with diabetes (IDF 2021). It estimates this figure will rise to 643 million people by 2030, due to population expansion and ageing, urbanisation, increasing levels of obesity, inadequate nutrition, and sedentary lifestyles (IDF 2021; Saeedi 2019). Diabetic retinopathy (DR) occurs because of neurovascular degeneration triggered by hyperglycaemia, and is the most common microvascular complication of diabetes. Worldwide prevalence of retinopathy related to diabetes, including diabetic macular oedema, was recently determined to be 27% in the period 2015 to 2019 (Thomas 2019). However, in their review, Thomas and colleagues report limitations in determining a more precise estimate due to differences in study populations and methodology.

DR is a progressive condition with advancing levels of severity. A classification in stages based on DR microvascular features, as observed on fundus photographs, was proposed by the Early Treatment Diabetic Retinopathy Study (ETDRS) Group. As a result, DR is categorised into two main stages: non‐proliferative (NPDR), and the more serious, sight‐threatening, proliferative stage (PDR) (ETDRS 1991a). The earliest visible clinical signs of NPDR are microhaemorrhages and microaneurysms which represent damage to retinal capillaries. Mild NPDR is defined by the presence of at least one retinal microaneurysm or microhaemorrhage. As disease severity progresses to moderate and severe NPDR, the number of microaneurysms and haemorrhages increase, and hard exudates, cotton‐wool spots, venous beading, and intraretinal microvascular abnormalities (IRMA) develop, signifying increasing capillary loss, hyperpermeability and non‐perfusion. Severe NPDR is defined by the '4:2:1: rule', which is the presence of retinal haemorrhages in all four quadrants, venous beading in at least two quadrants, or IRMA in at least one quadrant.

Retinal ischaemia (also referred to as retinal capillary non‐perfusion) is considered to be the main catalyst for the occurrence of PDR. PDR is characterised by the development of abnormal new blood vessels (so‐called 'new vessels'), with or without accompanying fibrous tissue (i.e. fibrovascular membranes), at the optic disc (new vessels in the disc (NVD)) or elsewhere in the retina (new vessels elsewhere (NVE)). The ischaemic retina triggers the release of growth factors, including vascular endothelial growth factor (VEGF), which promote the growth of these new vessels in a futile attempt to restore vascular supply to the retina. However, new vessels are fragile and often rupture, leading to haemorrhages inside the eye (so‐called vitreous haemorrhages or pre‐retinal haemorrhages). PDR can progress in severity from mild to high‐risk characteristics (HRC‐PDR). The latter is defined by the presence of NVD of more than one‐fourth to one‐third disc area in size, or NVD or NVD/NVE of any size associated with bleeding, in the form of vitreous or pre‐retinal haemorrhages (Diabetic Retinopathy Study Research Group 1991). In severe cases, PDR can lead to complete visual loss resulting from proliferation of fibrovascular membranes and retinal detachment.

Almost all, if not all, individuals with DM will develop DR if they live for a sufficient period of time. During the first two decades of disease, nearly all people with type 1 diabetes (T1D) and 60% of those with type 2 diabetes (T2D) develop DR (Fong 2003). A pooled analysis to determine the global prevalence of DR found that over one‐third of individuals with DM had DR; of these, approximately 7%, equating to 17 million individuals, will develop PDR (Yau 2012). A more recent pooled analysis estimated the global prevalence of PDR to be 1.4% for the period of 2015 to 2019 (Thomas 2019). However, the authors acknowledge significant heterogeneity in study populations and methodology as limiting factors in accurately deriving the global prevalence of DR and PDR (Thomas 2019).

#### Treatment

The International Diabetes Federation advises regular eye examinations every one to two years for people with diabetes and no retinopathy (IDF 2021). Once DR develops, the frequency of assessments should be increased depending on the severity of the retinopathy and level of control of systemic factors (Fred Hollows Foundation 2015). Currently, treatment options for NPDR are scarce (Royle 2015); treatment is most often only given when PDR or diabetic macular oedema (DMO) have ensued.

The Diabetic Retinopathy Study (DRS) demonstrated that risk of severe visual loss in people with HRC‐PDR was reduced by 50% at two and five years with laser panretinal photocoagulation (PRP) treatment (Diabetic Retinopathy Study Research Group 1987). A Cochrane intervention review also verified that PRP is beneficial in reducing vision loss and progression in PDR (Evans 2014). PRP involves burning the retina, avoiding the macula (the area responsible for the central sight), with spots of laser, leading to regression of new vessels following treatment. The exact mechanism of action of PRP remains unclear, but it is presumably due to the reduced oxygen requirement of the less extensive viable retina post‐treatment, and diminished growth factor production resulting from ablation of the ischaemic retina (Doft 1984). PRP generally preserves rather than improves vision and may be associated with adverse side effects, such as diminished peripheral vision, night vision, or both, and exacerbation of DMO.

The advent of intravitreal anti‐VEGF injections has become a pharmacological alternative to PRP (Cheung 2010). A 2014 Cochrane intervention review determined that evidence from randomised controlled trials (RCTs) for the efficacy and safety of anti‐VEGF drugs in the treatment of PDR was of low quality, but did find a reduction in the risk of intraocular bleeding (Martinez‐Zapata 2014). Recent trials have shown that anti‐VEGFs are non‐inferior to PRP in the treatment of PDR (Gross 2015; Sivaprasad 2017). However, the great majority of participants included in these RCTs did not have HRC‐PDR, where laser PRP has been shown to be most beneficial. Furthermore, anti‐VEGFs appear not to have any beneficial effect on retinal ischaemia, which seems to continue to progress despite this treatment (Chatziralli 2022; Zhu 2021). Recent studies have shown that people with PDR who are treated with anti‐VEGF therapy alone and become temporarily lost to follow‐up are more susceptible to developing irreversible blindness when compared with those treated with laser PRP (Obeid 2018; Wubben 2019). Furthermore, anti‐VEGFs do not appear to be cost‐effective unless they are used to treat people with concomitant DMO and PDR (Hutton 2017). Given that several long‐term studies have verified that the beneficial effects of PRP generally last indefinitely (Chew 2003; Dogru 1999), PRP remains the mainstay therapy for PDR. Even with treatment, however, progression of PDR and the development of further complications may still occur in severe cases.

#### Moment of prognostication

The moment of prognostication is any time after an individual has been diagnosed as having diabetes and DR, and prior to the occurrence of PDR.

#### Clinical context

Although many people develop DR, few will progress to the stage of PDR. However, all individuals with DR require lifelong follow‐up, and diabetic eye screening services and eye health services are currently finding it very challenging to contend with the demand (Foot 2017). A concerning report revealed that lack of capacity within hospital eye services resulted in permanent sight loss in people of all ages, due to delayed appointments, including in people with DR (Foot 2017). The Liverpool Risk Calculation Engine study group determined that implementing individualised screening intervals based on standard clinical data would facilitate more effective management of resources into targeting high‐risk groups (Eleuteri 2017). Thus, identifying prognostic factors signalling risk of visual loss would be extremely beneficial in the enhancement and development of predictive models to optimise resources.

### Description of the prognostic factors

This systematic review focused on identifying prognostic factors for progression from DR to PDR and to HRC‐PDR. We outline some of the risk factors below.

Diabetes duration appears to be a key predictor of the development and progression of DR, independent of glycaemic control (Fong 2003). For example, in individuals with T1D, PDR is not usually observed for the first 10 years of disease, but there is a rapid increase in incidence, to approximately 60%, by 20 years of disease duration (Klein 2008).    

The Diabetes Control and Complications Trial (DCCT) provided evidence that rigorous glycaemic control delays development and progression of DR in T1D (Diabetes Control and Complications Group 1998). Similarly, the UK Prospective Diabetes Study (UKPDS) was pivotal in establishing the beneficial effect of regulating glycaemic levels in people with T2D (Turner 1998). A meta‐analysis of 16 RCTs found that the risk of retinopathy progression was lower after two years of intensive glucose control (Wang 1993). However, it concluded that progression to and within NPDR is clinically different from progression to PDR, but not all studies separate these stages. In those that did, long‐term intensive glucose control significantly reduced retinopathy progression to PDR (odds ratio (OR) 0.44, 95% confidence interval (CI) 0.22 to 0.87; P = 0.018; test for heterogeneity, P = 0.991) (Wang 1993). 

A Cochrane Review assessed the effects of intensive versus conventional glycaemic control on long‐term diabetic complications in people with T1D, and aimed to determine whether near normoglycaemic values are beneficial. The review confirmed that tight blood sugar control significantly reduced the risk of developing retinopathy (23/371 (6.2%) versus 92/397 (23.2%); risk ratio (RR) 0.27, 95% CI 0.18 to 0.42; P < 0.001; 2 studies, 768 participants; high‐quality evidence). However, the beneficial effect of tight blood sugar control seemed to become weaker once retinopathy was present (Fullerton 2014). A recent review, consisting of five RCTs with large sample sizes and long‐term follow‐up, found that in people with worse‐than‐moderate NPDR, intensive glycaemic control may not confer any benefits in terms of progression (Liu 2020).

International evidence‐based clinical practice guidelines recognise the benefit of glycaemic control (Fred Hollows Foundation 2015). However, current management approaches do not fully prevent progression to PDR, and there is no glycaemic threshold below which protection is certain (Diabetes Control and Complications Group 1993). 

Prior to the undertaking of this systematic review, the current evidence on the effect of hypertension on progression to and within PDR seemed unclear. Although the Wisconsin Epidemiological Study of Diabetic Retinopathy determined hypertension to be associated with progression to PDR in people with T1D (Klein 1998), and the UKPDS identified a corresponding relationship in those with T2D (Turner 1998), other studies failed to corroborate these findings (Chew 2014; Harris 2013; Jin 2015). In the Action to Control Cardiovascular Risk in Diabetes (ACCORD) Eye study, intensive blood pressure control did not have a significant effect on retinopathy progression (Chew 2014). A Cochrane Review of 15 RCTs, including participants with T1D and T2D conducted mainly in North America and Europe, determined an association between reduced blood pressure and prevention of DR for up to four to five years (Do 2015). However, the review concluded that the available evidence did not support a benefit of intervention on blood pressure on progression to PDR or moderate/severe visual loss after five years of follow‐up. Similarly, a recent meta‐analysis concluded that intensive blood pressure control reduced relative risk of incidence of DR by 17% in T2D (Zhou 2018a). However, the available data were insufficient to confirm a relative risk reduction for DR progression or incidence of PDR (Zhou 2018a).  

The effect of cholesterol on the progression of DR also remains uncertain. The Collaborative Atorvastatin Diabetes Study found no difference in the progression of DR between participants randomised to receive a daily dose of atorvastatin and those randomised to placebo (Colhoun 2004). Investigation of fibrates in the Fenofibrate Intervention and Event Lowering in Diabetes (FIELD) study found a significant relative reduction in the need for PRP in people with T2D treated with a fibrate; a reduction in DR progression was observed only in those with retinopathy at baseline (Keech 2007). However, it is acknowledged that fenofibrate may benefit the retina independently of its lipid‐lowering effects (reviewed by Stewart and Lois) (Stewart 2018). An ongoing Cochrane Review with a published protocol will evaluate the evidence in this regard (Inoue 2019). A recent systematic review and meta‐analysis of observational studies exploring associations between serum lipids and the occurrence of DR found a slightly higher low‐density lipoprotein (LDL) cholesterol in cases with DR (Zhou 2018b). The review identified that in a large, population‐based, longitudinal, observational study of people with pre‐existing DR at baseline, poor control of total cholesterol was associated with a higher incidence of sight‐threatening retinopathy after adjusting for potential confounders. Poor control of triglycerides was also associated with progression to PDR, and this was greater when all lipid types were abnormal (Srinivasan 2017). There is currently no Cochrane Review evaluating the relationship between cholesterol and DR. Although definitive evidence is lacking regarding the effect of optimal control of blood lipids on reducing the incidence and progression of DR, it is advisable in terms of benefits to overall health.   

Diabetes duration, hyperglycaemia, hypertension, and hyperlipidaemia, whilst likely relevant for determining the risk of DR development (i.e. from no DR to presence of DR), may not fully explain the highly variable progression of NPDR to PDR, as also pointed out in a recent review by Sivaprasad and colleagues (Sivaprasad 2019). Many studies have assessed generalised DR progression using data from screening programmes where the majority of people included had no DR or only mild NPDR. To our knowledge, there are currently no systematic reviews on prognostic factors for the development of PDR and its progression.  

This review aimed to identify factors conferring increased risk of PDR and HRC‐PDR in people with diabetes once retinopathy is present.

### Health outcomes

This review considered the prognostic factors associated with the development of PDR and progression from less than HRC‐PDR to HRC‐PDR. Thus, we investigated the health outcomes PDR and HRC‐PDR.

As stated above, PDR is diagnosed by the presence of: NVD, defined as new vessels on or within one disc diameter of the disc; or NVE, defined as new vessels at any other locations in the retina. HRC‐PDR is defined according to the ETDRS as NVD of more than one‐fourth to one‐third disc area, or NVD or NVE of any size if associated with the presence of vitreous haemorrhage or pre‐retinal haemorrhage.

Alarmingly, many people with diabetes can progress to the sight‐threatening stage of PDR without developing any obvious prior warning symptoms. The DRS found that approximately 50% of people with PDR who do not receive timely treatment will become legally blind within five years (Diabetic Retinopathy Study Research Group 1981a). The ETDRS was important in establishing that PRP treatment can be deferred in people with NPDR or PDR until high‐risk characteristics develop (Diabetic Retinopathy Study Research Group 1991). The study also identified that only 50% of eyes assigned to deferral of treatment (until HRC‐PDR ensued) progressed to HRC‐PDR after seven years of follow‐up (Diabetic Retinopathy Study Research Group 1991).

A large cohort study ‐ of 7.7 million people who contributed data to the Clinical Practice Research Datalink ‐ evaluated population trends in the 10‐year incidence and prevalence of DR in the UK from 2004 to 2014 (Mathur 2017). The study considered trends by diabetes type, age, sex, ethnicity, deprivation, region, and calendar year (Mathur 2017). It found that the age‐standardised prevalence of DR decreased over time from 2.6% to 2.2%, whilst the age‐standardised prevalence of severe DR remained stable at 0.1%. The incidence also remained stable at one event per 10,000 person‐years (Mathur 2017). This suggests that despite improved medical management of DM, the threat of PDR and its complications remain a significant problem.

The time horizon for the evaluation of health outcomes in this review was three years (± two years), eight years (± two years), or lifelong, if available.

### Why it is important to do this review

We undertook this review to gather evidence on prognostic factors for the development and progression of PDR. This information is essential for ophthalmologists and other healthcare professionals for the counselling and management of people with diabetes and thus for people with diabetes and their families. Our findings will help clinicians to provide advice to their patients regarding modifiable risk factors, to determine in a more personalised manner the interval required for the purpose of monitoring their disease, and to consider early intervention in high‐risk groups. Due to the increasing prevalence of diabetes and the limited resources of healthcare systems, tailoring health care in an individualised manner seems essential, avoiding the need to review patients in low‐risk groups too often and guaranteeing prompt and close evaluation and treatment, if required, of those who are at high risk.   

This prognosis review may help to identify targets for new interventions that aim to modify the course of the disease. Furthermore, the findings may guide the design and analysis of future interventional clinical trials, and highlight areas where further research is required.

To our knowledge, there are currently no systematic reviews on prognostic factors specifically for the development of PDR and its progression to high‐risk PDR. A systematic review on prognostic prediction models for DR progression was published recently (Haider 2019). This review aimed to summarise the performance of existing models in predicting progression of retinopathy and the models' applicability for higher‐risk DR patients under hospital care to predict the need for treatment or loss of vision. Based on their findings, the authors identified the need for an accurate model that can determine patients’ individual risk of progression to a treatment stage or loss of vision. They determined that this knowledge will allow for a more appropriate use of resources and further optimisation of services, especially for individuals with a higher risk of progression (Haider 2019). This current Cochrane Review provides evidence‐based information on risk factors for the development and progression of PDR that can be used for the development of future prognostic models.

## Objectives

### Primary objectives

To assess prognostic factors for predicting the occurrence of PDR in individuals with diabetic retinopathy.

#### Table 1. PICOTS of the primary objective

**Table:** 

**Population**	Male and female adults ≥ 18 years of age of any ethnicity with DM and DR (NPDR), diagnosed as per standard clinical protocol
**Index prognostic factors**	Specific prognostic factors of interest included: routinely collected patient demographics and information, such as age, gender, ethnicity, socioeconomic status, and smoking habits; frequently obtained standard clinical data, such as comorbidities (e.g. presence/absence of cardiovascular disease; cerebrovascular disease; nephropathy, and specifically, chronic kidney failure (defined as estimated glomerular filtration rate (GFR) of < 60 mL/min/1.73 m^2^); peripheral neuropathy); body mass index (BMI); neck/waist circumference; glycated haemoglobin; blood pressure; low‐density lipoprotein; high‐density lipoprotein; and triglycerides; and functional and structural retinal biomarkers in the prognostic context of the development and progression of PDR. We considered prognostic factors in the absence of treatment for DR. We expected that prognostic factors would generally have been measured at the time participants entered the studies, and indeed after diagnosis of DR. If measures of prognostic factors were available at other time points, and these coincided in more than one included study, we planned to consider investigating them at these other time points. We excluded studies evaluating risk factors that ‐ to be measured ‐ require invasive procedures (e.g. aqueous or vitreous samples to measure growth factors in these fluids) not performed in routine clinical practice.
**Comparator**	Not applicable
**Outcomes**	Progression from DR (NPDR) to any stage of PDR. We considered participants who received laser PRP for the treatment of PDR to have progressed to the outcome of PDR.
**Timing**	3 years (± 2 years), 8 years (± 2 years), or lifelong, if available. PDR can occur very rapidly ‐ in days ‐ or take months or years to develop.
**Setting**	Any clinical setting. No geographical limitations

### Secondary objectives

To assess prognostic risk factors for predicting the progression of PDR from less than HRC‐PDR to HRC‐PDR.

#### Table 2. PICOTS of the secondary objective

**Table:** 

**Population**	Male and female adults ≥ 18 years of age of any ethnicity with less than HRC‐PDR, diagnosed as per standard clinical protocol
**Index prognostic factors**	We anticipated that less information would be available regarding prognostic factors associated with progression from PDR to HRC‐PDR. Prognostic factors of interest included: routinely collected patient demographics and information, such as age, gender, ethnicity, socioeconomic status, and smoking habits; frequently obtained standard clinical data, such as comorbidities (presence/absence of cardiovascular disease; cerebrovascular disease; nephropathy, and specifically, chronic kidney failure (defined as estimated GFR of < 60 mL/min/1.73 m^2^); peripheral neuropathy); BMI, neck/waist circumference; glycated haemoglobin; blood pressure; low‐density lipoprotein; high‐density lipoprotein; and triglycerides; and functional and structural retinal biomarkers in the prognostic context of the development and progression of HRC‐PDR*.* The scope of this review did not extend to the evaluation of the effect of treatment on progression to HRC‐PDR. Given this, we considered prognostic factors in the absence of previous treatment for PDR. Prognostic factors were generally measured at the time participants entered the studies, and indeed, after the diagnosis of less than HRC‐PDR. However, where measures of prognostic factors were available at other time points, and these coincided in more than one included study, we investigated them at these other time points. We did not consider prognostic factors that ‐ to be measured ‐ require invasive procedures (e.g. aqueous or vitreous samples to measure growth factors in these fluids) not performed in routine clinical practice.
**Comparator**	Not applicable
**Outcomes**	Progression from PDR to HRC‐PDR.
**Timing**	3 years (± 2 years), 8 years (± 2 years), or lifelong, if available. HRC‐PDR can occur very rapidly ‐ in days ‐ or take months or years to develop.
**Setting**	Any clinical setting. No geographical limitations

## Methods

### Criteria for considering studies for this review

#### Types of studies

##### Inclusion criteria

Eligible study designs included prospective or retrospective cohort and case‐control longitudinal studies including participants who have not had previous treatment for DR. Although we initially planned to include randomised controlled trials (RCTs) evaluating therapeutic interventions to prevent progression of DR where there was a control, untreated arm, ultimately we decided not to include these (see Differences between protocol and review). We also included studies based on longitudinal registry data. It was a mandatory requirement for inclusion in the review that studies had to evaluate prognostic factors specifically for the development and progression of PDR, as opposed to generalised progression of DR.

Studies investigating general microvascular complications of diabetes but including a subset of data related to factors involved in the development of PDR were eligible for inclusion if specific information on this group (PDR) was given.

##### Exclusion criteria

We excluded case reports, as they would have introduced selection bias, and editorials and letters to editors not containing primary data. We did not include cross‐sectional studies, as this type of study design is less appropriate for the evaluation of prognostic factors for the development or progression of disease.

#### Targeted population

The target population consisted of adults (≥ 18 years of age) of any gender with NPDR or PDR with less than HRC‐PDR, diagnosed as per standard clinical practice. Studies including participants of all ethnicities, geographical locations, and socioeconomic status were eligible for inclusion. Any appropriate studies including a subset of relevant participants were considered as potentially eligible if data from this subset were given separately.

#### Types of prognostic factors

This review considered and included prognostic factor studies only. Specific prognostic factors of interest included, but were not restricted to:

- routinely collected patient demographics and information, such as age, gender, ethnicity, socioeconomic status, and smoking habits;
- frequently obtained standard clinical data, such as comorbidities (presence/absence of cardiovascular disease, cerebrovascular disease, nephropathy and specifically chronic kidney failure (defined as estimated GFR of < 60 mL/min/1.73 m^2^), peripheral neuropathy and specifically foot ulcers, amputation), BMI, neck/waist circumference, glycated haemoglobin, blood pressure, low‐density lipoprotein, high‐density lipoprotein, triglycerides; and
- functional and structural retinal biomarkers in the prognostic context of the development and progression of PDR. 

We excluded studies evaluating prognostic factors involving invasive procedures that cannot be practically undertaken in a clinical setting (such as aqueous/vitreous sampling) and are thus unlikely to be translatable to routine clinical practice.

We expected that prognostic factors would generally have been measured at the time participants entered the studies, and indeed after diagnosis of DR or PDR. If measures of prognostic factors were available at other time points, and these coincided in more than one study, we planned to consider investigating them at these other time points.

#### Types of outcomes to be predicted

##### Development of PDR

The development of PDR was determined by the presence of NVD or NVE, as diagnosed based on fundus examination, fundus photography, or fundus fluorescein angiography. We considered participants requiring laser treatment for PDR specifically to have progressed to the outcome of PDR.

##### Development of HRC‐PDR

Progression from less than HRC‐PDR to HRC‐PDR. HRC‐PDR was defined according to the ETDRS as: i) NVD 0.5 disc area plus vitreous haemorrhage or pre‐retinal haemorrhage; ii) vitreous haemorrhage or pre‐retinal haemorrhage obscuring more than one disc area (Diabetic Retinopathy Study Research Group 1991). These features could have been determined by clinical examination or by the grading of ophthalmic images, both fundus photography and fundus fluorescein angiograms. Participants requiring laser treatment for HRC‐PDR specifically were considered as having progressed to the outcome of HRC‐PDR. 

The time horizon for the evaluation of health outcomes in this review was three years (± two years), eight years (± two years), or lifelong, if available. If not, we accepted and presented other time points.

### Search methods for identification of studies

#### Electronic searches

A medical librarian specialist from Queen’s University Belfast, and the Cochrane Eyes and Vision Information Specialist searched the following electronic databases. There were no restrictions on language or year of publication. The date of the search was 27 May 2022. The search was developed around the following components: “prognostic factors”, “proliferative diabetic retinopathy”, and “development and progression”. 

- Cochrane Central Register of Controlled Trials (CENTRAL; 2022, Issue 5) (which contains the Cochrane Eyes and Vision Trials Register) in the Cochrane Library (searched 27 May 2022) (Appendix 1).
- MEDLINE Ovid (1946 to 27 May 2022) (Appendix 2).
- Embase Ovid (1980 to 27 May 2022) (Appendix 3).

#### Searching other sources 

We supplemented the above searches by screening reference lists of all eligible articles. We did not include grey literature sources in the review, as we did not expect these to be sufficiently informative to justify the extra resources required to conduct these searches.

### Data collection

#### Selection of studies

Two review authors (amongst JP, NL, JE, RH, JL), independently and masked to each other’s initial decisions, reviewed titles and abstracts of studies identified by the electronic searches and classified them as potentially eligible or ineligible. We used an online review management software for this purpose (Covidence). Discrepancies were resolved by discussion. We obtained full‐text articles of potentially eligible studies. Two review authors (amongst JP, NL, JE, RH, JL) independently classified them as included or excluded. Discrepancies were resolved by discussion. We recorded the study selection process in a PRISMA flow diagram, specifying reasons for exclusion of studies excluded after full‐text review. One review authors (JP) scrutinised reference lists of included studies; two independent reviewers (amongst JP, NL, JE, RH, JL) then classified studies as potentially eligible or ineligible. Two independent review authors ( amongst JP, NL, JE, RH, JL) then retrieved full‐text articles of potentially eligible studies for review and classified them as eligible or ineligible. As above, discrepancies were resolved by discussion.

#### Data extraction and management

To account for heterogeneity amongst studies, data extraction involved two stages. The first stage consisted of a mapping exercise to categorise eligible studies according to their design, prognostic factors evaluated, time points of prognostic factor measurements and outcomes, and type of analysis/effect estimates. One review author (JP) undertook this stage. Information was then entered into a pilot‐tested spreadsheet specifically designed for this purpose and reviewed by the review team. 

In the second stage, data were extracted, firstly during a pilot stage, and then in full, for all eligible studies. Two review authors (amongst JP, JC, EL, NL) independently undertook data extraction. Disagreements were resolved by discussion or with the involvement of a third review author. We used the Checklist for Critical Appraisal and Data Extraction for Systematic Reviews of Prediction Modelling Studies (CHARMS‐PF) to guide data extraction (Appendix 4).

We extracted and entered the following data, if available, according to the following categories.

- Study
  - Title
  - Authors’ contact details
  - Sources of funding
  - Dates
- Study design
  - Prospective or retrospective cohort or case‐control studies and longitudinal registry data
- Participants
  - Eligibility criteria and recruitment method
  - Participant description
  - Details of treatments received, if relevant
- Outcomes to be predicted
  - Definition and method of measurement of outcomes
  - Types of outcomes: 1) developing PDR; 2) progressing from less than HRC‐PDR to HRC‐PDR
  - Time of outcome occurrence
- Prognostic factors
  - Number and type of prognostic factors
  - Definition and method for measurement
  - Timing of prognostic factor measurement
- Sample size
  - Sample size calculation
  - Number of participants and number of outcomes
  - Outcomes per variable
- Missing data
- Analysis
  - Modelling method
- Results
  - Unadjusted and adjusted prognostic effect estimates (e.g. risk ratio, odds ratio, hazard ratio, or mean difference) for each prognostic factor of interest and corresponding measure of uncertainty (e.g. standard errors, variances, or confidence intervals)
  - For each extracted adjusted prognostic effect estimate of interest, the set of adjusted factors

#### Assessment of risk of bias in included studies

We used the Quality in Prognosis Studies (QUIPS) tool to assess risk of bias of the included studies (Appendix 5) (Hayden 2013). We considered six risk of bias domains: study participation, study attrition, prognostic factor measurement, outcome measurement, adjustment for other prognostic factors, and statistical analysis and reporting. 

The study participation domain consisted of six items: adequate participation in study by eligible individuals (i.e. sampling frame and recruitment adequately described, including methods to identify the sample); description of target population (i.e. source population for cohort with DR is clearly described); description of baseline study sample (i.e. number of people with DR at baseline is given); adequate description of recruitment process (i.e. way of establishing the sample population, selection criteria, and key characteristics of the population clearly described); adequate description of period and place of recruitment (time period and place of recruitment for both baseline and follow‐up examinations are clearly described); and adequate description of inclusion/exclusion criteria.

The study attrition domain consisted of five items: adequate response rate for study participants of at least 80%; description of process for collecting information on participants who dropped out (i.e. attempts to collect information on participants who dropped out are described); reasons for loss to follow‐up provided; adequate description of participants lost to follow‐up; and no important differences between participants who completed the study and those who dropped out. 

The prognostic factor domain consisted of six items: clear definition of prognostic factor provided; method of prognostic factor measurement is adequately valid and reliable; continuous variables are reported (i.e. standard categories for prognostic factors/cut‐offs); method and setting of measurement of prognostic factor is identical for all participants; adequate proportion of study sample has complete data for prognostic factor; and appropriate methods of imputation used for missing prognostic factor data. 

The outcome measurement domain consisted of three items: clear definition of outcome provided; method of outcome measurement is adequately valid and reliable (measurement of PDR/HRC as part of a diagnostic assessment); and method and setting of outcome measurement is identical for all participants. 

The adjustment for other prognostic factors domain consisted of seven domains: all other important prognostic factors measured (i.e. HbA1c and duration of DM as a minimum); clear definitions of important prognostic factors measured provided; measurement of all important prognostic factors adequately valid and reliable; measurement and setting of prognostic factor measurement identical for all participants; appropriate methods are used to deal with missing values of prognostic factors (i.e. strategy to impute missing confounder data is described); important prognostic factors accounted for in study design; and important prognostic factors accounted for in analysis (i.e. important confounders are accounted for in multivariable logistic regression and Cox proportional hazards models). 

The statistical analysis and reporting domain consisted of four items: sufficient presentation of data to assess adequacy of analytic strategy (i.e. mean or median values, including confidence intervals or standard errors or standard deviations provided); strategy for model‐building appropriate and based on a conceptual framework or model; selected statistical model adequate for design of study (mainly incidence rates, uni‐ and multivariate logistic regression, Cox proportional hazard models); and no selective reporting of results.

Two review authors (amongst JP, JC, EL, NL) independently assessed risk of bias. We assessed each risk of bias domain as low, moderate, or high risk, and detailed the reasoning for such assessments (see Appendix 6 for signalling questions).

#### Measures of association or predictive performance measures extracted 

For each factor of interest, we extracted estimates of prognostic effect, such as hazard ratios (HR), risk ratios (RR), odds ratios (OR), or mean differences (MD) with a measure of their uncertainty (standard errors (SE), variances, or confidence intervals (CIs)). We collected adjusted prognostic effect estimates preferentially and documented the set of adjustment factors used.

#### Dealing with missing data

We contacted study authors when we required further information or clarification. When time‐to‐event analyses were performed, and adjusted hazard ratio estimates and their uncertainty were unavailable, we planned to derive unadjusted estimates and their standard errors, following guidance described by Tierney and colleagues (Tierney 2007), if the summary statistics reported permitted it.

#### Investigation of sources of heterogeneity between studies

Between‐study heterogeneity related to two key areas:

- clinical heterogeneity, including the effect of different comorbidities, medications, and interventions in study cohorts, and differences in how outcomes were measured, such as diagnoses of PDR (clinical examination versus supported by imaging/imaging technologies used) and how progression was defined;
- methodological heterogeneity generated from different study designs, and how robustly studies were conducted with regard to risk of bias and approach to analysis. 

We explored the effects of these aspects of heterogeneity on the meta‐analyses we undertook.

Since the I^2^ statistic can be problematic in certain situations (Rücker 2008), we planned to quantify heterogeneity using Tau^2^. Where there was an appropriate number of studies included in a meta‐analysis, we also planned to present 95% prediction intervals.

#### Assessment of reporting deficiencies 

We planned to assess small‐study effects using contour‐enhanced funnel plots when 10 or more studies were included in a meta‐analysis. We anticipated variation in effect measures, length of follow‐up, and other characteristics, and therefore expected to include few studies in each meta‐analysis. Consequently, we did not plan to perform funnel plot asymmetry tests given the low power of such tests when studies are few (Debray 2018).

### Data synthesis

#### Data synthesis and meta‐analysis approaches

We conducted meta‐analysis (i.e. report a weighted average of the individual study measures of association) in clinically relevant groups using a random‐effects approach. We stratified by different time points of outcomes and meta‐analysed HR, OR, and RR separately for each prognostic factor and outcome available for meta‐analysis. Similarly, we reported unadjusted and adjusted associations separately. Our primary analyses focused on adjusted estimates, but we could only do two of this type of meta‐analysis due to insufficient data available. We present most data below in a narrative or tabulated summary because we did not identify enough studies of sufficient homogeneity to permit meta‐analysis. We used 95% confidence intervals throughout. 

#### Sensitivity analysis 

We planned to perform sensitivity analyses to explore the impact of the following factors (when applicable) on effect sizes by excluding:

- studies at high risk of bias in one or more domains;
- retrospective studies.

However, due to the very limited data available, we were unable to perform these analyses.

#### Conclusions and summary of findings table

We prepared a summary of findings table assessing the certainty of the evidence using GRADE modified for prognostic factor studies (Foroutan 2020; Higgins 2022). In this table, we included all prognostic factors investigated in eligible studies for their potential role in the development of PDR using adjusted analysis in multivariable regression models. The certainty of evidence was based on grading the following domains: risk of bias; inconsistency; imprecision; and indirectness. We rated evidence down for risk of bias if more than 80% of studies included in the multivariable regression analyses had unclear or high risk of bias. We rated evidence down for inconsistency when the direction of effect estimates differed amongst studies, and for imprecision when confidence intervals were wide. If populations and outcomes evaluated did not correspond to the populations and outcomes of interest in the review, we rated the certainty of the evidence down for indirectness.

Two independent review authors (NL, JP) conducted the GRADE assessments, and resolved any discrepancies through discussion. We used the summary of findings table to clearly identify factors that influenced the development of PDR and our confidence in the estimates of effects observed. We planned to also include HRC‐PDR in this table, but there were no data available.

## Results

### Results of the search

The electronic searches yielded a total of 8007 records. After removing 1776 duplicates, a total of 6231 records remained. We also identified an additional 160 records by screening the reference lists of eligible studies, and undertaking additional searches for relevant studies to supplement the Background and Discussion sections of the manuscript. In total, we screened 6391 records at the title and abstract screening stage, and determined that 6087 records were irrelevant to the review. We classified the remaining 304 records as 'potentially eligible', and retrieved full‐text articles to screen them for eligibility. We contacted the corresponding authors of three studies to request additional information necessary to determine eligibility for inclusion in our review (Leese 2004; Mathur 2016; Takaike 2018). We did not establish contact with one author (Takaike 2018); the other two authors provided further information, which allowed us to confirm that these studies were not eligible for inclusion in our review. Thus, we identified a total of 87 reports of 59 studies as eligible for inclusion in this review (see Figure 1 for study flow diagram).

**1:**
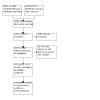
PRISMA study flow diagram

#### Included studies

Of the included studies, several articles referred to the same original study cohorts. Thus, the WESDR study reported its findings on various prognostic factors associated with PDR in T1D and T2D at different time points, ranging from four to 25 years in 23 separate publications. The population was also divided into a “younger‐onset” group (diabetes diagnosis at < 30 years) and “older‐onset” group (diabetes diagnosis at ≥ 30 years), and further subdivided into participants “taking insulin” or “not taking insulin” (WESDR). Similarly, the studies by Grauslund 2009, Hsieh 2018, Lloyd 1995, Mathiesen 1990, Nielsen 1984, and Roy 2006 reported findings on different prognostic factors and time points in their cohorts in separate reports.

Of the 59 unique studies included in this review, 25 were conducted in Europe (Bojestig 1998; Burditt 1968; Grauslund 2009; Gurreri 2019; Hovind 2003; Janghorbani 2000; Jones 2012; Keen 2001; Kofoed‐Enevoldsen 1987; Kullberg 1993; Lee 2017; Lestradet 1981; Mathiesen 1990; McCance 1989; Nielsen 1984; Nordwall 2015; Pirart 1977; Porta 2001; Simonsen 1980; Skrivarhaug 2006; Styles 2000; Teuscher 1988; Vesteinsdottir 2010; Voigt 2018; Zavrelova 2011), 18 in North America (Arfken 1998; Ballard 1986; Dwyer 1985; Gange 2021; Hardin 1956; Harris 2013; Klein 1984; Lee 1992; Lloyd 1995; Nelson 1989; Pambianco 2006; Rodriguez‐Villalobos 2005; Roy 2006; Rudnisky 2017; Silva 2015; Valone 1981; Varma 2010; WESDR), 13 in Asia (Chawla 2021; Chen 1995; Cho 2019; Gui 2013; Hsieh 2018; Jeng 2016; Kalter‐Leibovici 1991; Kim 1998; Kim 2014; Lee 2021; Miki 1969; Okudaira 2000; Yokoyama 1994), and one each in Africa (Burgess 2015), South America (Rodriguez‐Villalobos 2005), and Australia (McCarty 2003).

Fifty‐seven studies were prospective cohort (n = 35) (Bojestig 1998; Burgess 2015; Chen 1995; Grauslund 2009; Hardin 1956; Hovind 2003; Hsieh 2018; Kalter‐Leibovici 1991; Keen 2001; Kim 1998; Kullberg 1993; Lee 1992; Lestradet 1981; Lloyd 1995; Mathiesen 1990; McCance 1989; McCarty 2003; Miki 1969; Nelson 1989; Nielsen 1984; Nordwall 2015; Okudaira 2000; Pambianco 2006; Pirart 1977; Porta 2001; Rodriguez‐Villalobos 2005; Roy 2006; Silva 2015; Simonsen 1980; Skrivarhaug 2006; Teuscher 1988; Valone 1981; Varma 2010) or retrospective cohort (n = 22) (Arfken 1998; Ballard 1986; Burditt 1968; Chawla 2021; Cho 2019; Gui 2013; Gurreri 2019; Janghorbani 2000; Kim 2014; Lee 2021; Rudnisky 2017; Verdaguer 2009; Vesteinsdottir 2010; Voigt 2018; WESDR; Yokoyama 1994; Zavrelova 2011) studies, with six of these based on data from electronic registers only (Dwyer 1985; Gange 2021; Harris 2013; Jeng 2016; Jones 2012; Lee 2017). The two remaining studies were retrospective case‐control studies (Kofoed‐Enevoldsen 1987; Styles 2000).

Twenty‐three studies evaluated participants with T1D (Arfken 1998; Bojestig 1998; Chawla 2021; Grauslund 2009; Hardin 1956; Hovind 2003; Kalter‐Leibovici 1991; Klein 1984; Kofoed‐Enevoldsen 1987; Kullberg 1993; Lestradet 1981; Lloyd 1995; Mathiesen 1990; McCance 1989; Nordwall 2015; Pambianco 2006; Porta 2001; Roy 2006; Simonsen 1980; Skrivarhaug 2006; Styles 2000; Verdaguer 2009; Yokoyama 1994), 19 with T2D (Ballard 1986; Chen 1995; Cho 2019; Gange 2021; Gui 2013; Gurreri 2019; Hsieh 2018; Jeng 2016; Kim 1998; Kim 2014; Lee 1992; Lee 2021; Nelson 1989; Okudaira 2000; Rodriguez‐Villalobos 2005; Rudnisky 2017; Valone 1981; Voigt 2018; Zavrelova 2011), and 17 included mixed populations (T1D and T2D) (Burditt 1968; Burgess 2015; Dwyer 1985; Harris 2013; Janghorbani 2000; Jones 2012; Keen 2001; Lee 2017; McCarty 2003; Miki 1969; Nielsen 1984; Pirart 1977; Silva 2015; Teuscher 1988; Varma 2010; Vesteinsdottir 2010; WESDR). Of the latter, three included participants with T1D and T2D but reported outcomes for the subgroups separately (Janghorbani 2000; Nielsen 1984; WESDR). In one study, the type of diabetes (T1 or T2) was not specified (Jeng 2016). 

Studies on T1D included from 39 to 3250 participants at baseline, followed up for one to 45 years. Studies on T2D included from 100 to 71,817 participants at baseline, followed up for one to 20 years. The studies on mixed populations of T1D and T2D ranged from 76 to 32,553 participants at baseline, followed up for four to 25 years.

We attempted to contact corresponding authors of six studies included in the review to request clarification on methodology (Chawla 2021; Gange 2021; Harris 2013; Jeng 2016) or results (Lee 2017; Lee 2021). We did not establish contact with three of these authors (Chawla 2021; Jeng 2016; Lee 2021). The others provided further information which we used in this review.

We present detailed descriptions of the included studies in supplementary files, which can be viewed here: osf.io/sjfy5/?view_only=23c87cd105bb49639d88b90cee1e68d1.

#### Excluded studies

We excluded 217 reports of 140 studies. See Characteristics of excluded studies for details.

### Risk of bias in included studies

We provide a summary of the risk of bias results for each of the domains (study participation, study attrition, prognostic factor measurement, outcome measurement, adjustment for other prognostic factors, and statistical analysis and reporting) in Figure 2 and Figure 3.

**2:**
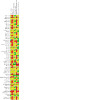
Risk of bias summary: review authors' judgements about each methodological quality item for each included study

**3:**
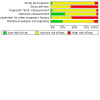
Risk of bias graph: review authors' judgements about each risk of bias item presented as percentages across all included studies

#### Study participation

Only two studies reported adequately on this domain (Janghorbani 2000; WESDR). In the remaining studies, reporting was unclear in 53 (Arfken 1998; Ballard 1986; Bojestig 1998; Burditt 1968; Burgess 2015; Chawla 2021; Chen 1995; Cho 2019; Dwyer 1985; Gange 2021; Grauslund 2009; Gui 2013; Gurreri 2019; Hardin 1956; Harris 2013; Hovind 2003; Hsieh 2018; Jones 2012; Kalter‐Leibovici 1991; Keen 2001; Kim 1998; Kim 2014; Klein 1984; Kofoed‐Enevoldsen 1987; Kullberg 1993; Lee 1992; Lee 2017; Lee 2021; Lestradet 1981; Lloyd 1995; Mathiesen 1990; McCance 1989; Miki 1969; Nelson 1989; Nielsen 1984; Nordwall 2015; Okudaira 2000; Pambianco 2006; Pirart 1977; Porta 2001; Roy 2006; Rudnisky 2017; Silva 2015; Simonsen 1980; Skrivarhaug 2006; Styles 2000; Teuscher 1988; Valone 1981; Varma 2010; Vesteinsdottir 2010; Voigt 2018; Yokoyama 1994; Zavrelova 2011), and at high risk of bias in four (Jeng 2016; McCarty 2003; Rodriguez‐Villalobos 2005; Verdaguer 2009). This was due to inadequate participation in the study by eligible individuals (n = 47/59, 80%), description of the target population (n = 45/59, 76%), description of baseline study sample (n = 34/59, 57%), description of the recruitment process (n = 44/59, 75%), the period and place of recruitment (n = 25/59, 42%), and description of the inclusion/exclusion criteria (n = 34/59, 58%).

#### Study attrition

The risk of bias for study attrition was unclear in 25 studies (Bojestig 1998; Chen 1995; Grauslund 2009; Gui 2013; Hardin 1956; Hovind 2003; Hsieh 2018; Kim 1998; Kim 2014; Klein 1984; Lee 1992; Lloyd 1995; McCarty 2003; Nielsen 1984; Nordwall 2015; Okudaira 2000; Pambianco 2006; Porta 2001; Roy 2006; Rudnisky 2017; Skrivarhaug 2006; Teuscher 1988; Valone 1981; Varma 2010; WESDR) and high in 34 (Arfken 1998; Ballard 1986; Burditt 1968; Burgess 2015; Chawla 2021; Cho 2019; Dwyer 1985; Gange 2021; Gurreri 2019; Harris 2013; Janghorbani 2000; Jeng 2016; Jones 2012; Kalter‐Leibovici 1991; Keen 2001; Kofoed‐Enevoldsen 1987; Kullberg 1993; Lee 2017; Lee 2021; Lestradet 1981; Mathiesen 1990; McCance 1989; Miki 1969; Nelson 1989; Pirart 1977; Rodriguez‐Villalobos 2005; Silva 2015; Simonsen 1980; Styles 2000; Verdaguer 2009; Vesteinsdottir 2010; Voigt 2018; Yokoyama 1994; Zavrelova 2011). This was due to inadequate response rate for study participants (n = 48/59, 81%), inadequate description of the process for collecting information on participants who dropped out (n = 54/59, 92%), reasons for loss to follow‐up not being provided (n = 37/59, 63%), inadequate description of participants lost to follow‐up (46/59, 78%), and important differences between participants who completed the study and those who did not (n = 55/59, 93%).

#### Prognostic factor measurement

Only two studies were at low risk of bias for this domain (McCarty 2003; Porta 2001). In the remaining studies, reporting was unclear in 55 (Arfken 1998; Ballard 1986; Bojestig 1998; Burditt 1968; Burgess 2015; Chawla 2021; Chen 1995; Cho 2019; Dwyer 1985; Gange 2021; Grauslund 2009; Gui 2013; Gurreri 2019; Hardin 1956; Harris 2013; Hovind 2003; Hsieh 2018; Janghorbani 2000; Jeng 2016; Jones 2012; Kalter‐Leibovici 1991; Keen 2001; Kim 1998; Kim 2014; Klein 1984; Kofoed‐Enevoldsen 1987; Kullberg 1993; Lee 1992; Lee 2017; Lee 2021; Lestradet 1981; Lloyd 1995; Mathiesen 1990; McCance 1989; Miki 1969; Nelson 1989; Nielsen 1984; Nordwall 2015; Okudaira 2000; Pambianco 2006; Pirart 1977; Roy 2006; Rudnisky 2017; Silva 2015; Simonsen 1980; Skrivarhaug 2006; Styles 2000; Teuscher 1988; Valone 1981; Varma 2010; Vesteinsdottir 2010; Voigt 2018; WESDR; Yokoyama 1994; Zavrelova 2011), and at high risk of bias in two (Rodriguez‐Villalobos 2005; Verdaguer 2009). This was due to unclear definition of prognostic factor (n = 13/59, 22%), inadequate method of prognostic factor measurement (n = 25/59, 40%), inadequate reporting of continuous variables (n = 41/59, 69%), differences in how prognostic factors were measured (n = 33/59, 56%), inadequate proportion of study sample having complete data for prognostic factor (n = 53/59, 90%), and inappropriate methods of imputation for missing prognostic factor data (n = 57/59, 97%).

#### Outcome measurement

This domain had the highest number of studies at low risk of bias (18) (Arfken 1998; Burgess 2015; Chen 1995; Grauslund 2009; Gurreri 2019; Hsieh 2018; Klein 1984; Kullberg 1993; Lee 1992; Lloyd 1995; McCance 1989; McCarty 2003; Porta 2001; Roy 2006; Silva 2015; Teuscher 1988; Varma 2010; WESDR). In the remaining studies, reporting was unclear in 39 (Ballard 1986; Bojestig 1998; Burditt 1968; Chawla 2021; Cho 2019; Dwyer 1985; Gange 2021; Gui 2013; Hardin 1956; Harris 2013; Hovind 2003; Janghorbani 2000; Jones 2012; Kalter‐Leibovici 1991; Keen 2001; Kim 1998; Kim 2014; Klein 1984; Kofoed‐Enevoldsen 1987; Lee 2017; Lee 2021; Lestradet 1981; Miki 1969; Nelson 1989; Nielsen 1984; Nordwall 2015; Okudaira 2000; Pambianco 2006; Pirart 1977; Rodriguez‐Villalobos 2005; Rudnisky 2017; Simonsen 1980; Skrivarhaug 2006; Styles 2000; Valone 1981; Verdaguer 2009; Vesteinsdottir 2010; Voigt 2018; Yokoyama 1994; Zavrelova 2011), and at high risk of bias in two (Jeng 2016; Mathiesen 1990). This was due to unclear definition of outcome (n = 44/59, 25%), unreliable method of outcome measurement (n = 28/59, 47%), and differences in method and setting of outcome measure (n = 44/59, 58%).

#### Adjustment for other prognostic factors

We assessed only one study as being at low risk of bias in this domain (Porta 2001). In the remaining studies, reporting was unclear in 25 studies (Arfken 1998; Bojestig 1998; Cho 2019; Gange 2021; Grauslund 2009; Gui 2013; Harris 2013; Hsieh 2018; Janghorbani 2000; Kalter‐Leibovici 1991; Kim 1998; Kim 2014; Lee 1992; Lloyd 1995; Mathiesen 1990; Nelson 1989; Nordwall 2015; Okudaira 2000; Roy 2006; Silva 2015; Skrivarhaug 2006; Styles 2000; Voigt 2018; WESDR), and at high risk of bias in 33 (Ballard 1986; Burditt 1968; Burgess 2015; Chawla 2021; Chen 1995; Dwyer 1985; Gurreri 2019; Hardin 1956; Hovind 2003; Jeng 2016; Jones 2012; Keen 2001; Klein 1984; Kofoed‐Enevoldsen 1987; Lee 2017; Lee 2021; Lestradet 1981; McCance 1989; McCarty 2003; Miki 1969; Nielsen 1984; Pambianco 2006; Pirart 1977; Rodriguez‐Villalobos 2005; Rudnisky 2017; Simonsen 1980; Teuscher 1988; Valone 1981; Varma 2010; Verdaguer 2009; Vesteinsdottir 2010; Yokoyama 1994; Zavrelova 2011). In 63% of studies, HbA1c and duration of DM were not controlled for when assessing the effect of other prognostic factors.

#### Statistical analysis and reporting

We determined that fifteen studies were at low risk of bias (25%) in the statistical analysis and reporting domain (Cho 2019; Gui 2013; Hsieh 2018; Janghorbani 2000; Jeng 2016; Kalter‐Leibovici 1991; Kim 1998; Kim 2014; Lee 2017; Lloyd 1995; Okudaira 2000; Porta 2001; Roy 2006; Silva 2015; WESDR). In the remaining studies, reporting of risk of bias was unclear in 39 studies (Arfken 1998; Ballard 1986; Bojestig 1998; Burditt 1968; Burgess 2015; Chawla 2021; Chen 1995; Dwyer 1985; Gange 2021; Grauslund 2009; Gurreri 2019; Hardin 1956; Harris 2013; Hovind 2003; Jones 2012; Keen 2001; Klein 1984; Kofoed‐Enevoldsen 1987; Kullberg 1993; Lee 1992; Lee 2021; Lestradet 1981; Mathiesen 1990; McCance 1989; Nelson 1989; Nielsen 1984; Nordwall 2015; Pambianco 2006; Rodriguez‐Villalobos 2005; Rudnisky 2017; Skrivarhaug 2006; Styles 2000; Teuscher 1988; Valone 1981; Varma 2010; Vesteinsdottir 2010; Voigt 2018; Yokoyama 1994; Zavrelova 2011), and high in five studies (McCarty 2003; Miki 1969; Pirart 1977; Simonsen 1980; Verdaguer 2009). This was due to insufficient presentation of data to assess adequacy of analytic strategy (n = 20/59, 34%), inadequate statistical model for study design (n = 41/59, 69%), and potentially selective reporting of results (n = 10/59, 17%)

### Prognostic factors for progression to PDR

#### Demographic factors

##### Gender  

Twenty‐five studies investigated gender: 10 studies in people with T1D (Arfken 1998; Janghorbani 2000; Lestradet 1981; Lloyd 1995; Porta 2001; Roy 2006; Skrivarhaug 2006; Verdaguer 2009; WESDR: Klein 1989b, 1994b, 1998, 2008; Yokoyama 1994); 10 studies in people with T2D (Ballard 1986; Gange 2021; Gui 2013; Janghorbani 2000; Kalter‐Leibovici 1991; Lee 1992; Lee 2021; Nelson 1989; Rudnisky 2017; WESDR: Klein 1989a, 1994b); and five studies in mixed populations of T1D and T2D (Harris 2013; Janghorbani 2000; Jeng 2016; Keen 2001; Lee 2017). 

Six studies undertook multivariable regression analyses (Gange 2021; Harris 2013; Jeng 2016; Lee 2017; Lee 2021; Nelson 1989) (Table 2). Gender was not found to be an independent predictor of PDR in any of these studies (Table 1).

It was only possible to undertake meta‐analysis of unadjusted effect estimates from two studies ‐ one with a mixed population of T1D and T2D (Harris 2013) and one with an unspecified DM diagnosis population (Jeng 2016) ‐ which we determined were sufficiently homogeneous with respect to study duration, type of analyses, and effect estimate provided. The pooled HR was 1.09 (95% CI 0.96 to 1.23) (Figure 4), which was consistent with the findings from multivariable analyses, in that gender was not likely to increase risk of developing PDR, with a moderate certainty of evidence.

**4:**
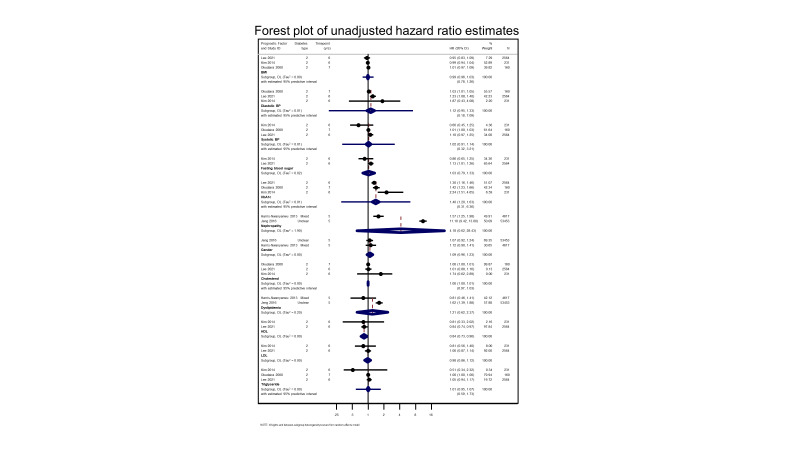
Forest plot of unadjusted hazard ratio estimates

Other studies compared incidence of PDR based on gender in T1D (Arfken 1998; Janghorbani 2000; Lestradet 1981; Lloyd 1995; Porta 2001; Roy 2006; Skrivarhaug 2006; WESDR: Klein 1989b, 1994b, 1998 and 2008); Yokoyama 1994) and T2D (Ballard 1986; Gange 2021; Gui 2013; Janghorbani 2000; Kalter‐Leibovici 1991; Lee 1992; Rudnisky 2017; WESDR: Klein 1989a and 1994b). All, with one exception (Yokoyama 1994), similarly concurred that gender was not associated with development of PDR. The study by Yokoyama and colleagues, which included 373 participants and was conducted over the longest follow‐up period of 35 years, found a cumulative incidence of PDR of 65% in males and 81% in females. Female participants also developed PDR significantly faster than males (P < 0.002) (Yokoyama 1994). 

##### Ethnicity

One study in people with T1D (Arfken 1998), three in people with T2D (Gange 2021; Kalter‐Leibovici 1991; Lee 2017), and one in a mixed population of people with T1D and T2D (Harris 2013) investigated ethnicity as a risk factor for PDR. Meta‐analysis was not possible given the heterogeneity of the studies.

Four studies undertook multivariable regression (Arfken 1998; Harris 2013; Kalter‐Leibovici 1991; Lee 2017) (Table 3). In Arfken’s study, African American ethnicity was not statistically significantly associated with progression to PDR when compared to Caucasian (understood to be White) ethnicity  (Arfken 1998). The Lee 2017 study included people of non‐Caucasian (understood to be non‐White) ethnicity (no further details provided; it states that 63.97% of the baseline population was White) and ethnicity was not found to have an effect on risk of PDR (Lee 2017). One study found non‐Ashkenazi Jews were at increased risk of developing PDR when compared to Ashkenazi Jews (Kalter‐Leibovici 1991) (Table 1). The certainty of evidence was, however, very low due to risk of bias, inconsistency, and imprecision. 

The Harris 2013 study compared proportion of participants progressing and not progressing to PDR in Whites, Blacks, Latinos, and Asians, and found no statistically significant differences. In further analyses, Black ethnicity was "statistically significantly" associated with progression to PDR in univariable regression analysis (HR 1.41, 95% CI 1.01 to 1.96; P < 0.05), as quoted in the publication, but not in multivariable regression analysis (Harris 2013).

Gange and colleagues undertook univariable analysis only and found people of Hispanic ethnicity (P = 0.003) to be at increased risk of developing PDR, whilst those of White ethnicity had reduced risk (P = 0.005) (Gange 2021).

##### Age at diagnosis of DM

Fourteen studies ‐ eight in people with T1D (Kalter‐Leibovici 1991; Nielsen 1984 (Report I); McCance 1989; Porta 2001; Styles 2000; Verdaguer 2009; WESDR: Klein 1998, 2008); Yokoyama 1994), four in people with T2D (Ballard 1986; Gange 2021; Lee 1992; Okudaira 2000), and two in a mixed population of people with TID and T2D (Janghorbani 2000; Pirart 1977) ‐ evaluated age at diagnosis of DM as a risk factor for PDR. Meta‐analysis was not possible due to heterogeneity among studies.

Two studies ‐ Porta 2001 and Gange 2021 ‐ undertook multivariable regression analyses (Table 4). Porta and colleagues found age of diagnosis of DM at less than 12 years to be an independent risk factor for progression to PDR in people with T1D (Porta 2001). Gange and colleagues evaluated the development of PDR in the five years following diagnosis of T2D, and found that those diagnosed between 65 and 74 years of age were at higher risk compared to those diagnosed at aged 18 to 64 years and at age 75 years or higher (Gange 2021) (Table 1). However, we rated the certainty of the evidence as low due to a moderate to high risk of bias in the studies and a lack of studies preventing the grading of consistency.  

The other studies on T1D (Kalter‐Leibovici 1991; McCance 1989; Styles 2000; Verdaguer 2009; WESDR) and T2D (Ballard 1986; Lee 1992; Okudaira 2000) undertook univariate analyses only, and found age at diagnosis of DM not to be associated with progression to PDR. The exception to this was the Yokoyama 1994 study which found that participants diagnosed with DM at zero to eight years of age have a "statistically significantly" reduced risk of development of PDR than those diagnosed at nine to 17 years of age (P < 0.001) and 18 to 29 years of age (P < 0.001). However, only descriptive statistics were undertaken, and the study included fewer participants (n = 373) than Porta 2001 (n = 2013), but was conducted over a longer period (35 years) (Yokoyama 1994). 

In a mixed cohort of 3482 participants with T1D and T2D followed for five years, Janghorbani 2000 found, using crude Cox regression coefficients, that being diagnosed at 30 years old or older compared to under 30 years of age was a risk factor for developing PDR (RR 1.24, 95% CI 1.09 to 1.41; P < 0.001). However, using an analysis of variance approach, they found participants who developed PDR were diagnosed with DM at a mean age of 38.2 years (standard deviation (SD) 18.4) when compared with those that did not (mean age of 42.5 years; SD 19.7). According to the publication, the mean difference was "statistically significant" (P < 0.001) (Janghorbani 2000).

##### Duration of diabetes

Duration of diabetes was the most frequently evaluated prognostic factor for PDR (n = 25 studies): 15 studies in people with T1D (Grauslund 2009; Janghorbani 2000; Kalter‐Leibovici 1991; Kullberg 1993; Lloyd 1995; McCance 1989; Nielsen 1984; Pambianco 2006; Porta 2001; Roy 2006; Skrivarhaug 2006; Styles 2000; Verdaguer 2009; WESDR; Yokoyama 1994); 11 studies in people with T2D (Chen 1995; Cho 2019; Gui 2013; Janghorbani 2000; Kim 1998; Kim 2014; Lee 1992; Nelson 1989; Nielsen 1984; WESDR; Zavrelova 2011); and three in mixed populations of people with T1D and T2D (Keen 2001; Janghorbani 2000; Varma 2010). Janghorbani 2000 reported findings separately for T1D and T2D, as well as for the combined cohort, but Keen 2001 and Varma 2010 only reported on the combined population (T1D and T2D). The WESDR study reported findings on the significance of duration of diabetes in five separate publications with outcomes at four (WESDR: Klein 1989b), 10 (WESDR: Klein 1994b), 14 (WESDR: Klein 1998) and 25 (WESDR: Klein 2008) years in participants with T1D, and at four (WESDR: Klein 1989a) and 10 (WESDR: Klein 1994b) years in those with T2D. Due to heterogeneity amongst studies, meta‐analysis was not possible.  

Ten studies undertook multivariable regression analyses (Grauslund 2009; Gui 2013; Janghorbani 2000; Kalter‐Leibovici 1991; Keen 2001; Kim 1998; Kim 2014; Lee 1992; Lloyd 1995; Porta 2001) (Table 5). In the studies on T1D (Grauslund 2009; Janghorbani 2000; Kalter‐Leibovici 1991; Lloyd 1995; Porta 2001), duration of DM was only found to be an independent predictor of the development of PDR when DR severity at baseline was not included as a covariate in the models (Janghorbani 2000; Kalter‐Leibovici 1991; Porta 2001). When models were adjusted for DR severity at baseline, the effect of diabetes duration did not remain statistically significant (Grauslund 2009; Lloyd 1995; Porta 2001). 

In the studies on T2D, an association between longer duration of diabetes and increased risk of PDR was found only in studies which did not correct for HbA1c at baseline (Gui 2013; Lee 1992). None of the studies included DR severity at baseline in their models. The studies by Kim 1998 and Kim 2014 did not find duration of diabetes to be an independent predictor of development of PDR. Janghorbani and colleagues found increased risk of progression to PDR in insulin‐treated participants with 12 or more years' duration of diabetes (Janghorbani 2000).  

In mixed populations of people with T1D and T2D, Janghorbani 2000 found participants with eight or more years' duration of diabetes were at increased risk of developing PDR. Similarly, Keen 2001 found duration of diabetes (per five years) to be an independent predictor of PDR. Neither of these studies adjusted for DR severity at baseline (Janghorbani 2000; Keen 2001) (Table 1).

We downgraded the certainty of evidence for effect of duration of DM to very low due to risk of bias in the included studies, inconsistencies in effect estimates, and imprecision.

The other studies on T1D which undertook univariable regression analyses generally concluded that diabetes duration had an impact on the development of PDR; however, this was not necessarily linear (Janghorbani 2000; Kalter‐Leibovici 1991; McCance 1989; Nielsen 1984 (Report I); Pambianco 2006; Roy 2006; Verdaguer 2009; WESDR; Yokoyama 1994). Participants were most likely to develop PDR between 13 and 19 years of diabetes duration, with a decline in risk thereafter (Kalter‐Leibovici 1991; Roy 2006; Skrivarhaug 2006; WESDR: Klein 1989b; 1994b); Yokoyama 1994). However, in the longest‐term, 25‐year follow‐up of the WESDR study, Klein and colleagues did not find duration of DM to be associated with progression to PDR, with the cumulative incidence of 42% (95% CI 39 to 46) remaining relatively constant with duration of diabetes, whilst accounting for competing risk of death (WESDR: Klein 2008).

Studies on T2D which undertook univariable analyses only generally concurred that increased duration of DM increased the risk of development of PDR (Chen 1995; Gui 2013; Lee 1992; Nelson 1989; Nielsen 1984), with the exception of Cho 2019, Kim 1998, and Kim 2014. Kim 1998 had a very small sample size (n = 56). Cho 2019 included 405 participants followed for a mean of four years (SD 2.0); it was unclear how many progressed to PDR (OR 1.00, 95% CI 1.00 to 1.03; P value not provided). Kim 2014 included 231 participants followed for six years (HR 1.26, 95% CI 1.00 to 1.65; P = 0.07).

Two of the WESDR trial reports (904 participants) evaluated linear trends in the effect of diabetes duration on incidence of PDR in people with older‐onset T2D, which authors also subgrouped as requiring or not requiring insulin (WESDR: Klein 1989a and 1994b). Progression to PDR was only statistically significantly associated with duration of DM (P < 0.005) in the older‐onset group using insulin (WESDR: Klein 1989a and 1994b). Varma 2010 included 324 participants with T1D and T2D; only 17 developed PDR during the four‐year follow‐up. Duration of diabetes was not found to be statistically significantly associated with development of PDR (P = 0.08) (Varma 2010). 

##### Type of DM

Only two studies compared development of PDR in participants with T1D and T2D (Janghorbani 2000; Keen 2001). Both undertook multivariable regression analyses (Table 33). Janghorbani 2000 found T1D to be associated with a decreased risk of developing PDR. Similarly, Keen 2001 found T1D to be associated with decreased risk of progression to PDR, but only when fasting plasma glucose was included in the model. However, we downgraded the certainty of the evidence to very low due to risk of bias in included studies, inconsistencies in effect estimates, and imprecision. 

**29:** Type of diabetes ‐ Studies undertaking multivariable regression analyses to determine the effect of type of diabetes on progression to PDR

**Study**	**Study type**	**Time years **	**N at baseline**	**Adjustment factors**	**Effect estimate**			**P value**	**Comments**
					Type	Value	95% CI		
									
**Type 1 and 2 diabetes**									
Janghorbani 2000	Retrospective cohort	5	3482	HbA1c, DM duration, SBP, and proteinuria	RR	0.59^a^	0.48 to 0.71		
Keen 2001	Prospective cohort	8	4483	Duration of DM, sex, age, SBP, DBP, plasma cholesterol, BMI, smoking status, insulin treatment, vascular disease, renal disease	OR	1.07^a^ 0.53^a,b^		Nonsignificant < 0.01	

**BMI:** body mass index; **CI:** confidence interval; **DBP:** diastolic blood pressure; **DM:** diabetes mellitus; **DR:** diabetic retinopathy; **HbA1c:** glycated haemoglobin/haemoglobin A1c; **HR:** hazard ratio; **NPDR:** non‐proliferative diabetic retinopathy; **OR:** odds ratio; **PDR:** proliferative diabetic retinopathy; **RR:** risk ratio; **SBP:** systolic blood pressure; **vs:** versus ^a^Type 1 diabetes ^b^Fasting plasma glucose included in model

##### Socioeconomic status

Socioeconmic status as a risk factor for PDR was investigated in two studies in people with T1D (Roy 2006; WESDR: Klein 1994a), four in people with T2D (Chen 1995; Gange 2021; Kalter‐Leibovici 1991; WESDR: Klein 1994a), and one in a mixed population of people with T1D and T2D (Harris 2013). The studies used different methods to assess socioeconomic status and, thus, meta‐analysis was not possible. With the exception of the Kalter‐Leibovici 1991 study, socioeconomic status was not found to be a risk factor for PDR (Chen 1995; Gange 2021; Harris 2013; Roy 2006; WESDR: Klein 1994a).

Only the WESDR study undertook multivariable regression analyses (WESDR: Klein 1994a) (Table 6). Socioeconomic status was determined using the Duncan Socioeconomic Index, which assigns a score according to occupation or the spouse’s occupation, if married but not working, with higher scores indicating higher socioeconomic status (Stevens 1981). Socioeconomic status was not associated with incidence of PDR in males or females in T1D or T2D (WESDR: Klein 1994a). We downgraded the certainty of the evidence to very low due to risk of bias in included studies and imprecision. It was not possible to grade consistency due to only one study having undertaken multivariable regression analysis (Table 1). 

The study by Kalter‐Leibovici and colleagues, which included 330 Jewish participants with T1D at baseline followed for 10 years, was the only study finding a negative correlation between socioeconomic status and progression to PDR. Using an analysis of variance, an increased percentage of participants who progressed to PDR had a family income of less than the national average (P < 0.001) (Kalter‐Leibovici 1991). 

##### Education level

Only two studies evaluated the effect of education, as a stand‐alone variable, on the development of PDR (Gange 2021; WESDR: Klein 1994a). Meta‐analysis was not possible due to heterogeneity between the studies.

Only the WESDR study undertook multivariable regression analysis at the four‐year follow‐up (WESDR: Klein 1994a) (Table 7). Different levels of education were considered: no high school degree, high school degree, some college, and college graduate; males and females were analysed separately. In females in the younger‐onset group, for every five or more years of education, there was a statistically significantly decreased probability of developing PDR, but this was not the case in males. In the older‐onset group, education was not associated with incidence of PDR in males or females (WESDR: Klein 1994a). We downgraded the certainty of the evidence to very low due to risk of bias in the included studies and imprecision. It was not possible to grade consistency due to only one study having undertaken multivariable regression analysis (Table 1).

In the WESDR report on outcomes at 25 years, males and females were considered collectively (n = 481) and only univariate analysis was undertaken. Education level (per four years) was not associated with incidence of PDR (HR 1.05, 95% CI 0.94 to 1.19; P = 0.38) (WESDR: Klein 2008). 

Gange and colleagues conducted a study involving 71,817 participants with T2D, followed for five years. Only univariate analysis was undertaken and, although there was a trend indicating that participants with PDR had lower levels of education, differences were not statistically significant (P > 0.05) (Gange 2021).

#### Systemic factors

##### Glycated haemoglobin (HbA1c)

The relationship between HbA1c level and the development of PDR was assessed in 24 studies: 12 studies in people with T1D (Arfken 1998; Janghorbani 2000; Kullberg 1993; Lloyd 1995; McCance 1989; Nordwall 2015; Porta 2001; Roy 2006; Skrivarhaug 2006; Styles 2000; Verdaguer 2009; WESDR: Klein 1988, 1994, 1998, 2008); 10 studies in people with T2D (Chen 1995; Cho 2019; Gange 2021; Gui 2013; Kalter‐Leibovici 1991; Kim 2014; Lee 2021; Okudaira 2000; WESDR: Klein 1988, 1994); Zavrelova 2011); and two studies in a mixed population of T1D and T2D (Harris 2013; Janghorbani 2000). 

Eight studies on T1D undertook multivariable logistic regression analyses (Arfken 1998; Janghorbani 2000; Kullberg 1993; Lloyd 1995; Porta 2001; Roy 2006; Skrivarhaug 2006; WESDR: Klein 1988a, 1994c, 1998, 2008). All studies found that increased HbA1c levels were associated with progression to PDR. DR severity at baseline was included as a co‐variate in most of the studies (Arfken 1998; Lloyd 1995; Porta 2001; Skrivarhaug 2006; WESDR: Klein 1988, 1994, 1998) (Table 9). 

Eight studies on T2D undertook multivariable logistic regression analyses (Cho 2019; Gange 2021; Kalter‐Leibovici 1991; Kim 1998; Kim 2014; Lee 2021; Okudaira 2000; WESDR: Klein 1988, 1994c). Similarly to findings in T1D, these studies generally found a positive correlation between increased HbA1c and incidence of PDR. The exceptions were the studies by Cho 2019 and Lee 2021, which found HbA1c level to be statistically significantly associated with progression to PDR in univariable, but not in multivariable, regression analyses. Only two studies corrected for DR severity at baseline (Lee 2021; WESDR: Klein 1988, 1994c) (Table 9). 

It was only possible to undertake meta‐analysis of unadjusted effect estimates combining three studies including a total of 2955 participants with T2D, followed for six to seven years (Kim 2014; Lee 2021; Okudaira 2000). None of these studies included DR severity at baseline in their models. The pooled HR was 1.40 (95% CI 1.20 to 1.63) and the 95% prediction interval ranged from 0.31 to 6.36 (Figure 4).

Two studies on mixed populations of people with T1D and T2D undertook multivariable regression analyses (Harris 2013; Janghorbani 2000). Harris and colleagues found that for every one per cent point increase in HbA1c, the risk of developing PDR was increased by 14% (Harris 2013). Janghorbani and colleagues found that HbA1c of 11% or higher was an independent predictor of PDR. Additionally, the mean difference in HbA1c values between non‐progressors and progressors to PDR was also statistically significant (MD 0.8%, 95% CI 0.42 to 1.18; P < 0.001), as quoted in the publication (Janghorbani 2000). Neither of these studies corrected for DR severity at baseline (Table 8). We rated the evidence for HbA1c as of moderate certainty because of moderate to high risk of bias in the studies included in the analyses. 

In general, studies using univariable analyses supported the above findings (McCance 1989; Nordwall 2015; Verdaguer 2009). The exception to this was the Styles 2000 study, which had the longest follow‐up (45 years) and did not find a significant mean difference in HbA1c values between progressors (mean 8.3%; range 5.8 to 12.3) and non‐progressors (mean 8.7%; range 6.7 to 11.9) to PDR (P = 0.16) (Styles 2000). 

Some studies attempted to establish whether there was an HbA1c threshold below which progression to PDR would not occur, but this threshold was not found (Porta 2001; Roy 2006; WESDR: Klein 1988, 1994c). However, a longer‐term study by Nordwall and colleagues recommended HbA1c to be below 7.6% to prevent PDR, as none of the 451 participants in their study with levels below 7.6% developed PDR during the study period of 22 years (Nordwall 2015).

The WESDR study evaluated whether there was a relationship between change in glycaemic control and the risk of progression to PDR, whilst controlling for DR severity and HbA1c at baseline, and hypertension. The OR for a one percentage point increase in HbA1c from baseline to four‐year follow‐up was 1.33 for progression to PDR, equivalent to a 25% increase in the 14‐year incidence of PDR (WESDR: Klein 1998). In the longest‐term report of 25 years, a one percentage point decrease in HbA1c from baseline to four‐year follow‐up was associated with an 18% decrease in the 21‐year rate of progression to PDR (WESDR: Klein 2008). The data from the WESDR reports also suggested that reducing levels of HbA1c, even later during DM, or even when moderate NPDR is present, may reduce the risk of progression to PDR already conferred by higher HbA1c in previous years (WESDR: Klein 1994c, 1998).

The Gui 2013 study included 190 participants with T2D followed for two years and found higher mean HbA1c in progressors (mean 11.43%; SE 3.09) compared to non‐progressors (mean 7.43%; SE 3.14) to PDR, but the difference was not significant (P = 0.1) (Gui 2013). Zavrelova and colleagues found mean HbA1c values for progressors (9.1%) to be higher than that of non‐progressors (8.4%) (Zavrelova 2011). 

##### Fasting plasma glucose

Seven studies evaluated fasting plasma glucose as a prognostic factor for the development of PDR: six in participants with T2D (Ballard 1986; Chen 1995; Cho 2019; Kim 1998; Lee 1992; Lee 2021), and one in a mixed population of people with T1D and T2D (Keen 2001).

Three studies undertook multivariable regression analyses (Keen 2001; Lee 1992; Lee 2021) (Table 10). In T2D, Lee and colleagues did not find fasting plasma glucose to be an independent predictor for the incidence of PDR (Lee 2021). Conversely, in the Lee 1992 study, fasting plasma glucose was predictive of the development of PDR (Lee 1992). However, HbA1c and DR severity at baseline were not adjusted for in the latter study (Lee 1992). In a mixed population of T1D and T2D, Keen and colleagues also found fasting plasma glucose to be significantly associated with development of PDR, but again, HbA1c and DR severity at baseline were not included as covariates (Keen 2001) (Table 10). Overall, evidence from multivariable regression analysis is very uncertain about the effect of fasting plasma glucose on the risk of developing PDR (Table 8).

It was only possible to undertake meta‐analysis of unadjusted effect estimates from two studies of T2D (Kim 2014; Lee 2021), which we determined to be sufficiently homogeneous with respect to study duration, type of analyses, and effect estimate provided. The pooled HR was 1.03 (95% CI 0.79 to 1.33) (Figure 4).

Some studies undertook univariable regression analyses. Ballard 1986 and Chen 1995 evaluated cumulative incidence with fasting plasma glucose and determined a positive correlation between increasing levels of fasting plasma glucose and development of PDR. In univariate analysis, Cho 2019 and Lee 2021 did not identify fasting plasma glucose as being associated with progression to PDR. The Lee 1992 study compared the mean level of fasting plasma glucose in progressors (12.5 mmol/L) versus non‐progressors (9.6 mmol/L) to PDR, and identified a difference in values (P < 0.001) (Lee 1992). Conversely, the Kim 1998 study did not detect any difference in mean fasting plasma glucose between the group that developed PDR (12.6 mmol/L; SD 5.7) and the group that remained stable (11.5 mmol/L; SD 4.5) (Kim 1998).

##### Diastolic blood pressure (DBP)

Twelve studies evaluated the relationship between DBP and the incidence of PDR: four in people with T1D (Grauslund 2009; Porta 2001; Roy 2006; WESDR); seven in people with T2D (Kim 1998; Kim 2014; Lee 1992; Lee 2021; Okudaira 2000; WESDR; Zavrelova 2011); and two in a mixed population of participants with T1D and T2D (Janghorbani 2000; Keen 2001). 

Seven studies undertook multivariable regression (Grauslund 2009; Keen 2001; Lee 2021; Okudaira 2000; Porta 2001; Roy 2006; WESDR: Klein 1989c) (Table 11). 

In T1D, DBP was only found to be an independent predictor of development of PDR when DR severity at baseline was not included in the models (Porta 2001; Roy 2006). The WESDR study and the Grauslund 2009 study included DR severity at baseline and DBP was not found to be statistically significantly associated with progression to PDR (Grauslund 2009; WESDR: Klein 1989c). 

In T2D, only two studies undertook multivariable regression analyses (Lee 2021; Okudaira 2000), and found that DBP was predictive of the development of PDR. However, only the Lee 2021 study corrected for DR severity at baseline. Evidence from multivariable regression analyses suggesting that DBP is associated with progression to PDR is very uncertain (Table 8). 

Two studies on T2D were appropriate for meta‐analysis with respect to study duration, type of analyses, and effect estimate provided (Hsieh 2018; Lee 2021). The pooled HR of adjusted HR estimates (HbA1c was the only common adjustment factor) was 1.07 (95% CI 0.96 to 1.18) (Figure 4). 

In T1D and T2D, Keen 2001 found no association between DBP (per 5 mmHg increase) and PDR in multivariable regression analysis. 

Other studies undertook univariable regression analyses only. The WESDR study also evaluated linear trends and found that increasing DBP was positively correlated with increasing incidence of PDR in T1D at four (P < 0.001), 10 (P < 0.001), and 14 (P < 0.001) years of follow‐up (WESDR: Klein 1989c, 1995a, 1998). In the 25‐year report, DBP (per 10 mmHg increase) was found to be associated with incidence of PDR (HR 1.3, 95% CI 1.16 to 1.46; P < 0.001) at the univariable level (not considered in multivariable analysis in this report) (WESDR: Klein 2008). 

In participants with T2D, at four years' follow‐up, the relationship between increasing DBP and incidence of PDR was not significant, as evaluated by linear trends, even after taking into account the use of antihypertensive medications (WESDR: Klein 1989c). At ten years, higher levels of DBP were associated with increased risk of developing PDR (P < 0.05) in the older‐onset group taking insulin. In other studies on T2D which undertook univariate analyses only (Kim 1998; Kim 2014; Lee 1992; Zavrelova 2011), DBP was not found to be a risk factor for PDR. 

The Janghorbani 2000 study (n = 3482 T1D and T2D; follow‐up of five years) found DBP of between 85 mmHg and 94 mmHg was associated with an increased risk of progression to PDR (P < 0.05), but levels higher than 95 mmHg were not. Using analysis of variance, they found participants who progressed to PDR had lower levels of DBP (75.1 mmHg; SD 28.4) compared to those who did not progress to PDR (78.3 mmHg; SD 23.4) (P < 0.05) (Janghorbani 2000). 

##### Systolic blood pressure (SBP)

Thirteen studies in participants with T1D (Arfken 1998; Grauslund 2009; Janghorbani 2000; Porta 2001; Roy 2006; WESDR: Klein 1989c, 1998, 2008), T2D (Kim 1998; Kim 2014; Lee 1992; Lee 2021; Okudaira 2000; WESDR: Klein 1989c; Zavrelova 2011), and mixed populations of both T1D and T2D (Janghorbani 2000; Keen 2001) evaluated SBP as a risk factor for PDR.

Eight studies undertook multivariable regression (Grauslund 2009; Janghorbani 2000; Keen 2001; Lee 1992; Lee 2021; WESDR: Klein 1989, 1995a, 2008) (Table 12). 

In T1D, SBP was only found to be an independent predictor of development of PDR when DR severity at baseline was not included in the models (Janghorbani 2000; WESDR: Klein 2008). In the studies by Grauslund and WESDR, which included DR severity at baseline, SBP was not significantly associated with progression to PDR (Grauslund 2009; WESDR: Klein 1989c, 1995a). 

In the studies on T2D, SBP was not found to be an independent predictor of development of PDR (Lee 1992; Lee 2021; WESDR: Klein 1989c). Three studies on T2D were also appropriate for meta‐analysis with respect to study duration, type of analyses, and effect estimate provided (Kim 2014; Lee 2021; Okudaira 2000). The pooled HR of unadjusted HR estimates was 1.02 (95% CI 0.91 to 1.14). The 95% prediction interval ranged from 0.32 to 3.21 (Figure 4). 

Janghorbani and colleagues additionally reported findings for the entire cohort of participants with T1D and T2D: SBP above 160 mmHg was significantly associated with increased progression to PDR (Janghorbani 2000). In contrast, the Keen 2001 study, which also included people with T1D or T2D, did not find SBP (increments of 10 mmHg) to be an independent predictor for the development of PDR (Keen 2001). Neither study corrected for DR severity at baseline and Keen 2001 did not adjust for HbA1c. Overall, evidence from multivariable regression analyses suggesting that DBP is associated with progression to PDR is very uncertain (Table 8).

In univariable analyses, the WESDR study additionally reported a statistically significant linear trend in the incidence of PDR in participants with T1D, with increasing SBP at the four‐, 10‐, and 14‐year periods (P < 0.001) (WESDR: Klein 1989c, 1995a, Report 1998) (linear trend analysis not reported in the WESDR Klein 2008 25‐year outcomes). Other potential confounders were not considered in these analyses. 

Other studies undertook univariable regression analyses only (Arfken 1998; Porta 2001; Roy 2006). Roy 2006 found that participants with T1D in the upper quartile of SBP (≥ 135 mmHg) at baseline had increased risk of progression to PDR during the six‐year follow‐up than those in the lowest quartile (≤ 110 mmHg) (OR 3.09, 95% CI 1.37 to 7.00; P = 0.05) (Roy 2006). Arfken 1998 followed 312 participants for six years and found an increase in SBP in participants progressing to PDR (mean 120 mmHg, SD 21) compared to those that did not (mean 111, SD 16; P = 0.01) (Arfken 1998). Conversely, when comparing participants who progressed to PDR (median: 119 mmHg; 25thand 75th percentiles: 108 and 134, respectively) with those that did not (median: 117 mmHg; 25thand 75th percentiles: 108 and 128, respectively) during a seven‐year observation period, Porta 2001 did not find statistically significant differences in SBP between groups (P = 0.10).

In the WESDR study, in analysis of linear trends at four years, SBP was not associated with incidence of PDR in either the insulin‐taking or non‐insulin‐taking groups (WESDR: Klein 1989c). At 10 years, in the older‐onset group taking insulin, higher SBP was significantly associated with development of PDR (P < 0.05), but not in the older‐onset group not taking insulin (P = 0.20) (WESDR: Klein 95a). 

##### Mean arterial pressure

Two studies considered the effect of mean arterial blood pressure on the development of PDR (Chen 1995; Roy 2006). 

In multivariable analysis, including HbA1c, age, sex, socioeconomic status, BMI, central retinal artery equivalent (CRAE), ocular perfusion pressure, and refractive error as covariates, Roy 2006 determined that mean arterial blood pressure (per 10 mmHg change) was associated with incidence of PDR during the six‐year study period (OR 1.35, 95% CI 0.91 to 2.00; P < 0.001). However, although the P value was statistically significant, the 95% CIs ranged from being protective to determining increased risk (Table 8). In the Chen 1995 study (follow‐up period not reported), the cumulative incidence of PDR was not associated with increasing quartiles of mean arterial blood pressure (P = 0.13), but other variables were not corrected.

##### Dyslipidaemia

Four studies considered the effect of dyslipidaemia on progression to PDR (Gange 2021; Harris 2013; Jeng 2016; Verdaguer 2009). The Gange 2021 and Verdaguer 2009 studies included participants with T1D and T2D, respectively, and the Harris 2013 study a mixed cohort of participants with T1D and T2D; Jeng 2016 did not provide information on type of diabetes. None found an association between dyslipidaemia and incidence of PDR (Gange 2021; Harris 2013; Jeng 2016; Verdaguer 2009). 

We undertook multivariable regression analysis in only two of these studies, which were sufficiently homogeneous (Harris 2013; Jeng 2016) (Table 13) (Table 8). The pooled HR of the unadjusted effect estimates was 1.21 (95% CI 0.62 to 2.37), consistent with the negative correlation between dyslipidaemia and development of PDR found in the other studies (Figure 4).

The remaining two studies undertook univariable analyses only (Gange 2021; Verdaguer 2009). Verdaguer 2009 included only 39 participants with T1D followed for 18 years. The study compared dyslipidaemia in the group with NPDR to the group with PDR and the difference was not significant (P = 0.133) (Verdaguer 2009). Gange 2021 included 71,817 participants with T2D. The percentage of participants with dyslipidaemia who progressed to PDR (25%) in the five‐year period subsequently to being diagnosed with DM was not significantly different to that of those who did not progress (25.6%, P = 0.61) (Gange 2021). 

##### Total cholesterol

Eleven studies evaluated total cholesterol as a risk factor for PDR: two studies in people with T1D (Porta 2001; Roy 2006); eight studies in people with T2D (Gui 2013; Kim 1998; Kim 2014; Lee 1992; Lee 2021; Nelson 1989; Okudaira 2000; Zavrelova 2011); and one study in a mixed group of people with T1D and T2D (Keen 2001). 

Five studies undertook multivariable regression analyses (Keen 2001; Lee 1992; Lee 2021; Nelson 1989; Porta 2001) (Table 14). Only Keen 2001 and Nelson 1989 found an association between increased total cholesterol and development of PDR. However, HbA1c and DR severity at baseline were not included in their models (Keen 2001; Nelson 1989) (Table 14).

The Lee 1992, Lee 2021, and Porta 2001 studies did not find total cholesterol to be an independent predictor of PDR. Porta 2001 found that mean total cholesterol level was elevated in participants who progressed to PDR (mean 5.6; SE 0.1) when compared to those that did not (mean 5.1; SE 0.03; P < 0.001). However, when adjustment for HbA1c and duration of diabetes was undertaken, the association was no longer "statistically significant" (reported descriptively) (Porta 2001). Overall evidence from multivariable regression analyses suggesting that total cholesterol is associated with progression to PDR is very uncertain (Table 8).

Additionally, meta‐analysis was possible in three other studies on T2D which we considered to be homogeneous with regard to study duration, type of analyses, and effect estimate provided (Kim 2014; Lee 2021; Okudaira 2000). The pooled HR of the unadjusted effect estimates was 1.00 (95% CI 1.00 to 1.01). The 95% prediction interval ranged from 0.97 to 1.03 (Figure 4).

Other studies included total cholesterol in univariable analyses only, and all but Roy 2006 did not find total cholesterol to be associated with PDR (Gui 2013; Kim 1998; Kim 2014; Okudaira 2000; Roy 2006; Zavrelova 2011). 

##### Triglycerides

Ten studies investigated the impact of triglyceride level on development of PDR: three studies in people with T1D (Lloyd 1995; Porta 2001; Skrivarhaug 2006), and seven studies in people with T2D (Gui 2013; Kim 1998; Kim 2014; Lee 1992; Lee 2021; Okudaira 2000; Zavrelova 2011). 

Three studies undertook multivariable regression (Lee 2021; Porta 2001; Skrivarhaug 2006) (Table 15). In T1D, triglycerides appeared to be an independent predictor of PDR (Porta 2001; Skrivarhaug 2006), but not in the study on T2D (Lee 2021) (Table 8). However, the certainty of evidence was low due to risk of bias in the included studies and imprecision in some studies (wide CIs).

Additionally, we undertook meta‐analysis combining three studies on T2D which were homogeneous with regard to study duration, analyses type, and effect estimates provided (Kim 2014; Lee 2021; Okudaira 2000). The pooled HR of the unadjusted effect estimates was 11.01 (95% CI 0.95 to 1.07), which was consistent with the non‐significant finding in the multivariable regression analysis (Lee 2021). The 95% prediction interval ranged from 0.59 to 1.73 (Figure 4).

Some studies undertook univariable analyses. In T1D, Lloyd 1995 found serum triglyceride levels (it is not reported whether participants were fasting) were elevated in the group that progressed to PDR (mean 2.0 mg/dL; SD 0.3) compared to the group that did not (mean 1.9 mg/dL; SD 0.2; P < 0.05) whilst adjusting for duration of diabetes (Lloyd 1995). Porta 2001 found triglycerides (fasting and non‐fasting levels) were increased in participants with T1D progressing to PDR (fasting and non‐fasting triglycerides: progressors 1.15 mmol/L versus non‐progressors 0.92 mmol/L; P < 0.001; fasting triglycerides: progressors 1.11 mmol/L versus non‐progressors 0.88 mmol/L; P < 0.001) (Porta 2001). 

The Lee 1992 study grouped participants with T2D as those with or without plasma triglyceride levels of 22.6 mg/dL or higher, and found the former had an increased risk of PDR (RR 1.7, 95% CI 1.1 to 2.62; P = 0.015). When stratified by duration of diabetes, triglyceride level remained significantly associated with development of PDR (P < 0.05) (Lee 1992). However, also in participants with T2D, Okudaira 2000 (HR 1, 95% CI 1 to 1; P = 0.90) and Kim 2014 (HR 0.51, 95% CI 0.34 to 2.32; P = 0.254) did not find triglyceride levels to be associated with incidence of PDR (Kim 2014; Lee 1992; Okudaira 2000). Similarly, studies which compared mean triglyceride values at baseline in progressors to PDR compared to non‐progressors did not find a statistically significant difference. Other potential risk factors were not corrected (Gui 2013; Kim 1998; Kim 2014; Zavrelova 2011). 

##### Low‐density lipoprotein (LDL) 

Seven studies evaluated the effect of LDL on the development of PDR: three in people with T1D (Lloyd 1995; Porta 2001; Roy 2006), and four in people with T2D (Gui 2013; Kim 2014; Lee 2021; Zavrelova 2011).

Only two studies undertook multivariable regression analyses (Lee 2021; Porta 2001). They did not find LDL to be an independent predictor for the development of PDR, but overall evidence was very uncertain about the effect (Lee 2021; Porta 2001) (Table 16) (Table 8). 

Additionally, we undertook meta‐analysis combining two studies in people with T2D which were sufficiently homogeneous with respect to study duration, analyses type, and effect estimates provided (Kim 2014; Lee 2021). The pooled HR of the unadjusted effect estimates was 0.98 (95% CI 0.86 to 1.12), which was consistent with the multivariable regression analysis by Lee 2021 which did not find LDL to be a prognostic factor for PDR (Figure 4).

Some studies undertook univariable analyses. In people with T1D, Roy 2006 found that higher LDL levels were associated with progression to PDR. Participants with LDL levels at baseline in the upper quartile had approximately three times the rate of progression to PDR than those in the lowest quartile (P = 0.02) (Roy 2006). Also in people with T1D, Lloyd 1995 determined that progressors to PDR had a higher LDL (mean value 121.3 mg/dL, SD 31.1) than non‐progressors (mean value 106.1 mg/dL, SD 28.6; P < 0.05) when adjusting for duration of diabetes only. 

In the studies in people with T2D, there was no significant difference in mean values of LDL in progressors to PDR compared to non‐progressors (Gui 2013; Kim 1998; Zavrelova 2011). 

##### High‐density lipoprotein (HDL)

Five studies evaluated the impact of HDL on the development of PDR: one study in people with T1D (Porta 2001), and four studies in people with T2D (Kim 1998; Kim 2014; Lee 2021; Zavrelova 2011). 

Only two studies undertook multivariable regression analyses and neither found HDL to be an independent predictor of PDR, but overall evidence was very uncertain about the effect (Lee 2021; Porta 2001) (Table 17) (Table 8). 

Additionally, we undertook meta‐analysis combining two studies in people with T2D which were sufficiently homogeneous with respect to study duration, analyses type, and effect estimates provided (Kim 2014; Lee 2021). The pooled HR of the unadjusted effect estimates was 0.84 (95% CI 0.73 to 0.96), suggesting a reduced risk of PDR with higher HDL values (Figure 4).

The remaining two studies undertook univariable analyses only, and only in people with T2D. These studies found reduced mean HDL levels in progressors to PDR versus non‐progressors (Kim 1998; Zavrelova 2011). 

##### Fibrinogen

Only two studies including people with T1D explored the effect of fibrinogen on progression to PDR (Lloyd 1995; Porta 2001). Both compared fibrinogen levels in progressors with those in non‐progressors to PDR. Lloyd 1995 identified an increased fibrinogen level in progressors (mean value 2.5, SD 0.1) compared to non‐progressors (mean value 2.4, SD 0.1; P < 0.01). In contrast, Porta 2001 found no statistically significant difference in fibrinogen levels between progressors (mean value 3.22, SE 0.1) and non‐progressors (mean value 3.17, SE 0.03; P = 0.6)). The effect of other potential risk factors was not considered in these analyses, except for duration of DM which was included in Lloyd 1995.

##### Biomarkers of renal function

The included studies that looked at this risk factor adopted different ways of assessing the effect of kidney function on the development of PDF. We describe these below.

###### Nephropathy

Five studies considered the effect of nephropathy or renal disease on the incidence of PDR: two in people with T2D (Gange 2021; Lee 1992), and three in mixed populations of people with T1D or T2D (Harris 2013; Jeng 2016; Keen 2001). 

Four studies undertook multivariable regression analyses (Gange 2021; Harris 2013; Jeng 2016; Keen 2001) (Table 18). In the study on T2D, renal disease was found to be an independent risk factor for PDR (Gange 2021). In the studies on mixed populations, the results were variable. Jeng 2016 and Keen 2001 identified a positive correlation between diabetic nephropathy and incidence of PDR. However, their definitions of nephropathy differed. Jeng 2016 defined nephropathy as “persistent albuminuria, progressive decline of GFR [glomerular filtration rate], and elevation of BP [blood pressure]”, whereas Keen 2001 assessed the intensity of protein precipitation in urine as an indicator of renal disease severity. Harris 2013 did not find a statistically significant association between renal disease and development of PDR. We assessed the available evidence based on multivariable regression analyses as very uncertain about the effect of nephropathy on the risk of developing PDR, due to risk of bias, inconsistency, and imprecision in studies (Table 8).  

The Harris 2013 and Jeng 2016 studies were sufficiently homogeneous to meta‐analyse. The pooled HR of the unadjusted estimates was 4.18 (95% CI 0.62 to 28.43), suggesting that the effect of renal disease on progression was not significant (Figure 4).

Other studies undertook univariable regression analyses in T2D. Whereas Gange 2021 and Harris 2013 found an increased percentage of progressors to PDR in people with renal disease (Gange 2021: 6.7% versus 1.6%; P < 0.001; Harris 2013: 39.1% versus 26%; P < 0.05), Lee 1992 did not identify a relationship between renal disease (defined as creatinine > 133 micrometre [µM] or proteinuria) and the development of PDR (RR 1.19, 95% CI 0.72 to 1.97; P = 0.501). 

###### Proteinuria

Seven studies studied the impact of proteinuria on the incidence of PDR: four studies in people with T1D (Grauslund 2009; Kofoed‐Enevoldsen 1987; Roy 2006; WESDR: Klein 1993, 1998, 2008), reporting outcomes at four, 14, and 25 years' follow‐up, in separate publications); three studies in people with T2D (Chen 1995; Nelson 1989; WESDR); and one study in people with T1D or T2D (Janghorbani 2000). Meta‐analysis was not possible due to study heterogeneity.

Five studies undertook multivariable regression (Grauslund 2009; Janghorbani 2000; Nelson 1989; Roy 2006; WESDR: Klein 1993, Reports XVII and XXII)) (Table 19). All but Grauslund 2009 and WESDR (Klein 1993) found proteinuria to be an independent predictor of PDR. Again, we deemed the certainty of evidence based on multivariable regression analyses to be very uncertain due to risk of bias, inconsistency, and imprecision in studies (Table 8). 

In the WESDR study reporting outcomes in people with T1D at four years, the presence of gross proteinuria (defined as urine protein concentration of 0.30 g/L or greater as measured by a reagent strip) at baseline was associated with increased risk of PDR. However, this association was only of borderline significance in people with no or mild NPDR at baseline (WESDR: Klein 1993). At 14 years (WESDR: Klein 1998) and 25 years (WESDR: Klein 2008), gross proteinuria at baseline remained a statistically significant risk factor for the development of PDR. Roy 2006 determined that participants with overt proteinuria (albumin excretion rate (AER) > 200 µg/min) at baseline had four times the risk of developing PDR.

In people with T2D, Nelson 1989 found proteinuria (protein‐to‐creatinine ratio of ≥ 113 mg/mmol) to be statistically significantly associated with development of PDR. However, the WESDR study found that, in the older‐onset group taking insulin with moderate or severe NPDR, gross proteinuria was not correlated with incidence of PDR (WESDR: Klein 1993). 

###### Albumin excretion rate (AER)

Five studies investigated the effect of AER on progression to PDR: four studies in people with T1D (Lloyd 1995; Mathiesen 1990; Porta 2001; Roy 2006), and one study in people with T2D (Kim 1998). Meta‐analysis was not possible due to the heterogeneity of these studies. 

Three studies undertook multivariable regression analyses (Kim 1998; Porta 2001; Roy 2006) (Table 20). Both studies including people with T1D found higher AER to be an independent predictor of PDR (Porta 2001; Roy 2006). In the study on T2D (Kim 1998), baseline AER was not associated with development of PDR.(Table 8).

Other studies undertook univariable analyses. In people with T1D, the Lloyd 1995 study compared mean AER in progressors to that in non‐progressors to PDR, whilst adjusting for duration of DM. Those who developed PDR had a statistically significantly increased mean AER (progressors versus non‐progressors: mean 1.7 LOG, SD 1.0 versus 1.2 LOG, SD 0.7; P < 0.001) (Lloyd 1995). Porta 2001 also found a significantly increased mean AER in progressors compared to non‐progressors (progressors versus non‐progressors: median 29 µg/min versus 12 µg/min; P < 0.001) (Porta 2001). Similarly, in people with T2D, the Kim 1998 study found that participants who progressed to PDR had increased AER at baseline compared to the non‐progressors (progressors versus non‐progressors: mean 67 µg/min, SD 61 versus 23 µg/min, SD 29; P < 0.05) (Kim 1998). 

###### Albumin creatinine ratio

Only three studies (Hsieh 2018; Kim 2014; Zavrelova 2011), all in people with T2D, evaluated the effect of values of albumin creatinine ratio on incidence of PDR. Meta‐analysis was not possible due to the studies' heterogeneity. 

Only two studies undertook multivariable regression analyses (Hsieh 2018; Kim 2014). Both studies found increasing albumin creatinine ratio to be an independent predictor of PDR (Hsieh 2018; Kim 2014) (Table 21). The certainty of the evidence is moderate, suggesting albumin creatinine ratio is likely associated with increased risk of progression to PDR in T2D (Table 8).

###### Estimated glomerular filtration rate (eGFR)

Two studies (Cho 2019; Hsieh 2018), both in people with T2D, evaluated the effect of eGFR on development of PDR and both undertook multivariable regression analyses (Table 22) (Table 8). Meta‐analysis was not possible due to their heterogeneity. Cho 2019 found a reduction in eGFR of more than 20% (but not in mean eGFR) to be associated with development of PDR. Hsieh 2018 found that participants with decreased mean follow‐up eGFRs were at increased risk of incident PDR.

###### Creatinine

Six studies considered the effect of creatinine on incidence of PDR: one studies in people with T1D (Styles 2000); four studies in people with T2D (Hsieh 2018; Lee 2021; Nelson 1989; Zavrelova 2011); and one in a mixed population of people with T1D and T2D. Three studies undertook multivariable regression analyses, all in T2D (Hsieh 2018; Lee 2021; Nelson 1989) (Table 23). Hsieh 2018 and Nelson 1989 found elevated creatinine levels to be an independent predictor of PDR, in contrast to the Lee 2021 study. However, we deemed the certainty of evidence as very low, due to risk of bias in included studies, inconsistency, and imprecision (Table 8). The Hsieh 2018 and Lee 2021 studies were appropriate for meta‐analysis with respect to study duration, type of analyses, and effect estimate provided, with HbA1c, age, gender, and BMI being common adjustment factors. The pooled HR of adjusted HR estimates was 1.61 (95% CI 0.77 to 3.36), although there appeared to be heterogeneity between the studies (Figure 5). 

**5:**
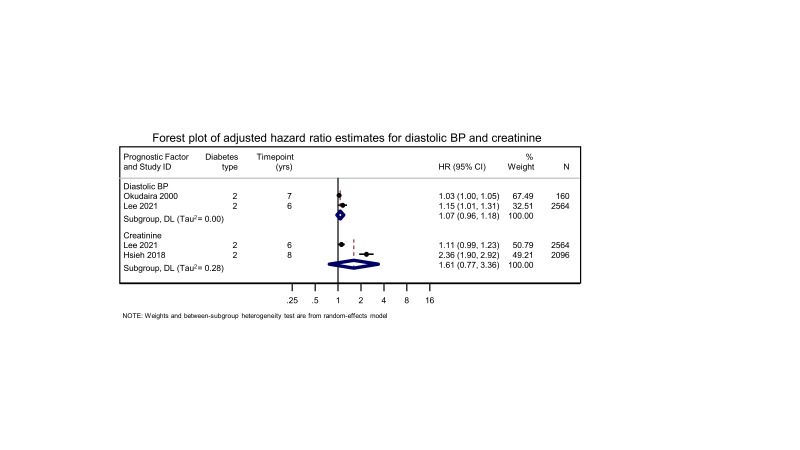
Forest plot of adjusted hazard ratio estimates for diastolic blood pressure and creatinine

Other studies undertook univariable regression analysis only. The Styles 2000 study included 52 participants with T1D followed for up to 45 years. According to the study publication, they did not find a "statistically significant" difference between median creatinine values in progressors (median 89; range 63 to 432) and non‐progressors to PDR (median value 78; range 56 to 330; P = 0.15) (Styles 2000).

In the only study on a mixed population of people with T1D and T2D, in univariable regression analysis and analysis of variance, the risk ratio for creatinine values of 133 mmol/L or higher (RR 1.04, 95% CI 0.88 to 1.24), and the mean difference between progressors and non‐progressors to PDR (mean difference 1.2, 95% CI ‐4.16 to 1.76; P = non‐significant) were not significant (Janghorbani 2000).

#### Ocular factors

##### DR severity at baseline

Twelve studies evaluated the effect of DR severity at baseline on progression to PDR (Arfken 1998; Burgess 2015; Klein 1984; Lee 2017; Lee 2021; Lloyd 1995; McCarty 2003; Nielsen 1984; Porta 2001; Roy 2006; Silva 2015; WESDR: Klein 1989a, Klein 1989b, 1994b, 1998, 2008). It was not possible to conduct a meta‐analysis, however, due to their heterogeneity. Most graded DR status based on the modified Airlie House classification system (Diabetic Retinopathy Study Research Group 1991) (Arfken 1998; Klein 1984; Lee 2017; Lloyd 1995; Porta 2001; Roy 2006; WESDR). The Nielsen 1984 study devised a scheme with four grades of background retinopathy, based on presence and number of red dots, haemorrhages and/or hard exudates, cotton wool spots, maculopathy and/or vitreous haemorrhage, and four grades of PDR, classified based on location of new vessels and presence of fibrovascular membranes, recurrent vitreous haemorrhage, neovascular glaucoma and/or retinal detachment. The Lee 2021 study categorised the level of DR according to the International Clinical Diabetic Retinopathy Disease Severity Scale (Wong 2018).

Six studies undertook multivariable regression (Arfken 1998; Lee 2017; Lee 2021; Lloyd 1995; Porta 2001; WESDR: Klein 1998, 2008) (Table 25). All found DR severity at baseline to be an independent predictor of PDR, with higher DR severity increasing the risk of PDR. We assessed the certainty of the evidence as moderate, suggesting DR severity at baseline is likely to be associated with risk of progression to PDR (Table 24).

Some studies undertook univariable analyses. In the WESDR study on T1D, in participants with moderate NPDR or worse in at least one eye at baseline, the risk of developing PDR was 42.4%, 63.6%, and 67.7% at four, 10 and 14 years, respectively (WESDR: Klein 1989b, 1994b, 1998). In the Roy 2006 study, in participants with moderate NPDR, 54.2% progressed to PDR during the six‐year follow‐up period. Porta 2001 reported that 17%, 40%, and 79% of participants with minimal, moderate, and severe NPDR, respectively, developed PDR during the seven‐year study duration. However, except for the Lloyd 1995 study, which accounted for HbA1c level, no other risk factors were included in the models (Porta 2001; Roy 2006; WESDR: Klein 1989b, 1994b, 1998)).

In T2D, the WESDR study found that, after four years of follow‐up, no participants in the older‐onset group taking insulin and with DR severity of less than level 21/21 (microaneurysms only or retinal haemorrhages or soft exudates in absence of microaneurysms) developed PDR. With increasing DR severity, progression to PDR generally increased. In the older‐onset group not taking insulin, progression to PDR increased significantly in participants with advancing levels of severity from 31/31 (microaneurysms and one or more of the following: venous loops 31 µm or greater; questionable soft exudate, intraretinal microvascular abnormalities (IRMA) or venous beading; and retinal haemorrhages) onwards (WESDR: Klein 1989a). At ten years' follow‐up, the trend in progression to PDR with increasing DR severity at baseline was "statistically significant" in both groups (using and not using insulin) (P < 0.001), according to the publication. In those with moderate NPDR or worse in at least one eye at baseline, 61.8% in the group diagnosed with DM at 30 years or older taking insulin and 50% in the group not taking insulin developed PDR (WESDR: Klein 1994b). The primary prognostic factor of interest in the Silva 2015 study, including 100 participants with T1D or T2D followed for four years, was the effect of predominantly peripheral lesions on the development of PDR, but an increase in the proportion of eyes developing PDR was observed with advancing DR severity at baseline. The percentages of participants developing PDR with mild, moderate, severe, and very severe NPDR at baseline were 6.1%, 13.3%, 36.4%, and 100%, respectively (Silva 2015).

##### DR features

Four studies assessed the influence of the presence of individual features of DR on the subsequent progression to PDR: two studies in people with T1D (WESDR; Verdaguer 2009), and two studies with a mixed population of people with T1D and T2D (Lee 2017; WESDR: Klein 1995c). Meta‐analysis was not possible due to their heterogeneity. 

Only two studies undertook multivariable regression analyses (Lee 2017; WESDR: Klein 1995c) (Table 26). To determine the effect of DR features on the incidence of PDR, Lee 2017 conducted a sub‐analysis of eyes with severe NPDR (n = 2823). The sub‐analyses included a total of 715 eyes, 240 eyes, and 169 eyes with IRMA, venous beading, and dot/blot haemorrhages in four quadrants, respectively. Lee 2017 established that the percentages of progressors to PDR by one, three, and five years were elevated in participants with IRMA (10.5%, 31.7%, 49.0%, respectively), followed by dot/blot haemorrhages (5.9%, 34.7%, 40.8%) in four quadrants and venous beading (5.0%, 17.2%, 39.9%). In multivariable Cox regression analysis, IRMA, but not dot/blot haemorrhages in four quadrants, was "statistically significantly" associated with increased risk of developing PDR compared to those with venous beading in two quadrants, according to the publication (Lee 2017) (Table 24).

A report by the WESDR study in a mixed population of people with T1D or T2D found that the difference and ratio in the number of microaneurysms in the worst affected eye between baseline and the four‐year follow‐up were "statistically significantly" associated with incidence of PDR, as quoted in the report (WESDR: Klein 1995c). For an increase of one retinal microaneurysm at the four‐year follow‐up, there was a 4% increased risk of developing PDR (WESDR: Klein 1995).

We rated the certainty of the evidence on the effect of DR features on development of PDR based on multivariable regression analyses as very low due to risk of bias, inconsistency, and imprecision (Table 24). 

The remaining two studies undertook univariable analyses only (Klein 1984; Verdaguer 2009). The Klein 1984 study (in people with T1D, follow‐up of six years), including an undetermined number of participants with moderate NPDR at baseline, evaluated the effect of hard exudates, cotton wool spots, IRMA, venous beading, and haemorrhages/microaneurysms (equalling or exceeding those in Standard Photo #3 as depicted by the DR Study Research Group; Diabetic Retinopathy Study Research Group 1981b) on the subsequent risk of PDR. The proportion of participants who progressed was identical in those with or without hard exudates (present 8/18; absent 11/25, 44%), and marginally increased in those with cotton wool spots (present 17/34, 50%; absent 2/9, 22%) and IRMA (present 11/18, 61%; absent 8/25, 32%) when compared with those without these features. There were too few with venous beading (n = 3) and haemorrhages/microaneurysms (n = 1) for an evaluation of risk in relation to these features (Klein 1984). The Verdaguer 2009 study (in 39 participants with T1D, follow‐up of 18 years) found in univariable analysis that hard exudates conferred a protective effect on the risk of PDR (OR 0.13, 95% CI 0.02 to 0.71; P = 0.011) (Verdaguer 2009).

The WESDR study examined the correlation between microaneurysms and progression to PDR, in 236 participants who had only microaneurysms at baseline, over four (WESDR: Klein 1989d) and 10 (WESDR: Klein 1995c) years. At four years, the ratio of total number of retinal microaneurysms at follow‐up divided by the number present at baseline was associated with the development of PDR (P < 0.001). All eyes that developed PDR had a ratio of three or more (WESDR: Klein 1989d). Similarly, at 10 years, progression to PDR was more common in eyes with three or more microaneurysms at baseline (P < 0.05) (WESDR: Klein 1995c).

##### PDR in fellow eyes

The Valone 1981 study included 136 participants with T2D (136 eyes) with NPDR in one eye and PDR in the fellow eye, and followed them up for at least three months (average follow‐up 34.5 months, 95% CI 29.4 to 39.6 months). They found that PDR developed in 58% (n = 73) of participants after an average follow‐up of 23.9 months (95% CI 18.8 to 29.0 months). A "statistically significantly" higher percentage of participants younger than 40 years of age (67%) compared to those older than 60 years (36%) developed PDR, according to the publication, but the analysis did not correct for other risk factors (Valone 1981).

In the Vesteinsdottir 2010 study, in a mixed population of people with T1D and T2D, 28 out of 48 (58%) developed PDR in the second eye within five years of its diagnosis in the first eye. Kaplan‐Meier analysis revealed that participants with T1D were at higher risk of developing PDR simultaneously in both eyes and within a short period when compared to those with T2D, but univariable analysis only was undertaken (Vesteinsdottir 2010).  

##### Retinal vessel caliber

Only two studies evaluated the effect of retinal vessel caliber on progression to PDR in participants with T1D (Roy 2006; WESDR: Klein 2004, 2007) (Table 27).

Roy 2006 found that increased central retinal vein equivalent (CRVE), defined as the average diameter of retinal venules measured at close proximity to the optic nerve head (but not central retinal artery equivalent, CRAE), was an independent predictor of the six‐year progression to PDR (P = 0.03) in univariable and multivariable models adjusted for HbA1c, age, sex, socioeconomic status, BMI, proteinuria, CRAE, ocular perfusion pressure, and refractive error (P = 0.03), with wider vein caliber found to increase the risk of PDR development (Roy 2006). DR severity at baseline was not included in these models.  

In the WESDR study, larger CRVE and smaller arteriolar‐venular ratio (AVR), but not CRAE, were statistically significantly correlated with greater four‐, 10‐, and 14‐year incidence of PDR (Klein 2004). In multivariable analysis (n = 871), larger venular diameters, but not arteriolar diameters, were associated with increased four‐year incidence of PDR (RR 4.28, 95% CI 1.50 to 12.19; P = 0.006), when controlling for sex, duration of diabetes, HbA1c, mean arterial blood pressure, antihypertension medication use, and DR severity at baseline. PDR was four times more likely to develop at four years in participants in whom the CRVE was in the fourth quartile range at baseline compared with participants in the first quartile range (WESDR: Klein 2004). 

In the T2D population, the WESDR study found larger CRVE ‐ defined as the CRVE in eyes in the fourth quartile compared with those in all other quartiles ‐ was associated with an increased 10‐year cumulative incidence of progression to PDR (P < 0.004). However, this relationship did not remain statistically significant in multivariate analyses (n = 889) controlling for age, HbA1c, and DR severity at baseline (P = 0.57). CRAE had no influence on the 10‐year development of PDR in univariate (P = 0.11) or multivariate (P = 0.78) analyses (WESDR: Klein 2007). 

We rated the overall certainty of the evidence for the effect of retinal vessel caliber as low, but the evidence suggests larger central retinal venular diameter may be associated with increased risk of progression to PDR in T1D (Table 24).

##### Intraocular pressure (IOP)

Only two studies evaluated the potential effect of IOP on incidence of PDR (Valone 1981; WESDR: Moss 1994), and both undertook multivariable regression analyses (Table 28). IOP was not found to be an independent predictor of incidence of PDR in either study. However, we rated the evidence as very uncertain due to risk of bias, inconsistency, and imprecision (Table 24).

Valone 1981 also compared the IOP in 44 participants with T2D evaluated over three years, and found no significant difference in the percentage of participants with higher IOP who remained with NPDR (53.8%) compared to those who progressed to PDR (61.3%) during the study (P > 0.05). 

#### Lifestyle Factors

##### Body mass index (BMI)

Fourteen studies evaluated the effect of BMI on progression to PDR: four in people with T1D (Grauslund 2009; Porta 2001; Styles 2000; WESDR: Klein 2008); nine studies in people with T2D (Gui 2013; Kim 1998; Kim 2014; Lee 1992; Lee 2021; Nelson 1989; Okudaira 2000; WESDR: Klein 1997; Zavrelova 2011); and two in a mixed population of participants with T1D or T2D (Janghorbani 2000; Keen 2001). 

Six studies undertook multivariable regression (Grauslund 2009; Keen 2001; Kim 1998; Lee 2021; Nelson 1989; WESDR: Klein 1997, 2008) (Table 30). Only the WESDR study, reporting outcomes at 25 years in participants with T1D, found BMI (per increase of 4 kg/m^2^) to be an independent predictor for the development of PDR, but DR severity at baseline was not accounted for in the model (WESDR: Klein 2008). BMI was not found to be an independent predictor of PDR in the other studies. However, we rated the evidence based on multivariable regression analyses to be very uncertain due to risk of bias, inconsistency, and imprecision (Table 29). 

It was only possible to undertake meta‐analysis of unadjusted effect estimates combining three studies (Kim 2014; Lee 2021; Okudaira 2000). The pooled HR was 0.99 (95% CI 0.96 to 1.03), which was consistent with the findings from multivariable regression analyses which also did not find BMI to be predictive of PDR. The prediction interval ranged from 0.78 to 1.26 (Figure 4).

All other studies, which undertook univariable analyses only, did not find BMI to be statistically significantly associated with progression to PDR (Gui 2013; Janghorbani 2000; Kim 2014; Lee 1992; Okudaira 2000; Porta 2001; Styles 2000; Zavrelova 2011).

##### Smoking

Thirteen studies evaluated the relationship between smoking and development of PDR: four studies in people with T1D (Grauslund 2009; Porta 2001; Styles 2000; WESDR: Moss 1991, 1996, Reports XVII, XXII); nine studies in people with T2D (Gange 2021; Gui 2013; Kim 2014; Lee 1992; Nelson 1989; Okudaira 2000; WESDR: Moss 1991, 1996; Zavrelova 2011); and two studies in a mixed population of people with T1D or T2D (Janghorbani 2000; Keen 2001). Meta‐analysis was not possible due to study heterogeneity. 

Six studies undertook multivariable regression (Gange 2021; Grauslund 2009; Gui 2013; Keen 2001; Nelson 1989; WESDR: Moss 1991, 1996, Report XVII) (Table 31). In all but one (Gui 2013), smoking was not found to be an independent predictor of development of PDR. Two studies including higher numbers of participants found smoking to have a protective effect in preventing PDR but neither included DR severity at baseline in their models (Gange 2021; Keen 2001). However, we rated the evidence based on multivariable regression analyses to be very uncertain due to risk of bias, inconsistency, and imprecision (Table 29).

The remaining studies undertook only univariable analyses. In people with T1D, some studies compared the smoking status in “progressors” and “non progressors” to PDR but did not find a significant difference (Porta 2001; Styles 2000). In a mixed population (n = 3468) of people with T1D and T2D with follow‐up of 5 years, Janghorbani 2000 found a negative association between current smoking and incidence of PDR (RR 0.68, 95%CI 0.55 to 0.84; P < 0.001), but a positive one between ex‐smokers and PDR (RR 1.36, 95% CI 1.14 to 1.62; P < 0.001) in univariable analysis. However, according to the publication, there was no "statistically significant" mean difference in the percentage of participants progressing to PDR in the non‐, ex‐, and current smoker groups (Janghorbani 2000). 

##### Alcohol

Only two studies considered the influence of alcohol consumption on the development of PDR (Styles 2000; WESDR: Moss 1994a). Of these, only the WESDR study undertook multivariable regression analysis. The study found that alcohol consumption was not an independent predictor for progression to PDR in the total population, or in male and female subgroups in either the younger‐onset or older‐onset groups (WESDR: Moss 1994a) (Table 32) (Table 29).

In the small Styles 2000 study (n = 52) in people with T1D followed for 45 years, there was no "statistically significant" difference between units of alcohol consumed per week by progressors to PDR compared to non‐progressors, as quoted in the publication (P = 0.31).

##### History of cardiovascular disease

Three studies explored the influence of a medical history of cardiovascular disease on progression to PDR (Jeng 2016; Lee 2021; Porta 2001). None found it to be a prognostic factor for PDR. Two of these studies undertook multivariable regression analyses (Jeng 2016; Lee 2021), but meta‐analysis was not possible due to study heterogeneity (Jeng 2016; Lee 2021; Porta 2001). 

#### Other prognostic factors 

A selection of other, less commonly evaluated, prognostic factors were investigated in individual studies, including: physical activity (WESDR: Cruickshanks 1995); myopia and ocular perfusion pressure (WESDR: Moss 1994a); alanine aminotransferase, haemoglobin, white blood cells, and platelets (Lee 2021); peripheral circulatory disorders (Gange 2021); peripheral lesions (Silva 2015); absence of Achilles tendon reflexes (Nelson 1989); cataracts (Verdaguer 2009); prothrombin time (Gui 2013); and oscillatory potential (Simonsen 1980). 

The WESDR study found no association between physical activity and development of PDR, even for those with more severe DR at baseline (WESDR:Cruickshanks 1995).

In the report on ocular factors and progression of PDR in the WESDR study, in multiple logistic regression analyses controlling for HbA1c, DR severity, and age at baseline in the younger‐onset group, myopia (≤ 2.00 dioptres) was found to be protective (OR 0.40, 95% CI 0.18 to 0.86), but not in the older‐onset group when controlling for HbA1c, duration of DM, and DR severity at baseline (OR 0.40, 95% CI 0.04 to 4.16) (WESDR: Moss 1994b). The study also investigated ocular perfusion pressure (mmHg), which was calculated from IOP, and blood pressure; these were not found to be significantly associated with progression to PDR in the younger‐onset (OR 1.21, 95% CI 0.76 to 1.94) or older‐onset, insulin‐taking (OR 1.04, 95% CI 0.64 to 1.71) groups (WESDR: Moss 1994b).

In a Cox proportional hazard model, adjusting for HbA1c, DR severity at baseline, gender, age, and BMI, Lee 2021 assessed the effect of alanine aminotransferase (U/L), haemoglobin (g/dL), white blood cells (10^3^/µL), and platelets (10^3^/µL) in 2623 participants with T2D at six years of follow‐up. Only haemoglobin was found to be an independent predictor of PDR (HR 0.84, 95% CI 0.74 to 0.96; P = 0.008) (Lee 2021).

In multivariable regression analysis, including HbA1c, age, sex, ethnicity, education level, income, smoking, hypertension, dyslipidaemia, renal and neurological disease, morbid obesity, and insulin use as covariates, Gange 2021 found that peripheral circulatory disorders were associated with development of PDR (OR 1.88, 95% CI 1.25 to 2.83; P = 0.003) whereas diabetic ketoacidosis was not (OR not reported, P > 0.05) (Gange 2021).

Silva 2015 found that peripheral lesions (haemorrhages, venous beading, IRMA, and NVE) identified on ultrawide field imaging were not "statistically significantly" associated with development of PDR, after adjusting for the previous two years’ HbA1c levels, DR severity at baseline, diabetes duration, and diabetes type in 109 participants with T1D or T2D followed for approximately four years (Silva 2015).

In the Nelson 1989 study on Pima Indians with T2D aged 35 years or older, the absence of Achilles tendon reflexes was associated with PDR (incidence‐rate ratio 4.4, 95% CI 1.3 to 14.9) in a Cox’s proportional hazard model controlling for age, sex, and diabetes duration (Nelson 1989).

In a small but long‐term study of 39 participants followed for 18 years, Verdaguer 2009 compared the presence of cataracts in groups with NPDR and PDR but did not find a significant difference (P = 0.117).

Gui 2013 found that the mean difference in prothrombin time between non‐progressors (10.6, SE 2.11) and progressors (18.4, SE 3.05) to PDR was not significant (P = 0.21) in 190 participants with T2D followed for approximately two years.

The Simonsen 1980 study explored the value of oscillatory potentials in detecting participants with T1D at risk of developing PDR. A total of 137 participants were followed at six to eight and 13 to 15 years. In all participants with PDR, oscillations were significantly reduced or extinguished (despite normal latencies of a‐ and b‐waves).

### Prognostic factors for progression to HRC‐PDR

Only three studies ‐ Roy 2006; Varma 2010; WESDR ‐ evaluated prognostic factors associated with progression specifically to HRC‐PDR, as defined by the DR Study Research (DRS) Group (and in our protocol) by the presence of NVD more than one‐fourth to one‐third disc area in size, or NVD/NVE of any size associated with vitreous or pre‐retinal haemorrhages (Diabetic Retinopathy Study Research Group 1991). None of these studies, however, looked at the specific risk of progression from PDR to HRC‐PDR. 

The WESDR study reported the effect of DR severity in T1D on development of HRC‐PDR at four, 10 and 14 years in participants with NPDR at baseline (WESDR: Klein 1989b, 1994b, 1998). Varma 2010 investigated the influence of age at baseline and duration of PDR on progression to HRC‐PDR, in participants with T1D and T2D (mixed cohort) over a four‐year period, but all had NPDR at baseline. The Roy 2006 study assessed the influence of gender and retinal vessel caliber on progression to HRC‐PDR, but some participants had no DR or NPDR at baseline. 

### Sensitivity analysis and sources of heterogeneity

There were insufficient data to explore the impact of studies at high risk of bias or retrospective studies on the effect sizes observed, as we had originally planned. For the same reason, we were unable to explore sources of heterogeneity between studies. 

## Discussion

### Summary of main results

This systematic review found HbA1c and DR severity at baseline to be independent predictors for the development of PDR in people with T1D or T2D, with higher levels of HbA1c and retinopathy increasing the risk of PDR. Included studies used different biomarkers indicative of renal disease (nephropathy, proteinuria, albumin excretion rate, ACR, eGFR, creatinine), with most pointing towards a possible increased risk of progression to PDR in people with impaired kidney function. Age at diagnosis of diabetes, elevated triglyceride levels, and larger retinal venular diameters may also possibly be associated with progression to PDR in people with T1D. There was no clear evidence that duration of diabetes had an influence on the development of PDR when HbA1c and DR severity were included in the models. Neither do SBP, DBP, total cholesterol, LDL or HDL levels, nor gender, ethnicity, BMI, socioeconomic status, smoking, and alcohol consumption appear to be associated with progression to PDR. 

Despite 59 studies (87 reports) being included in the review, the great heterogeneity in study design, prognostic factors evaluated, how they were measured, the lack of adjustment for potentially important risk factors in some studies and their consideration in statistical models, as well as weaknesses in the quality of reporting, significantly limited our ability to undertake meta‐analysis and even interpret with certainty their results (Figure 2). 

### Prognostic factors found to be associated with progression to PDR

HbA1c was identified as an independent predictor of PDR in almost all studies in which multivariable regression analyses were undertaken, with the exception of Cho 2019 and Lee 2021, both retrospective cohort studies including Asian populations with T2D (Cho 2019: n = 1527; Lee 2021: n = 2623). Besides differences in ethnicity when compared with the other studies, HbA1c levels at baseline in these studies appeared to be lower, which may at least partly explain these discrepant results (Cho 2019; Lee 2021).

The finding that elevated HbA1c contributes to the development of PDR, even when taking into consideration DR severity, challenges the hypothesis of 'retinopathic momentum', which suggests that once DR progresses far enough, no intervention will halt its relentless progression (Diabetes Control and Complications Group 1993). A review by Liu and colleagues similarly proposed that intensive glycaemic control may not be beneficial if DR severity is worse than moderate NPDR (Liu 2020). The fact that HbA1c levels are a risk factor for PDR is also supported by interventional RCTs, which found that a more rigorous glycaemic control has beneficial effects in reducing risk of progression of DR, albeit this being more pronounced in people with T1D when compared to those with T2D (ACCORD 2011; Diabetes Control and Complications Group 1993; Turner 1998). Furthermore, results of a meta‐analysis of 16 interventional RCTs concluded that intensive glycaemic control significantly delayed progression to PDR or requirement of laser treatment for PDR in people with T1D (OR 0.44, 95% CI 0.22 to 0.87; P = 0.018) (Wang 1993). It is important to note that rapid changes in glycaemia should be avoided, as these may lead to acceleration of the progression of DR, including development of PDR (Diabetes Control and Complications Group 1998). This aligns with the findings of a recent meta‐analysis of RCTs that the use of newer glucagon‐like peptide‐1 receptor agonists (GLP 1 RA) (e.g. liraglutide, semaglutide and dulaglutide) was associated with an increased risk of rapidly worsening of DR (Yoshida 2022).

DR severity at baseline was also found to be a consistent and significant independent predictor of PDR development. In this regard, the ETDRS found that severity of DR features at baseline was the most important factor predicting progression of DR (ETDRS 1991b). Interestingly, when DR severity was included in multivariable regression models in many studies included in our review, the effect of other risk factors, initially potentially linked to PDR, was no longer observed. This suggests that the retina portrays the effects of the systemic environment and, as a result, changes in the retina have more prognostic value than that of the risk factors themselves.

Renal impairment, determined by different means in the various studies included, was generally found to be an independent predictor of PDR. Thus, three out of four studies evaluating nephropathy using multivariable regression found this diabetic complication increased the risk of progression to PDR. Similarly, five of the eight studies evaluating proteinuria, two of the three studies using “albumin excretion rate” or “albumin creatinine ratio” or “creatinine”, and the two studies that used “eGFR” found, in multivariable regression models, that deranged kidney function was associated with increased risk of development of PDR. Heterogeneity in the populations studied and in the various definitions used for renal impairment may at least partly explain discrepancies in the results observed amongst different studies. Given that similar histopathological alterations are present in retina and kidney in people with diabetes ‐ including basement membrane thickening, endothelial cell dysfunction/loss, and loss of pericytes in the retina and their kidney counterpart (podocytes), amongst others ‐ it is not surprising that disease in both organs may be interrelated (Wong 2014). Indeed, the overall prevalence of PDR in a cohort of 15,409 participants over 19 years of age with T1D or T2D was found to be 5.5% if chronic kidney disease was present compared to 1.8% if it was not (Park 2015). A study of 1214 participants with T2D found a profound difference in the prevalence of PDR in those on dialysis (31.7%) compared to those not requiring it (1.9%) (P < 0.001) (Boelter 2016). 

Evidence on the relationship between triglycerides and PDR was relatively limited, but suggested that increased triglyceride levels in T1D, but not in T2D, may be associated with development of PDR. Other studies not included in this review have suggested a possible association between triglyceride levels and PDR. Thus, in a mixed population of 2651 participants with T1D or T2D, the ETDRS found that increased triglyceride levels were associated with progression to HRC‐PDR in multivariable models controlling for HbA1c, type and duration of DM, DR severity at baseline, age, gender, ethnicity, weight, visual acuity and presence of diabetic macular oedema (DMO) (Davis 1998). Moreover, in an Irish cohort study including 2770 participants with T2D, triglycerides were positively correlated with increased risk of referable DR (HR 1.10, 95% CI 1.03 to 1.18; P = 0.004) (Smith 2020). It should be noted, though, that 'referable DR' includes PDR but also higher stages of NPDR and DMO. However, the Diabetes Control and Complications Trial (DCCT), which included 1441 participants with T1D, did not find triglyceride levels to be an independent predictor of progression to PDR in multivariable regression controlling for HbA1c, duration of DM, DR severity at baseline, randomised treatment, age, sex, and smoking (Miljanovic 2004). Unlike cholesterol, levels of triglycerides are greatly influenced by whether or not blood samples obtained for their analysis are gathered following fasting. Thus, a fasting blood sample is required to ensure accuracy of results; these were obtained in some of the studies included in this review in which the potential relationship between triglyceride levels and PDR was tested, but not reported in others.

It is possible that age at diagnosis of DM may also determine risk of development of PDR. Although this conclusion is drawn from results of only two studies in which multivariable regression models were used, in one of these by Porta and colleagues, the effect of younger age at diagnosis was observed even when DR severity at baseline was included in the model, suggesting it is indeed an important risk factor, at least in people with T1D (Porta 2001). 

Similarly, in people with T1D, larger retinal venular diameter was also found to be associated with an increased risk of PDR in two studies (Roy 2006; WESDR: Klein 2004). In one of these, the effect remained after controlling in a multivariable regression model for DR severity at baseline (WESDR: Klein 2004). This finding is in accordance with studies evaluating pathogenic mechanisms of disease in DR. These showed that the arteriolar vasoconstriction observed in early DR stages with subsequent blood flow reduction is followed by vasodilation and enhanced blood flow which could then hasten the development of PDR (Stitt 2016).   

### Prognostic factors not found to be associated with progression to PDR

Duration of diabetes in people with T1D was only found to be an independent predictor of the development of PDR when DR severity at baseline was not included as a covariate in multivariable regression models (Grauslund 2009; Janghorbani 2000; Kalter‐Leibovici 1991; Lloyd 1995; Porta 2001). In T2D, results were inconsistent and none of the studies using multivariable regression models included DR severity at baseline. It may be challenging to determine the exact date of diagnosis of DM in people with T2D, and, thus, its relationship to PDR. In this regard, studies have suggested that glucose dysregulation can precede the diagnosis of T2D by up to 20 years (Sagesaka 2018). Similarly, in nearly all studies evaluating DBP, SBP, and total cholesterol, these were only independently associated with progression to PDR when DR severity at baseline was not included in multivariate regression models. This suggests that the influence of these factors may not be evident beyond their effect already imprinted in the retina.

Our findings with regard to the effect of blood pressure (BP) supported those of a Cochrane Review (Do 2015). It included 15 interventional RCTs involving participants with T1D and T2D, and did not find a beneficial effect in reducing blood pressure to prevent progression to PDR (estimated RRs 0.95, 95% CIs 0.83 to 1.09), although there was a benefit in delaying the incidence of DR (Do 2015). In the UK Prospective Diabetes Study (UKPDS), the beneficial effects of BP control were only seen when baseline BP was very high (160/94 mmHg) (UK Prospective Diabetes Study Group 1998). However, it is possible that in a real‐world clinical setting, individuals’ control of BP may be as poor as it was in the UKPDS. Under these circumstances, there would be an overall benefit of reducing levels of BP in order to reduce the risk of development of other diabetic complications. 

Aside from the possible effect of triglycerides in people with T1D, as reviewed above, dyslipidaemia ‐ although evaluated extensively in relation to incidence and progression of DR ‐ was only rarely studied with regard to its relationship with the incidence of PDR, limiting available data. 

In studies undertaking multivariable regression, BMI was not associated with progression to PDR, with the exception of the 25‐year prospective cohort study by Klein and colleagues (WESDR: Klein 2008), which found BMI (per 4 kg/m^2^) to be an independent predictor of the incidence of PDR in people with T1D. However, DR severity at baseline was not included in the model. Discordance in the relationship between obesity and PDR may be due to BMI being an inaccurate indicator of central fat distribution, which has been found to be associated with more severe stages of DR in those with T2D (Man 2016; Raman 2010). Indeed, in Parente and colleagues' 15‐year observational cohort study, waist‐to‐height ratio and waist circumference were more significant indicators of progression to "serious diabetic eye disease" than BMI (Parente 2022).

Our review did not find evidence for an effect of smoking on the risk of development of PDR. Although nicotine can cause vasoconstriction in normal circumstances, which would be expected to reduce blood flow through the retina, it is possible this effect may not alter the blood flow in the diabetic retina given that vasodilation of arteriolas is known to be part of the dysregulation present in the diabetic retina in more advanced stages of disease (Mills 2021; Schmetterer 1999).

### Strengths and weaknesses of the review

Strengths of this review include our comprehensive and systematic search of the literature for relevant studies, with no language or date restrictions, and our detailed scrutiny of all references listed in all included studies. We believe this rigorous search process means it is unlikely we missed relevant literature. We conducted the review according to Cochrane’s standards.  

The review's limitations relate to features of the included studies, such as the heterogeneity in study design, populations, follow‐up period, type of analyses undertaken, and adjustment (or lack thereof) for other potentially important risk factors. Thus, study heterogeneity greatly restricted our ability to undertake meta‐analysis. Furthermore, anomalies in the reporting of important baseline characteristics of study participants, such as the level of DR severity at baseline, ethnicity, and HbA1c and blood pressure levels, resulted in some uncertainty when interpreting outcomes of various studies in the context of the populations investigated. Moreover, some studies did not specify the direction of the effect of particular prognostic factors studied, making it impossible to interpret findings. Many studies undertook only univariable regression analyses when evaluating specific risk factors for progression to PDR, thus disregarding the effect of other potentially important factor. Based on our review, HbA1c and DR severity should always be corrected for in future PDR prognosis studies. Other potential limitations of the data presented relate to limitations inherent to the techniques used to diagnose PDR: most studies used standard fundus photography. Thus, it is very possible that cases of early PDR missed by standard fundus photography could have been identified by the use of fundus fluorescein angiography.

Substantial resources are required to conduct prognosis studies, not only with regard to costs, but also the time and effort invested by participants and researchers. Thus, it is crucial that future studies are rigorously planned, and appropriately statistically analysed and reported, to ensure maximal benefit to people with diabetic retinopathy is realised. Guidelines provided by the Transparent Reporting of a multivariable prediction model for Individual Prognosis Or Diagnosis (TRIPOD) initiative (Patzer 2021; www.tripod‐statement.org/scope), as well as those provided by the Quality In Prognosis Studies (QUIPS) tool (developed to assess risk of bias in prognosis studies), could help researchers to design more robust prognosis studies.

### Conclusion

Increased HbA1c is likely to be associated with progression to PDR, and therefore, maintaining adequate glucose control throughout life, irrespective of stage of DR severity, may help to prevent progression to PDR and risk of its sight‐threatening complications. Renal impairment in people with T1D or T2D, as well as younger age at diagnosis of DM, increased triglyceride levels, and retinal venular diameters in people with T1D may also be associated with increased risk of progression to PDR. Given that more advanced DR severity is associated with higher risk of progression to PDR, the earlier the disease is identified, and the above systemic risk factors are controlled, the greater the chance of reducing the risk of PDR and saving sight.

## Authors' conclusions

There is evidence to support that elevated glycated haemoglobin (HbA1c) and more advanced diabetic retinopathy (DR) severity at baseline are independent risk factors for the development of proliferative diabetic retinopathy (PDR) in people with type 1 (T1D) or type 2 (T2D) diabetes. Evidence for other risk factors is less compelling, although it suggests that renal disease in people with T1D and T2D, and younger age at diagnosis of diabetes, higher triglyceride levels, and larger retinal venular diameters in people with T1D may also increase the risk of PDR. 

Despite the large number of cohort studies that have been undertaken over the years, the great heterogeneity in study design, prognostic factors evaluated and how they were measured, the lack of adjustment for potentially important risk factors in some studies and their consideration in statistical models, as well as weaknesses in the quality of reporting, significantly limited our ability to interpret their results.  

### Implications for practice

Evidence from this review suggests it is likely that maintaining adequate glucose control throughout life reduces the risk of developing PDR. People with T1D or T2D and renal disease may be at increased risk of developing PDR. Maintaining triglyceride levels within the normal range may also reduce the risk of progression to PDR in people with T1D. Research has shown that fenofibrate, which lowers triglycerides, has other additional beneficial effects in the retina independent of the lipid reduction (Chew 2003; Keech 2007; Stewart 2018). However, at present, this drug is not licenced in the UK for the treatment of DR.

### Implications for research

More robust research is necessary to adequately determine prognostic factors associated with PDR and, specifically, progression from PDR to high‐risk characteristics DPD (HRC‐PDR) in order to identify individuals at higher risk of developing sight‐threatening complications who may benefit from even earlier interventions.

We identified HbA1c and DR severity at baseline as being the most significant risk factors for development of PDR. Thus, it is essential that future studies adjust for them in their prognostic models. 

The heterogeneity in study design, characteristics of populations included, follow‐up period, types of analyses undertaken, and adjustment (or lack thereof) for other potentially important prognostic factors greatly limited our ability to undertake meta‐analysis. Researchers should be mindful of this. Establishing a core outcome set for prognosis studies in the field of DR, as advocated by the Core Outcome Measures in Effectiveness Trials (COMET) initiative (Mokkink 2016), and homogenising the instruments to measure these outcomes, as proposed by the COnsensus‐based Standards for the selection of health Measurement Instruments (COSMIN) initiative (www.cosmin.nl), would greatly facilitate synthesis of data to derive more meaningful conclusions. We encourage these developments.

Frequently in this review, prognosis studies were not eligible for inclusion because study authors reported only a combined outcome of development of PDR or diabetic macular oedema (DMO; what is called 'sight‐threatening' DR), or generalised progression of two to three steps on the Early Treatment Diabetic Retinopathy Study (ETDRS) scale. Given that the risk of blindness conferred by each of these conditions is very different and that indeed total blindness (central and peripheral) still occurs in present times only as a result of complications of PDR, it is essential clinicians and researchers look into each of these outcomes separately in future studies. 

Lastly, establishing a database of prognosis studies in diabetes and DR would facilitate the identification of such studies in medical database searches and would thus aid and advance research efforts in this field. 

## History

Protocol first published: Issue 11, 2020

## Appendices

### Appendix 1. CENTRAL search strategy

#1 MeSH descriptor: [Risk Factors] this term only #2 risk factor* #3 MeSH descriptor: [Biomarkers] this term only #4 biomarker* #5 marker* #6 biological marker* #7 MeSH descriptor: [Vascular Endothelial Growth Factor A] this term only #8 Vascular Endothelial Growth Factor A #9 VEGF #10 MeSH descriptor: [Intercellular Signaling Peptides and Proteins] this term only #11 growth factor* #12 MeSH descriptor: [Erythropoietin] explode all trees #13 erythropoietin* #14 EPO #15 retinal angiogenic factor* #16 MeSH descriptor: [Epidemiology] explode all trees #17 epidemiolog* #18 potential role* #19 (risk* or rate*) NEAR/5 (progress* or complicat*) #20 MeSH descriptor: [Risk Assessment] this term only #21 risk* NEAR/5 (assess* or stratif*) #22 MeSH descriptor: [Phenotype] explode all trees #23 phenotype* #24 MeSH descriptor: [Prognosis] this term only #25 prognos* #26 predict* #27 model* #28 variable* #29 #1 or #2 or #3 or #4 or #5 or #6 or #7 or #8 or #9 or #10 or #11 or #12 or #13 or #14 or #15 or #16 or #17 or #18 or #19 or #20 or #21 or #22 or #23 or #24 or #25 or #26 or #27 or #28 #30 MeSH descriptor: [Diabetic Retinopathy] this term only #31 proliferative diabetic retinopathy* #32 PDR #33 non?proliferative diabetic retinopathy* #34 NPDR #35 complication* adj5 (diabetic retinopathy* or DR) #36 microvascular complication* NEAR/5 diabet* #37 severity* NEAR/5 (diabetic retinopathy* or DR) #38 advanced NEAR/5 (diabetic retinopathy* or DR*) #39 severe retinopathy* #40 MeSH descriptor: [Retinal Neovascularization] this term only #41 new vessel* #42 retina* NEAR/5 neo?vasculari* #43 (neovasculari* or new vessel*) NEAR/5 (disc* or retina* or elsewhere or iris*) #44 NVD or NVE or NVI #45 rubeosis iridis* #46 (vision* or sight*) NEAR/5 threat* adj5 (diabet* or retinopathy*) #47 #30 or #31 or #32 or #33 or #34 or #35 or #36 or #37 or #38 or #39 or #40 or #41 or #42 or #43 or #44 or #45 or #46 #48 MeSH descriptor: [Vitreous Hemorrhage] this term only #49 vitreous h?emorrhage* #50 fibro?proliferative disease* #51 tractional retinal detachment* #52 rhegmatogenous retinal detachment* #53 MeSH descriptor: [Glaucoma, Neovascular] this term only #54 neovascular glaucoma* #55 NVG #56 (moderate* or severe* or reduced) NEAR/5 vis* #57 MeSH descriptor: [Blindness] this term only #58 registered NEAR/5 blind #59 blindness* #60 partial* sight* #61 #48 or #49 or #50 or #51 or #52 or #53 or #54 or #55 or #56 or #57 or #58 or #59 or #60 #62 occurrence* #63 advancement* #64 worsen* #65 evolution* or evolv* #66 relationship* between #67 MeSH descriptor: [Association] this term only #68 MeSH descriptor: [Correlation of Data] this term only #69 MeSH descriptor: [Incidence] this term only #70 MeSH descriptor: [Prevalence] this term only #71 MeSH descriptor: [Disease Progression] explode all trees #72 natural histor* #73 natural course* #74 #62 or #63 or #64 or #65 or #66 or #67 or #68 or #69 or #70 or #71 or #72 or #73 #75 29 and 47 and 61 and 74

### Appendix 2. MEDLINE search strategy

1. Risk Factors/ 2. risk factor*.tw. 3. Biomarkers/ 4. biomarker*.tw. 5. marker*.tw. 6. biological marker*.tw. 7. Vascular Endothelial Growth Factor A/ 8. Vascular Endothelial Growth Factor A.tw. 9. VEGF.tw. 10. "Intercellular Signaling Peptides and Proteins"/ 11. growth factor*.tw. 12. exp Erythropoietin/ 13. erythropoietin*.tw. 14. EPO.tw. 15. retinal angiogenic factor*.tw. 16. exp Epidemiology/ 17. epidemiolog*.tw. 18. potential role*.tw. 19. ((risk* or rate*) adj5 (progress* or complicat*)).tw. 20. Risk Assessment/ 21. (risk* adj5 (assess* or stratif*)).tw. 22. exp Phenotype/ 23. phenotype*.tw. 24. Prognosis/ 25. prognos*.tw. 26. predict*.tw. 27. model*.tw. 28. variable*.tw. 29. or/1‐28 30. Diabetic Retinopathy/ 31. proliferative diabetic retinopathy*.tw. 32. PDR.tw. 33. non?proliferative diabetic retinopathy*.tw. 34. NPDR.tw. 35. (complication* adj5 (diabetic retinopathy* or DR)).tw. 36. (microvascular complication* adj5 diabet*).tw. 37. (severity* adj5 (diabetic retinopathy* or DR)).tw. 38. (advanced adj5 (diabetic retinopathy* or DR*)).tw. 39. severe retinopathy*.tw. 40. Retinal Neovascularization/ 41. (retina* adj5 neo?vasculari*).tw. 42. new vessel*.tw. 43. ((neovasculari* or new vessel*) adj5 (disc* or retina* or elsewhere or iris*)).tw. 44. (NVD or NVE or NVI).tw. 45. rubeosis iridis*.tw. 46. ((vision* or sight*) adj5 threat* adj25 (diabet* or retinopathy*)).tw. 47. or/30‐46 48. Vitreous Hemorrhage/ 49. vitreous h?emorrhage*.tw. 50. fibro?proliferative disease*.tw. 51. tractional retinal detachment*.tw. 52. rhegmatogenous retinal detachment*.tw. 53. Glaucoma, Neovascular/ 54. neovascular glaucoma*.tw. 55. NVG.tw. 56. ((moderate* or severe* or reduced) adj5 vis*).tw. 57. Blindness/ 58. (registered adj5 blind).tw. 59. blindness*.tw. 60. partial* sight*.tw. 61. or/48‐60 62. occurrence*.tw. 63. advancement*.tw. 64. worsen*.tw. 65. (evolution* or evolv*).tw. 66. relationship* between.tw. 67. Association/ 68. "correlation of data"/ 69. incidence/ or prevalence/ 70. exp disease progression/ 71. natural histor*.tw. 72. natural course*.tw. 73. or/62‐72 74. 29 and 47 and 61 and 73

### Appendix 3. Embase search strategy

1. risk factor/ 2. risk factor*.tw. 3. exp marker/ 4. biomarker*.tw. 5. marker*.tw. 6. vasculotropin/ 7. Vascular Endothelial Growth Factor A.tw. 8. VEGF.tw. 9. growth factor/ 10. growth factor*.tw. 11. erythropoietin/ 12. erythropoietin*.tw. 13. EPO.tw. 14. retinal angiogenic factor*.tw. 15. exp epidemiology/ 16. epidemiolog*.tw. 17. potential role*.tw. 18. ((risk* or rate*) adj5 (progress* or complicat*)).tw. 19. risk assessment/ 20. exp phenotype/ 21. phenotype*.tw. 22. prognosis/ 23. prognos*.tw. 24. predict*.tw. 25. model*.tw. 26. variable*.tw. 27. inter?cellular signal*.tw. 28. or/1‐27 29. diabetic retinopathy/ or proliferative diabetic retinopathy/ 30. proliferative diabetic retinopathy*.tw. 31. PDR.tw. 32. non?proliferative diabetic retinopathy.tw. 33. NPDR.tw. 34. (complication* adj5 (diabetic retinopathy* or DR)).tw. 35. (microvascular complication* adj5 diabet*).tw. 36. (severity* adj5 (diabetic retinopathy* or DR)).tw. 37. (advanced adj5 (diabetic retinopathy* or DR)).tw. 38. severe retinopathy*.tw. 39. retina neovascularization/ 40. (retina* adj5 neovasculari*).tw. 41. new vessel*.tw. 42. (neovasculari* adj5 (disc* or retina* or elsewhere or iris*)).tw. 43. (NVD or NVE or NVI).tw. 44. iris rubeosis/ 45. rubeosis iridis*.tw. 46. ((vision* or sight*) adj5 threat* adj5 (diabet* or retinopathy*)).tw. 47. or/29‐46 48. vitreous hemorrhage/ 49. vitreous h?emorrhage*.tw. 50. fibro?proliferative disease*.tw. 51. tractional retinal detachment*.tw. 52. rhegmatogenous retinal detachment*.tw. 53. neovascular glaucoma/ 54. neovascular glaucoma*.tw. 55. NVG.tw. 56. ((moderate* or severe* or reduced) adj5 vis*).tw. 57. blindness/ 58. (registered adj5 blind).tw. 59. blindness.tw. 60. partial* sight*.tw. 61. or/48‐60 62. occurrence*.tw. 63. advancement*.tw. 64. worsen*.tw. 65. (evolution* or evolv*).tw. 66. relationship* between.tw. 67. association/ 68. data correlation/ 69. incidence/ 70. prevalence/ 71. disease exacerbation/ 72. natural histor*.tw. 73. disease course/ 74. natural course*.tw. 75. or/62‐74 76. 28 and 47 and 61 and 75

### Appendix 4. CHARMS‐PF data extraction

**Table:**

	**Study**
**Study design**	Source of data (e.g. cohort, case‐control, randomised trial, or registry data)
	Dates
**Participants**	Participant eligibility and recruitment method (e.g. consecutive participants, location, number of centres, setting, inclusion and exclusion criteria)
	Participant description
	Details of treatment received, if relevant
**Outcomes to be predicted**	Definition of outcome
	Method of measurement
	Time of outcome occurrence
**Prognostic factors (index and comparator)**	Type of prognostic factors
	Definition and method of measurement for prognostic factors
	Timing of prognostic factor measurement
	Handling of prognostic factors in the analysis
**Sample size**	Was a sample size calculation conducted, and if so, how?
	Number of participants
	Number of outcomes
	Number of outcomes in relation to number of candidate prognostic factors (outcomes per variable)
**Missing data**	Number of participants with missing data for each prognostic factor of interest
	Details of attrition and, for time‐to‐event outcomes, number of censored observations
	Handling of missing data
**Analysis**	Modelling method of analysis
	How modelling assumptions were checked: in particular, for time‐to‐event outcomes and the analysis of hazard ratios, the method for assessing non‐proportional hazards (non‐constant hazard ratios over time)
	Method for selection of prognostic factors for inclusion in multivariable modelling (e.g. all candidate prognostic factors considered, preselection of established prognostic factors, retain only those significant from univariable analysis)
	Method for selection or exclusion of prognostic factors (including those of interest and those used as adjustment factors) during multivariable modelling (e.g. backward or forward selection, or full model approach including all factors regardless) and criteria used for any selection or exclusion (e.g. P value, Akaike information criterion)
**Results**	Unadjusted and adjusted prognostic effect estimates (e.g. risk ratios, odds ratios, hazard ratios, mean differences) for each prognostic factor of interest, and the corresponding 95% confidence interval (or variance or standard error)
	For each extracted adjusted prognostic effect estimate of interest, the set of adjustment factors used

### Appendix 5. Quality in Prognosis Studies (QUIPS) tool

**Table:**

**Domains**	**Signalling items**	**Risk of bias ratings**
**1. Study participation**	(a) Adequate participation in study by eligible individuals	Relationship between PF and outcome ‐
	(b) Description of target population	High: **very likely** to be different for participants and eligible non‐participants
	(c) Description of baseline study sample	Moderate: **may** be different for participants and eligible non‐participants
	(d) Adequate description of recruitment process	Low: **unlikely** to be different for participants and eligible non‐participants
	(e) Adequate description of period and place of recruitment	
	(f) Adequate description of inclusion/exclusion criteria	
**2. Study attrition**	(a) Adequate response rate for study participants	Relationship between PF and outcome ‐
	(b) Description of process for collecting information on participants who dropped out	High: **very likely** to be different for completing and non‐completing participants
	(c) Reasons for loss to follow‐up provided	Moderate: **may** be different for completing and non‐completing participants
	(d) Adequate description of participants lost to follow‐up	Low: **unlikely** be different for completing and non‐completing participants
	(e) No important differences between participants who completed the study and those who dropped out	
**3. Prognostic factor (PF) measurement**	(a) Clear definition of PF provided	Measurement of PF ‐
	(b) Method of PF measurement is adequately valid and reliable	High: **very likely** to be different for different levels of outcome of interest
	(c) Continuous variables are reported	Moderate: **may** be different for different levels of outcome of interest
	(d) Method and setting of measurement of PF is identical for all participants	Low: **unlikely** to be different for different levels of outcome of interest
	(e) Adequate proportion of study sample has complete data for PF	
	(f) Appropriate methods of imputation used for missing PF data	
**4. Outcome measurement**	(a) Clear definition of outcome provided	High: outcome measurement **very likely** to be different related to baseline level of PF
	(b) Method of outcome measurement is adequately valid and reliable	Moderate: outcome measurement **may** be different related to baseline level of PF
	(c) Method and setting of outcome measurement is identical for all participants	Low: outcome measurement **unlikely** to be different related to baseline level of PF
**5. Adjustment for other prognostic factors**	(a) All other important PFs measured	Observed effect of PF on outcome ‐
	(b) Clear definitions of important PFs measured provided	High: **very likely** to be distorted by another factor related to PF and outcome
	(c) Measurement of all important PFs adequately valid and reliable	Moderate: **may** be distorted by another factor related to PF and outcome
	(d) Measurement and setting of PF measurement identical for all participants	Low: **unlikely** to be distorted by another factor related to PF and outcome
	(e) Appropriate methods are used to deal with missing values of PFs	
	(f) Important PFs accounted for in study design	
	(g) Important PFs accounted for in analysis	
**6. Statistical analysis and reporting**	(a) Sufficient presentation of data to assess adequacy of analytic strategy	Reported results ‐
	(b) Strategy for model building appropriate and based on a conceptual framework or model	High: **very likely** to be spurious or biased related to analysis or reporting
	(c) Selected statistical model adequate for design of study	Moderate: **may** be spurious or biased related to analysis or reporting
	(d) No selective reporting of results	Low: **unlikely** to be spurious or biased related to analysis or reporting

**PF:** prognostic factor

### Appendix 6. QUIPS ‐ authors' judgements for low risk of bias

**Table:**

**Domain**	**Signalling items**	**Authors' judgement for 'yes'**
1. Study participation	(a) Adequate participation in study by eligible individuals	The sampling frame and recruitment are adequately described, including methods to identify the sample sufficient to limit potential bias (number and type used, e.g. referral patterns in health care)
	(b) Description of target population	Source population for cohort with diabetic retinopathy (DR) is clearly described
	(c) Description of baseline study sample	Number of people with DR at baseline is clearly described
	(d) Adequate description of recruitment process	Way of establishing the source population, selection criteria and key characteristics of the source population clearly described
	(e) Adequate description of period and place of recruitment	Time period and place of recruitment for both baseline and follow‐up examinations are clearly described
	(f) Adequate description of inclusion/exclusion criteria	Definition of DR and other inclusion and exclusion criteria clearly defined
	**Domain overall risk of bias **	**High:** most items are answered with 'no'**; Low:** all items answered with 'yes'**; Moderate:** most items are answered with 'unclear'
2. Study attrition	(a) Adequate response rate for study participants	Response rate (i.e., proportion of study sample completing the study and providing outcome data) is adequate
	(b) Description of process for collecting information on participants who dropped out	Attempts to collect information on participants who dropped out are described (e.g. telephone contact, mail, registers)
	(c) Reasons for loss to follow‐up provided	Reasons on participants who dropped out are reported
	(d) Adequate description of participants lost to follow‐up	Key characteristics of participants lost to follow‐up are described
	(e) No important differences between participants who completed the study and those who dropped out	Study authors described differences between participants completing the study and those who did not as not important or information provided to judge the differences
	**Domain overall risk of bias**	**High:** most items are answered with 'no'**; Low:** all items answered with 'yes'**; Moderate:** most items are answered with 'unclear'
3. Prognostic factor measurement	(a) Clear definition of prognostic factor (PF) provided	Measurements for prognostic factors (PFs) are provided
	(b) Method of PF measurement is adequately valid and reliable	Measurements techniques for prognostic factors are described and likely to be valid and reliable, e.g., standardised, repeated
	(c) Continuous variables are reported	Standard categories for prognostic factors / cut‐offs
	(d) Method and setting of measurement of PF is identical for all participants	Measurements of PFs are the same for all study participants
	(e) Adequate proportion of study sample has complete data for PF	Adequate proportion of the study sample has complete data for PF variable
	(f) Appropriate methods of imputation used for missing PF data	Appropriate methods of imputation are used for missing PF data
	**Domain overall risk of bias**	**High:** most items are answered with 'no'**; Low:** all items answered with 'yes'**; Moderate:** most items are answered with 'unclear'
4. Outcome measurement	a) Clear definition of outcome provided	Measurement of proliferative diabetic retinopathy(PDR)/high‐risk characteristics (HRC) is defined
	(b) Method of outcome measurement is adequately valid and reliable	Measurement of PDR/HRC has to be a part of a diagnostic assessment
	(c) Method and setting of outcome measurement is identical for all participants	Measurements of PDR/HRC are the same for all study participants
	**Domain overall risk of bias**	**High:** most items are answered with 'no'**; Low:** all items answered with 'yes'**; Moderate:** most items are answered with 'unclear'
5. Adjustment for other prognostic factors	(a) All other important PFs measured	Important confounders are: HbA1c and duration of DM
	b) Clear definitions of important PFs measured provided	Measurement of confounders has to be clearly described
	(c) Measurement of all important PFs adequately valid and reliable	Measurement of confounders is valid and reliable
	(d) Measurement and setting of PF measurement identical for all participants	Measurements of confounders are the same for all study participants
	(e) Appropriate methods are used to deal with missing values of PFs	Strategy to impute missing confounder data is described
	(f) Important PFs accounted for in study design	Methods section of the publication describes strategy to account for confounders
	(g) Important PFs accounted for in analysis	Important confounders are accounted for in multivariable logistic regression and Cox proportional hazards models
	**Domain overall risk of bias**	**High:** most items are answered with 'no'**; Low:** all items answered with 'yes'**; Moderate:** most items are answered with 'unclear'
6. Statistical analysis and reporting	(a) Sufficient presentation of data to assess adequacy of analytic strategy	Mean or median values, including confidence intervals or standard errors or standard deviations provided
	(b) Strategy for model building appropriate and based on a conceptual framework or model	The selected statistical model is adequate for the design of the study
	(c) Selected statistical model adequate for design of study	Mainly incidence rates, uni‐ and multivariate logistic regression, Cox proportional hazard model
	(d) No selective reporting of results	There is no selective reporting of results
	**Domain overall risk of bias**	**High:** most items are answered with 'no'**; Low:** all items answered with 'yes'**; Moderate:** most items are answered with 'unclear'

## Characteristics of studies

### Characteristics of included studies [ordered by study ID]

**Table:** Arfken 1998

***Study characteristics***		
Methods	Study design: retrospective cohort longitudinal Location: USA Time period: unclear Outcome: PDR Outcome measurement: incidence and OR (95% CI)	
Participants	Number of participants: 312 Diabetes type: T1D Inclusion criteria: T1D (defined as age of onset ≤ 40 years and continuous insulin usage); at least two visits with gradable fundus photographs; African‐American or white; duration of DM ≤ 16 years (relaxed for African‐American group) Exclusion criteria: haemoglobinopathies; PDR; evidence of treatment for PDR at baseline	
Notes	Link to data extraction table, risk of bias assessment, and results: osf.io/krc53/?view_only=bf305ee63ef54bdca777f003087b567c	

**Item**	**Authors' judgement**	**Support for judgement**
Study participation	Unclear	See ‘Notes’ above for link to risk of bias assessment
Study attrition	No	See ‘Notes’ above for link to risk of bias assessment
Prognostic factor measurement	Unclear	See ‘Notes’ above for link to risk of bias assessment
Outcome measurement	Yes	See ‘Notes’ above for link to risk of bias assessment
Adjustment for other prognostic factors	Unclear	See ‘Notes’ above for link to risk of bias assessment
Statistical analysis and reporting	Unclear	See ‘Notes’ above for link to risk of bias assessment

**Table:** Ballard 1986

***Study characteristics***		
Methods	Study design: retrospective cohort longtitudinal Location: USA Time period: DM diagnosed between 1945 and 1969 and followed through complete medical records to 31 Dec 1981 Outcome: PDR Outcome measurement: incidence density, cumulative incidence	
Participants	Number of participants: 1031 Diabetes type: NIDDM Inclusion criteria: NIDDM Exclusion criteria: IDDM; secondary diabetes	
Notes	Link to data extraction table, risk of bias assessment, and results: osf.io/8f5zr/?view_only=1f191b99e38a4e55be1916411610f508	

**Item**	**Authors' judgement**	**Support for judgement**
Study participation	Unclear	See ‘Notes’ above for link to risk of bias assessment
Study attrition	No	See ‘Notes’ above for link to risk of bias assessment
Prognostic factor measurement	Unclear	See ‘Notes’ above for link to risk of bias assessment
Outcome measurement	Unclear	See ‘Notes’ above for link to risk of bias assessment
Adjustment for other prognostic factors	No	See ‘Notes’ above for link to risk of bias assessment
Statistical analysis and reporting	Unclear	See ‘Notes’ above for link to risk of bias assessment

**Table:** Bojestig 1998

***Study characteristics***		
Methods	Study design: prospective cohort longitudinal Location: Sweden Time period: DM diagnosed between 1961 and 1980 and followed up between 1990 and 1992 Outcome: PDR Outcome measurement: cumulative incidence	
Participants	Number of participants: 213 Diabetes type: T1D Inclusion criteria: T1D; onset before the age of 15 years; lived within catchment area of the paediatric clinic, University Hospital, Linkoping, Sweden Exclusion criteria: not reported	
Notes	Link to data extraction table, risk of bias assessment, and results: osf.io/prd4t/?view_only=97fe8cf9a841490dbdb22ff26c542ebc	

**Item**	**Authors' judgement**	**Support for judgement**
Study participation	Unclear	See ‘Notes’ above for link to risk of bias assessment
Study attrition	Unclear	See ‘Notes’ above for link to risk of bias assessment
Prognostic factor measurement	Unclear	See ‘Notes’ above for link to risk of bias assessment
Outcome measurement	Unclear	See ‘Notes’ above for link to risk of bias assessment
Adjustment for other prognostic factors	Unclear	See ‘Notes’ above for link to risk of bias assessment
Statistical analysis and reporting	Unclear	See ‘Notes’ above for link to risk of bias assessment

**Table:** Burditt 1968

***Study characteristics***		
Methods	Study design: retrospective case‐control longitudinal Location: UK Time period: late 1949 to 1968 Outcome: PDR Outcome measurement: incidence	
Participants	Number of participants: 2184 Diabetes type: unclear Inclusion criteria: diagnosis of DM (random blood sugar > 180 mg/100 ml^2^; fasting blood sugar > 130 mg/100 ml^2^; glucose tolerance test ‘florid diabetes’; other evidence e.g. use of insulin) Exclusion criteria: DM known to be associated with chronic pancreatitis, haemochromatosis, or acromegaly	
Notes	Link to data extraction table, risk of bias assessment, and results: osf.io/2b9q3/?view_only=eb515e7e72f3433b80fc94df4714b53f	

**Item**	**Authors' judgement**	**Support for judgement**
Study participation	Unclear	See ‘Notes’ above for link to risk of bias assessment
Study attrition	No	See ‘Notes’ above for link to risk of bias assessment
Prognostic factor measurement	Unclear	See ‘Notes’ above for link to risk of bias assessment
Outcome measurement	Unclear	See ‘Notes’ above for link to risk of bias assessment
Adjustment for other prognostic factors	No	See ‘Notes’ above for link to risk of bias assessment
Statistical analysis and reporting	Unclear	See ‘Notes’ above for link to risk of bias assessment

**Table:** Burgess 2015

***Study characteristics***		
Methods	Study design: retrospective cohort longitudinal Location: Malawi Time period: 2007 to 2012 Outcome: PDR Outcome measurement: incidence	
Participants	Number of participants: 281 Diabetes type: T1D and T2D Inclusion criteria: individuals who had participated in 2007 cross‐sectional study Exclusion criteria: not reported	
Notes	Link to data extraction table, risk of bias assessment, and results: osf.io/6y3rc/?view_only=031a09c802af41cb98933e6a98302a5b	

**Item**	**Authors' judgement**	**Support for judgement**
Study participation	Unclear	See ‘Notes’ above for link to risk of bias assessment
Study attrition	No	See ‘Notes’ above for link to risk of bias assessment
Prognostic factor measurement	Unclear	See ‘Notes’ above for link to risk of bias assessment
Outcome measurement	Yes	See ‘Notes’ above for link to risk of bias assessment
Adjustment for other prognostic factors	No	See ‘Notes’ above for link to risk of bias assessment
Statistical analysis and reporting	Unclear	See ‘Notes’ above for link to risk of bias assessment

**Table:** Chawla 2021

***Study characteristics***		
Methods	Study design: prospective cohort longitudinal Location: India Time period: 2016 to 2019 Outcome: PDR Outcome measurement: incidence	
Participants	Number of participants: 3090 Diabetes type: T2D Inclusion criteria: T2D as defined based by the American Diabetes Association; dietary habits noted before a comprehensive clinical examination and basic assessment for microvascular complications Exclusion criteria: other causes of microalbuminuria (fever, recent vigorous exercise, haematuria, dehydration, urinary tract infection)	
Notes	Link to data extraction table, risk of bias assessment, and results: https://osf.io/k2vrh/?view_only=e191205b9a3a4aabb69bcab3908ca510	

**Item**	**Authors' judgement**	**Support for judgement**
Study participation	Unclear	See ‘Notes’ above for link to risk of bias assessment
Study attrition	No	See ‘Notes’ above for link to risk of bias assessment
Prognostic factor measurement	Unclear	See ‘Notes’ above for link to risk of bias assessment
Outcome measurement	Unclear	See ‘Notes’ above for link to risk of bias assessment
Adjustment for other prognostic factors	No	See ‘Notes’ above for link to risk of bias assessment
Statistical analysis and reporting	Unclear	See ‘Notes’ above for link to risk of bias assessment

**Table:** Chen 1995

***Study characteristics***		
Methods	Study design: prospective cohort longitudinal Location: Taiwan Time period: 1986 to 1990 Outcome: PDR Outcome measurement: cumulative incidence	
Participants	Number of participants: 471 Diabetes type: T2D Inclusion criteria: age ≥ 40 years; fasting or 2‐hour post‐prandial blood glucose level indicating DM according to WHO, or receiving insulin or sulphonylurea agents for diabetic control; ocular fundus clearly visible by ophthalmoscopy Exclusion criteria: history of diabetic ketoacidosis; insulin therapy within one year of diagnosis of diabetes	
Notes	Link to data extraction table, risk of bias assessment, and results: osf.io/6rqcu/?view_only=d40b2521ba98457690f0c5b2ea8a406f	

**Item**	**Authors' judgement**	**Support for judgement**
Study participation	Unclear	See ‘Notes’ above for link to risk of bias assessment
Study attrition	Unclear	See ‘Notes’ above for link to risk of bias assessment
Prognostic factor measurement	Unclear	See ‘Notes’ above for link to risk of bias assessment
Outcome measurement	Yes	See ‘Notes’ above for link to risk of bias assessment
Adjustment for other prognostic factors	No	See ‘Notes’ above for link to risk of bias assessment
Statistical analysis and reporting	Unclear	See ‘Notes’ above for link to risk of bias assessment

**Table:** Cho 2019

***Study characteristics***		
Methods	Study design: retrospective cohort longitudinal Location: South Korea Time period: 2006 to 2014 Outcome: PDR Outcome measurement: prevalence, OR	
Participants	Number of participants: 1527 Diabetes type: T2D Inclusion criteria: T2D from the diabetes clinic in the Department of Endocrinology of Kangnam Sacred Heart Hospital who underwent fundus photographic examinations for DR and whose renal profiles were studied between August 2006 and February 2014 Exclusion criteria: estimated glomerular filtration rate < 15 mL/min/1:73 m^2^ and without follow‐up renal profiles; fundus exam obtained more than 3 months after the first evaluation	
Notes	Link to data extraction table, risk of bias assessment, and results: osf.io/3qt9w/?view_only=03b2f088652d44de83fe308b6e1b5070	

**Item**	**Authors' judgement**	**Support for judgement**
Study participation	Unclear	See ‘Notes’ above for link to risk of bias assessment
Study attrition	No	See ‘Notes’ above for link to risk of bias assessment
Prognostic factor measurement	Unclear	See ‘Notes’ above for link to risk of bias assessment
Outcome measurement	Unclear	See ‘Notes’ above for link to risk of bias assessment
Adjustment for other prognostic factors	Unclear	See ‘Notes’ above for link to risk of bias assessment
Statistical analysis and reporting	Yes	See ‘Notes’ above for link to risk of bias assessment

**Table:** Dwyer 1985

***Study characteristics***		
Methods	Study design: registry data Location: USA Time period: diagnosed 1945 to 1969, followed until December 1981 Outcome: PDR Outcome measurement: incidence	
Participants	Number of participants: 1135 Diabetes type: T1D and T2D Inclusion criteria: new DM diagnosis 1945 to 1969 Exclusion criteria: not reported	
Notes	Link to data extraction table, risk of bias assessment, and results: osf.io/w2mq9/?view_only=d23bc4f8bf7045c3a5089f140af86fa2	

**Item**	**Authors' judgement**	**Support for judgement**
Study participation	Unclear	See ‘Notes’ above for link to risk of bias assessment
Study attrition	No	See ‘Notes’ above for link to risk of bias assessment
Prognostic factor measurement	Unclear	See ‘Notes’ above for link to risk of bias assessment
Outcome measurement	Unclear	See ‘Notes’ above for link to risk of bias assessment
Adjustment for other prognostic factors	No	See ‘Notes’ above for link to risk of bias assessment
Statistical analysis and reporting	Unclear	See ‘Notes’ above for link to risk of bias assessment

**Table:** Gange 2021

***Study characteristics***		
Methods	Study design: registry data Location: USA Time period: 2007 to 2015 Outcome: PDR Outcome measurement: cumulative incidence, OR, means (SD) and percentages (SD) of progressors versus non‐progressors to PDR	
Participants	Number of participants: 277,401 Diabetes type: T2D Inclusion criteria: insured patients aged ≥ 18 yrs; newly diagnosed T2D; continuous enrolment for 12 months without a diabetes diagnosis or any diabetes medication use Exclusion criteria: concurrent pregnancy; gestational diabetes; T1D; use of an insulin pump; diagnosis of diabetic eye disease prior to the diagnosis of diabetes	
Notes	Link to data extraction table, risk of bias assessment, and results: osf.io/x6fub/?view_only=876e299959fc4b89abed3a24d82e3fc9	

**Item**	**Authors' judgement**	**Support for judgement**
Study participation	Unclear	See ‘Notes’ above for link to risk of bias assessment
Study attrition	No	See ‘Notes’ above for link to risk of bias assessment
Prognostic factor measurement	Unclear	See ‘Notes’ above for link to risk of bias assessment
Outcome measurement	Unclear	See ‘Notes’ above for link to risk of bias assessment
Adjustment for other prognostic factors	Unclear	See ‘Notes’ above for link to risk of bias assessment
Statistical analysis and reporting	Unclear	See ‘Notes’ above for link to risk of bias assessment

**Table:** Grauslund 2009

***Study characteristics***		
Methods	Study design: prospective cohort longitudinal Location: Denmark Time period: baseline 1981 to 82; follow‐up 2007 to 2008 Outcome: PDR Outcome measurement: incidence and OR (95% CI)	
Participants	Number of participants: 573 Diabetes type: T1D Inclusion criteria: all T1D patients from Fyn County, Denmark, with DM onset before 30 years of age, identified based on insulin prescription as of 1 July 1973 Exclusion criteria: not reported	
Notes	Link to data extraction table, risk of bias assessment, and results: osf.io/ef7w9/?view_only=6fd044128cb14a4592c3df877f0507bc	

**Item**	**Authors' judgement**	**Support for judgement**
Study participation	Unclear	See ‘Notes’ above for link to risk of bias assessment
Study attrition	Unclear	See ‘Notes’ above for link to risk of bias assessment
Prognostic factor measurement	Unclear	See ‘Notes’ above for link to risk of bias assessment
Outcome measurement	Yes	See ‘Notes’ above for link to risk of bias assessment
Adjustment for other prognostic factors	Unclear	See ‘Notes’ above for link to risk of bias assessment
Statistical analysis and reporting	Unclear	See ‘Notes’ above for link to risk of bias assessment

**Table:** Gui 2013

***Study characteristics***		
Methods	Study design: retrospective cohort Location: China Time period: Recruitment: 1 January 2009 to 1 January 2010 Follow‐up: January to May 2012 Outcome: PDR Outcome measurement: cumulative incidence, OR, means and percentages of progressors versus non‐progressors to PDR	
Participants	Number of participants: 205 Diabetes type: T2D Inclusion criteria: T2D; NPDR Exclusion criteria: not reported	
Notes	Link to data extraction table, risk of bias assessment, and results: osf.io/psj4v/?view_only=70ca392da5cb4d7cb069361dbb4eda03	

**Item**	**Authors' judgement**	**Support for judgement**
Study participation	Unclear	See ‘Notes’ above for link to risk of bias assessment
Study attrition	Unclear	See ‘Notes’ above for link to risk of bias assessment
Prognostic factor measurement	Unclear	See ‘Notes’ above for link to risk of bias assessment
Outcome measurement	Unclear	See ‘Notes’ above for link to risk of bias assessment
Adjustment for other prognostic factors	Unclear	See ‘Notes’ above for link to risk of bias assessment
Statistical analysis and reporting	Yes	See ‘Notes’ above for link to risk of bias assessment

**Table:** Gurreri 2019

***Study characteristics***		
Methods	Study design: retrospective cohort Location: Italy Time period: February 2012 to December 2017 Outcome: PDR Outcome measurement: incidence	
Participants	Number of participants: 479 Diabetes type: T2D Inclusion criteria: T2D with NPDR; patients who visited department between February 2012 and December 2017 Exclusion criteria: T1D, PDR, age‐related macular degeneration, or other unrelated retinal conditions, incomplete data, or non‐adherence to therapy	
Notes	Link to data extraction table, risk of bias assessment, and results: osf.io/ytkeh/?view_only=e67462dbac7c4919a51db2ddd32b2319	

**Item**	**Authors' judgement**	**Support for judgement**
Study participation	Unclear	See ‘Notes’ above for link to risk of bias assessment
Study attrition	No	See ‘Notes’ above for link to risk of bias assessment
Prognostic factor measurement	Unclear	See ‘Notes’ above for link to risk of bias assessment
Outcome measurement	Yes	See ‘Notes’ above for link to risk of bias assessment
Adjustment for other prognostic factors	No	See ‘Notes’ above for link to risk of bias assessment
Statistical analysis and reporting	Unclear	See ‘Notes’ above for link to risk of bias assessment

**Table:** Hardin 1956

***Study characteristics***		
Methods	Study design: prospective cohort Location: USA Time period: period ending September 1954 Outcome: PDR Outcome measurement: incidence	
Participants	Number of participants: 132 Diabetes type: T2D Inclusion criteria: juvenile diabetes (developed disease aged 6 months to 19 years); second decade of diabetes Exclusion criteria: not reported	
Notes	Link to data extraction table, risk of bias assessment, and results: osf.io/vwcn2/?view_only=98b37d2b3c364592b1f421bd4ffd5e61	

**Item**	**Authors' judgement**	**Support for judgement**
Study participation	Unclear	See ‘Notes’ above for link to risk of bias assessment
Study attrition	Unclear	See ‘Notes’ above for link to risk of bias assessment
Prognostic factor measurement	Unclear	See ‘Notes’ above for link to risk of bias assessment
Outcome measurement	Unclear	See ‘Notes’ above for link to risk of bias assessment
Adjustment for other prognostic factors	No	See ‘Notes’ above for link to risk of bias assessment
Statistical analysis and reporting	Unclear	See ‘Notes’ above for link to risk of bias assessment

**Table:** Harris 2013

***Study characteristics***		
Methods	Study design: registry data Location: USA Time period: 01 January 2001 to 31 December 2009 Outcome: PDR Outcome measurement: incidence, means, and percentages of progressors versus non‐progressors to PDR, and HR	
Participants	Number of participants: 4617 Diabetes type: T1D and T2D Inclusion criteria: new diagnosis of NPDR after first year in registry (point of baseline); aged ≥ 30 years; ≥ 2 registrations as having diagnosis of DM; continuous enrolment in registry; ≥ 1 visit to an ophthalmologist or optometrist during first year of registration and no signs of NPDR or PDR; ≥ 1 record of HbA1c following baseline date Exclusion criteria: in registry < 1 year; not in registry continuously; any record of PDR prior to index date	
Notes	Link to data extraction table, risk of bias assessment, and results: osf.io/t52qu/?view_only=37b711fa179d4b249d9d16c430ec169e	

**Item**	**Authors' judgement**	**Support for judgement**
Study participation	Unclear	See ‘Notes’ above for link to risk of bias assessment
Study attrition	No	See ‘Notes’ above for link to risk of bias assessment
Prognostic factor measurement	Unclear	See ‘Notes’ above for link to risk of bias assessment
Outcome measurement	Unclear	See ‘Notes’ above for link to risk of bias assessment
Adjustment for other prognostic factors	Unclear	See ‘Notes’ above for link to risk of bias assessment
Statistical analysis and reporting	Unclear	See ‘Notes’ above for link to risk of bias assessment

**Table:** Hovind 2003

***Study characteristics***		
Methods	Study design: prospective cohort Location: Denmark Time period: baseline: onset of T1D 1965 to 1984, followed until 2000 Outcome: PDR Outcome measurement: cumulative incidence	
Participants	Number of participants: not reported Diabetes type: T1D Inclusion criteria: groups A to C ‐ all T1D patients at Hvidovre Hospital with onset of diabetes between 1965 and 1979, and before the age of 41 years, and who were 18 years of age at the time of study; group D ‐ all newly diagnosed T1D patients referred to the Steno Memorial Hospital between 1 September 1979 and 31 August 1984 Exclusion criteria: mentally ill; onset of other serious competing medical or psychosocial conditions	
Notes	Link to data extraction table, risk of bias assessment, and results: osf.io/jmkeu/?view_only=cebee4c24f3042b8aa34e045b8acd90e	

**Item**	**Authors' judgement**	**Support for judgement**
Study participation	Unclear	See ‘Notes’ above for link to risk of bias assessment
Study attrition	Unclear	See ‘Notes’ above for link to risk of bias assessment
Prognostic factor measurement	Unclear	See ‘Notes’ above for link to risk of bias assessment
Outcome measurement	Unclear	See ‘Notes’ above for link to risk of bias assessment
Adjustment for other prognostic factors	No	See ‘Notes’ above for link to risk of bias assessment
Statistical analysis and reporting	Unclear	See ‘Notes’ above for link to risk of bias assessment

**Table:** Hsieh 2018

***Study characteristics***		
Methods	Study design: prospective cohort Location: Taiwan Time period: baseline: April 2002 to September 2004; end of study: 31 December 10 Outcome: PDR Outcome measurement: incidence and HR	
Participants	Number of participants: 2135 Diabetes type: T2D Inclusion criteria: patients who received a diagnosis of T2D and underwent treatment in the outpatient clinic of the Metabolism Division at Changhua Christian Hospital and Kaohsiung Medical University Hospital between April 2002 and September 2004 Exclusion criteria: lost to follow‐up within 6 months; ungradable image results from both eyes at baseline	
Notes	Link to data extraction table, risk of bias assessment, and results: osf.io/759nk/?view_only=cbb8e5efe90b4af5979af0f889818c26	

**Item**	**Authors' judgement**	**Support for judgement**
Study participation	Unclear	See ‘Notes’ above for link to risk of bias assessment
Study attrition	Unclear	See ‘Notes’ above for link to risk of bias assessment
Prognostic factor measurement	Unclear	See ‘Notes’ above for link to risk of bias assessment
Outcome measurement	Yes	See ‘Notes’ above for link to risk of bias assessment
Adjustment for other prognostic factors	Unclear	See ‘Notes’ above for link to risk of bias assessment
Statistical analysis and reporting	Yes	See ‘Notes’ above for link to risk of bias assessment

**Table:** Janghorbani 2000

***Study characteristics***		
Methods	Study design: retrospective cohort Location: UK Time period: 1979 to 1992 Outcome: PDR Outcome measurement: incidence, RR, and mean difference between progressors and non‐progressors to PDR	
Participants	Number of participants: 3482 Diabetes type: T1D and T2D Inclusion criteria: NIDDM or IDDM; free of PDR (including those with no retinopathy and those with NPDR at registration); complete data available Exclusion criteria: secondary diabetes; type of diabetes unknown	
Notes	Link to data extraction table, risk of bias assessment, and results: osf.io/3xfrg/?view_only=baa50019fd8b4a1898e78ad977ed9398	

**Item**	**Authors' judgement**	**Support for judgement**
Study participation	Yes	See ‘Notes’ above for link to risk of bias assessment
Study attrition	No	See ‘Notes’ above for link to risk of bias assessment
Prognostic factor measurement	Unclear	See ‘Notes’ above for link to risk of bias assessment
Outcome measurement	Unclear	See ‘Notes’ above for link to risk of bias assessment
Adjustment for other prognostic factors	Unclear	See ‘Notes’ above for link to risk of bias assessment
Statistical analysis and reporting	Yes	See ‘Notes’ above for link to risk of bias assessment

**Table:** Jeng 2016

***Study characteristics***		
Methods	Study design: registry data/case‐control study Location: Taiwan Time period: 01 January 2000 to 31 December 2010 Outcome: PDR Outcome measurement: cumulative incidence, incidence per 1000 person‐years, and HR	
Participants	Number of participants: 53453 Diabetes type: unclear Inclusion criteria: diabetic nephropathy (DN) cohort: ≥ 18‐year old patients with DM plus DN diagnosed between 01 January 2000 to 31 December 2010. Non‐DN cohort: diagnosis of DN not made during 01 January 2000 to 31 December 2010. Exclusion criteria: not reported	
Notes	Link to data extraction table, risk of bias assessment, and results: osf.io/h95wu/?view_only=e18a5b64b3244a3987aa4520c779ee54	

**Item**	**Authors' judgement**	**Support for judgement**
Study participation	No	See ‘Notes’ above for link to risk of bias assessment
Study attrition	No	See ‘Notes’ above for link to risk of bias assessment
Prognostic factor measurement	Unclear	See ‘Notes’ above for link to risk of bias assessment
Outcome measurement	No	See ‘Notes’ above for link to risk of bias assessment
Adjustment for other prognostic factors	No	See ‘Notes’ above for link to risk of bias assessment
Statistical analysis and reporting	Yes	See ‘Notes’ above for link to risk of bias assessment

**Table:** Jones 2012

***Study characteristics***		
Methods	Study design: prospective cohort longitudinal Location: UK Time period: January 1990 to December 2006 Outcome: PDR Outcome measurement: cumulative incidence	
Participants	Number of participants: 3632 Diabetes type: T1D and T2D Inclusion criteria: screened at Central Norfolk DR Screening Service between January 1990 and December 2006 Exclusion criteria: sight‐threatening maculopathy or PDR at first retinal exam	
Notes	Link to data extraction table, risk of bias assessment, and results: osf.io/gzan9/?view_only=f42d9d00afaf40f9ad6872fb841e754e	

**Item**	**Authors' judgement**	**Support for judgement**
Study participation	Unclear	See ‘Notes’ above for link to risk of bias assessment
Study attrition	No	See ‘Notes’ above for link to risk of bias assessment
Prognostic factor measurement	Unclear	See ‘Notes’ above for link to risk of bias assessment
Outcome measurement	Unclear	See ‘Notes’ above for link to risk of bias assessment
Adjustment for other prognostic factors	No	See ‘Notes’ above for link to risk of bias assessment
Statistical analysis and reporting	Unclear	See ‘Notes’ above for link to risk of bias assessment

**Table:** Kalter‐Leibovici 1991

***Study characteristics***		
Methods	Study design: prospective cohort longitudinal Location: Israel Time period: not reported Outcome: PDR Outcome measurement: incidence, OR, and median and percentages of progressors versus non‐progressors to PDR	
Participants	Number of participants: 330 Diabetes type: T1D Inclusion criteria: all Jewish patients attending centre with early‐onset IDDM before 30 years of age and DM duration of ≥ 10 years Exclusion criteria: not reported	
Notes	Link to data extraction table, risk of bias assessment, and results: osf.io/tz8bc/?view_only=5d94c5615d7847418d616db8f0ddd107	

**Item**	**Authors' judgement**	**Support for judgement**
Study participation	Unclear	See ‘Notes’ above for link to risk of bias assessment
Study attrition	No	See ‘Notes’ above for link to risk of bias assessment
Prognostic factor measurement	Unclear	See ‘Notes’ above for link to risk of bias assessment
Outcome measurement	Unclear	See ‘Notes’ above for link to risk of bias assessment
Adjustment for other prognostic factors	Unclear	See ‘Notes’ above for link to risk of bias assessment
Statistical analysis and reporting	Yes	See ‘Notes’ above for link to risk of bias assessment

**Table:** Keen 2001

***Study characteristics***		
Methods	Study design: prospective cohort longitudinal Location: multinational (10 centres) Time period: 1975 to 1978; follow‐up study conducted between 1983 and 1986 (in Oklahoma between 1988 and 1990) Outcome: PDR Outcome measurement: cumulative incidence and OR	
Participants	Number of participants: 4483 Diabetes type: T1D and T2D Inclusion criteria: the study protocol required equal numbers of men and women with diabetes, sampled from three age bands within the range 35 to 54 years Exclusion criteria: not reported	
Notes	Link to data extraction table, risk of bias assessment, and results: osf.io/mejrs/?view_only=7dda7dbbfcea4b78ac3bef421141ff80	

**Item**	**Authors' judgement**	**Support for judgement**
Study participation	Unclear	See ‘Notes’ above for link to risk of bias assessment
Study attrition	No	See ‘Notes’ above for link to risk of bias assessment
Prognostic factor measurement	Unclear	See ‘Notes’ above for link to risk of bias assessment
Outcome measurement	Unclear	See ‘Notes’ above for link to risk of bias assessment
Adjustment for other prognostic factors	No	See ‘Notes’ above for link to risk of bias assessment
Statistical analysis and reporting	Unclear	See ‘Notes’ above for link to risk of bias assessment

**Table:** Kim 1998

***Study characteristics***		
Methods	Study design: prospective cohort longitudinal Location: South Korea Time period: Recruitment 1990 to 1991 Outcome: PDR Outcome measurement: incidence, means of progressors versus non‐progressors to PDR, and RR	
Participants	Number of participants: 56 Diabetes type: T2D Inclusion criteria: individuals attending a university hospital (the Asan Medical Center) in Seoul, Korea; NIDDM diagnosis based on clinical characteristics that included no episodes of ketoacidosis, a diagnosis of diabetes after 30 years of age and treatment by diet and/or oral hypoglycaemic agents, or fasting serum C‐peptide values > 0.30 nmol/L in patients using insulin Exclusion criteria: not reported	
Notes	Link to data extraction table, risk of bias assessment, and results: osf.io/daecn/?view_only=9a57929592c24d4588ba5646c089e6d1	

**Item**	**Authors' judgement**	**Support for judgement**
Study participation	Unclear	See ‘Notes’ above for link to risk of bias assessment
Study attrition	Unclear	See ‘Notes’ above for link to risk of bias assessment
Prognostic factor measurement	Unclear	See ‘Notes’ above for link to risk of bias assessment
Outcome measurement	Unclear	See ‘Notes’ above for link to risk of bias assessment
Adjustment for other prognostic factors	Unclear	See ‘Notes’ above for link to risk of bias assessment
Statistical analysis and reporting	Yes	See ‘Notes’ above for link to risk of bias assessment

**Table:** Kim 2014

***Study characteristics***		
Methods	Study design: retrospective cohort longitudinal Location: South Korea Time period: followed up since 2000 Outcome: PDR Outcome measurement: incidence, HR (95% CI), and means and percentages of progressors versus non‐progressors to PDR	
Participants	Number of participants: 452 Diabetes type: T2D Inclusion criteria: patients who were diagnosed with T2D and followed annually or more often for more than 5 years at a hospital‐based diabetic clinic (Asan Medical Center, Seoul, South Korea) Exclusion criteria: PDR at the initial examination, with concomitant ocular disease other than DR, or history of ocular trauma or intraocular surgery were excluded	
Notes	Link to data extraction table, risk of bias assessment, and results: osf.io/ks6ep/?view_only=4f4b86c4841e4f8497ccc16c30f7a4d3	

**Item**	**Authors' judgement**	**Support for judgement**
Study participation	Unclear	See ‘Notes’ above for link to risk of bias assessment
Study attrition	Unclear	See ‘Notes’ above for link to risk of bias assessment
Prognostic factor measurement	Unclear	See ‘Notes’ above for link to risk of bias assessment
Outcome measurement	Unclear	See ‘Notes’ above for link to risk of bias assessment
Adjustment for other prognostic factors	Unclear	See ‘Notes’ above for link to risk of bias assessment
Statistical analysis and reporting	Yes	See ‘Notes’ above for link to risk of bias assessment

**Table:** Klein 1984

***Study characteristics***		
Methods	Study design: prospective cohort longitudinal Location: USA Time period: baseline: 1970 to 1971; follow‐up: 1976 to 1977 Outcome: PDR Outcome measurement: incidence	
Participants	Number of participants: 191 Diabetes type: T1D Inclusion criteria: on insulin since DM diagnosis, if asymptomatic and diagnosed through routine examination must have become symptomatic and taken insulin within one year of diagnosis; ≥ 5 years duration; under care of cooperating GPs for at least 2/3 of the duration of DM Exclusion criteria: overweight; ≥ 50 years	
Notes	Link to data extraction table, risk of bias assessment, and results: osf.io/cz5vn/?view_only=940908b18a9c4a14873f1cf4ab26c03a	

**Item**	**Authors' judgement**	**Support for judgement**
Study participation	Unclear	See ‘Notes’ above for link to risk of bias assessment
Study attrition	Unclear	See ‘Notes’ above for link to risk of bias assessment
Prognostic factor measurement	Unclear	See ‘Notes’ above for link to risk of bias assessment
Outcome measurement	Yes	See ‘Notes’ above for link to risk of bias assessment
Adjustment for other prognostic factors	No	See ‘Notes’ above for link to risk of bias assessment
Statistical analysis and reporting	Unclear	See ‘Notes’ above for link to risk of bias assessment

**Table:** Kofoed‐Enevoldsen 1987

***Study characteristics***		
Methods	Study design: retrospective case‐control longitudinal Location: Denmark Time period: 1975 to 1982; December 1985 Outcome: PDR Outcome measurement: incidence	
Participants	Number of participants: 220 Diabetes type: T1D Inclusion criteria: group A: persistent proteinuria; group B: matched to group A with regard to gender and year and age at diabetes onset, but free of proteinuria during the study period Exclusion criteria: not reported	
Notes	Link to data extraction table, risk of bias assessment, and results: osf.io/qp7b2/?view_only=229b89f8cd7d43538e0aab26340c16cc	

**Item**	**Authors' judgement**	**Support for judgement**
Study participation	Unclear	See ‘Notes’ above for link to risk of bias assessment
Study attrition	No	See ‘Notes’ above for link to risk of bias assessment
Prognostic factor measurement	Unclear	See ‘Notes’ above for link to risk of bias assessment
Outcome measurement	Unclear	See ‘Notes’ above for link to risk of bias assessment
Adjustment for other prognostic factors	No	See ‘Notes’ above for link to risk of bias assessment
Statistical analysis and reporting	Unclear	See ‘Notes’ above for link to risk of bias assessment

**Table:** Kullberg 1993

***Study characteristics***		
Methods	Study design: retrospective cohort longitudinal Location: Sweden Time period: 01 August 1988 to 31 October 1991 Outcome: PDR Outcome measurement: incidence and means of progressors versus non‐progressors to PDR	
Participants	Number of participants: 172 Diabetes type: T1D Inclusion criteria: all T1D patients attending the diabetic outpatient clinic at the University Hospital, Linkoping during the period 01 August 1988 to 31 October 1991, with age at diagnosis of less than 31 years, diabetes duration 25 years or less, and with glycated haemoglobin measured for at least 5 years at the hospital, in the absence of or before the appearance of PDR or nephropathy Exclusion criteria: not explicitly stated as criteria, but excluded those with multiple endocrine insufficiency and hypothyroidism with impaired renal function, abnormal haemoglobin, and pregnancy	
Notes	Link to data extraction table, risk of bias assessment, and results: osf.io/fkmzr/?view_only=7fbd4103b88d4f9d8cf7c5c8e34a91e8	

**Item**	**Authors' judgement**	**Support for judgement**
Study participation	Unclear	See ‘Notes’ above for link to risk of bias assessment
Study attrition	No	See ‘Notes’ above for link to risk of bias assessment
Prognostic factor measurement	Unclear	See ‘Notes’ above for link to risk of bias assessment
Outcome measurement	Yes	See ‘Notes’ above for link to risk of bias assessment
Adjustment for other prognostic factors	Unclear	See ‘Notes’ above for link to risk of bias assessment
Statistical analysis and reporting	Unclear	See ‘Notes’ above for link to risk of bias assessment

**Table:** Lee 1992

***Study characteristics***		
Methods	Study design: prospective cohort longitudinal Location: USA Time period: 1972 to 1990 Outcome: PDR Outcome measurement: cumulative incidence, means of progressors versus non‐progressors to PDR, and RR	
Participants	Number of participants: 354 Diabetes type: T2D Inclusion criteria: NIDDM (no further description); Oklahoma Indians (understood to be Native Americans) examined at the Indian Health Service facilities in Oklahoma; fasting plasma glucose > 7.8 mmol (140 mg/dL) or a 2‐hour post‐load blood glucose level > 11.1 mmol (200 mg/dL); diagnoses of DM between 1937 and 1980 Exclusion criteria: PDR at baseline	
Notes	Link to data extraction table, risk of bias assessment, and results: osf.io/by7rg/?view_only=efe5c7e01b0a4c2d8ab0a55cdc05fb08	

**Item**	**Authors' judgement**	**Support for judgement**
Study participation	Unclear	See ‘Notes’ above for link to risk of bias assessment
Study attrition	Unclear	See ‘Notes’ above for link to risk of bias assessment
Prognostic factor measurement	Unclear	See ‘Notes’ above for link to risk of bias assessment
Outcome measurement	Yes	See ‘Notes’ above for link to risk of bias assessment
Adjustment for other prognostic factors	Unclear	See ‘Notes’ above for link to risk of bias assessment
Statistical analysis and reporting	Unclear	See ‘Notes’ above for link to risk of bias assessment

**Table:** Lee 2017

***Study characteristics***		
Methods	Study design: registry data Location: UK Time period: 2007 to 2014; data extracted November 2014 Outcome: PDR Outcome measurement: incidence and HR	
Participants	Number of participants: 32,553 Diabetes type: T1D and T2D Inclusion criteria: first‐time presenters to eye providers after being referred from the UK national DR screening program; at least 2 DR assessments Exclusion criteria: anti–vascular endothelial growth factor injections during study period; eyes with neovascularisation at baseline were excluded from survival analyses	
Notes	Link to data extraction table, risk of bias assessment, and results: osf.io/xzbwg/?view_only=c8bb1357ce82409eaa0caa59cc1df5d7	

**Item**	**Authors' judgement**	**Support for judgement**
Study participation	Unclear	See ‘Notes’ above for link to risk of bias assessment
Study attrition	No	See ‘Notes’ above for link to risk of bias assessment
Prognostic factor measurement	Unclear	See ‘Notes’ above for link to risk of bias assessment
Outcome measurement	Unclear	See ‘Notes’ above for link to risk of bias assessment
Adjustment for other prognostic factors	No	See ‘Notes’ above for link to risk of bias assessment
Statistical analysis and reporting	Yes	See ‘Notes’ above for link to risk of bias assessment

**Table:** Lee 2021

***Study characteristics***		
Methods	Study design: retrospective cohort Location: Taiwan Time period: 12 October 2012 to 11 September 2018 Outcome: PDR Outcome measurement: HR	
Participants	Number of participants: 2626 Diabetes type: T2D Inclusion criteria: T2D with more than two fundus colour photography tests Exclusion criteria: without T2D; no HbA1c or fasting plasma glucose tests within 14 days of the study start; PDR	
Notes	Link to data extraction table, risk of bias assessment, and results: osf.io/y7mu2/?view_only=4001f0cef1cf4908b0df27446ffb93d8	

**Item**	**Authors' judgement**	**Support for judgement**
Study participation	Unclear	See ‘Notes’ above for link to risk of bias assessment
Study attrition	No	See ‘Notes’ above for link to risk of bias assessment
Prognostic factor measurement	Unclear	See ‘Notes’ above for link to risk of bias assessment
Outcome measurement	Unclear	See ‘Notes’ above for link to risk of bias assessment
Adjustment for other prognostic factors	No	See ‘Notes’ above for link to risk of bias assessment
Statistical analysis and reporting	Unclear	See ‘Notes’ above for link to risk of bias assessment

**Table:** Lestradet 1981

***Study characteristics***		
Methods	Study design: prospective cohort Location: France Time period: 1949 to 1976 Outcome: PDR Outcome measurement: incidence	
Participants	Number of participants: 372 Diabetes type: T1D Inclusion criteria: all IDD children (age at onset less than 16 years), whose diabetes was diagnosed between October 1949 and December 1960; followed from the onset of their disease until December 1976 by the Department of Diabetology at the Harold Hospital in Paris Exclusion criteria: patients previously diagnosed outside the department	
Notes	Link to data extraction table, risk of bias assessment, and results: osf.io/rcdpf/?view_only=ac0ce7cab53b43a8b1d17be32afaf799	

**Item**	**Authors' judgement**	**Support for judgement**
Study participation	Unclear	See ‘Notes’ above for link to risk of bias assessment
Study attrition	No	See ‘Notes’ above for link to risk of bias assessment
Prognostic factor measurement	Unclear	See ‘Notes’ above for link to risk of bias assessment
Outcome measurement	Unclear	See ‘Notes’ above for link to risk of bias assessment
Adjustment for other prognostic factors	No	See ‘Notes’ above for link to risk of bias assessment
Statistical analysis and reporting	Unclear	See ‘Notes’ above for link to risk of bias assessment

**Table:** Lloyd 1995

***Study characteristics***		
Methods	Study design: prospective cohort Location: USA Time period: baseline 1986 to 1988; follow‐up 1988 to 1990 Outcome: PDR Outcome measurement: incidence, mean difference, and RR	
Participants	Number of participants: 496 Diabetes type: T1D Inclusion criteria: childhood‐onset < 17 years at the Hospital of Pittsburgh between January 1950 and May 1980, and living within 100 miles of Pittsburgh at the time of the study Exclusion criteria: not reported	
Notes	Link to data extraction table, risk of bias assessment, and results: osf.io/shcf9/?view_only=814f3f5f86874f3d9ee5412730151dca	

**Item**	**Authors' judgement**	**Support for judgement**
Study participation	Unclear	See ‘Notes’ above for link to risk of bias assessment
Study attrition	Unclear	See ‘Notes’ above for link to risk of bias assessment
Prognostic factor measurement	Unclear	See ‘Notes’ above for link to risk of bias assessment
Outcome measurement	Yes	See ‘Notes’ above for link to risk of bias assessment
Adjustment for other prognostic factors	Unclear	See ‘Notes’ above for link to risk of bias assessment
Statistical analysis and reporting	Yes	See ‘Notes’ above for link to risk of bias assessment

**Table:** Mathiesen 1990

***Study characteristics***		
Methods	Study design: prospective cohort Location: Denmark Time period: recruitment: October 1982 to January 1983; follow‐up: January 1988 Outcome: PDR Outcome measurement: incidence	
Participants	Number of participants: 209 Diabetes type: T1D Inclusion criteria: age 18 to 50 years; onset of IDDM before 30 years; duration of diabetes 10 to 30 years; regular attendance in outpatient clinic at Steno Diabetes Centre; diastolic blood pressure < 100 mmHg; sterile urine with normal urinary microscopy; urine albumin excretion (UAE) < 30 mg/24 hours in one 24‐hour urine sample collected at home; during the first year of observation, at least 2 of 3 UAE tests had to be within normal range to secure an observation period with normoalbuminuria Exclusion criteria: not reported	
Notes	Link to data extraction table, risk of bias assessment, and results: osf.io/3hk45/?view_only=9f362bec79d6448ab00f9dccff055bba	

**Item**	**Authors' judgement**	**Support for judgement**
Study participation	Unclear	See ‘Notes’ above for link to risk of bias assessment
Study attrition	No	See ‘Notes’ above for link to risk of bias assessment
Prognostic factor measurement	Unclear	See ‘Notes’ above for link to risk of bias assessment
Outcome measurement	No	See ‘Notes’ above for link to risk of bias assessment
Adjustment for other prognostic factors	Unclear	See ‘Notes’ above for link to risk of bias assessment
Statistical analysis and reporting	Unclear	See ‘Notes’ above for link to risk of bias assessment

**Table:** McCance 1989

***Study characteristics***		
Methods	Study design: retrospective cohort longitudinal Location: UK Time period: diagnosed between January 1968 and December 1986 Outcome: PDR Outcome measurement: incidence and median difference	
Participants	Number of participants: 271 Diabetes type: T1D Inclusion criteria: classic insulin‐dependent diabetes diagnosed between January 1968 and December 1986; 25 years or younger at diagnosis Exclusion criteria: unable to be contacted; failed to attend; received diabetic care abroad; in prison, refused to participate, temporarily abroad or died; congenital nystagmus; medial opacities preventing retinal photography	
Notes	Link to data extraction table, risk of bias assessment, and results: osf.io/t2bzq/?view_only=ad9a16f7610a4e7385e115d13dedc3dc	

**Item**	**Authors' judgement**	**Support for judgement**
Study participation	Unclear	See ‘Notes’ above for link to risk of bias assessment
Study attrition	No	See ‘Notes’ above for link to risk of bias assessment
Prognostic factor measurement	Unclear	See ‘Notes’ above for link to risk of bias assessment
Outcome measurement	Yes	See ‘Notes’ above for link to risk of bias assessment
Adjustment for other prognostic factors	No	See ‘Notes’ above for link to risk of bias assessment
Statistical analysis and reporting	Unclear	See ‘Notes’ above for link to risk of bias assessment

**Table:** McCarty 2003

***Study characteristics***		
Methods	Study design: prospective cohort longitudinal Location: Australia Time period: baseline examinations between 1992 and 1994; follow‐up data collected 5 years later Outcome: PDR Outcome measurement: incidence	
Participants	Number of participants: 169 Diabetes type: T1D and T2D Inclusion criteria: to be eligible for the Melbourne Visual Impairment Project, participants "had to be 40 years of age or older or turned 40 in the current calendar year and resident at the target address for six months or more" Exclusion criteria: "family members who are institutionalised; relatives visiting for a vacation (for less than 6 months); friend(s)/partner who lived in the household on a part‐time basis and has/have a permanent residence outside of the sample area; residents on an extended vacation during the testing period in the sample area; a resident who dies after the initial contact but before examination; and an eligible resident who cannot be contacted after 10 attempts" Inclusion and exclusion criteria obtained from the publication 'Methods for a population‐based study of eye disease: the Melbourne Visual Impairment Project' (McCarty 2003, secondary reference). Unclear eligibility criteria for this particular study.	
Notes	Link to data extraction table, risk of bias assessment, and results: osf.io/ek8t9/?view_only=99134c6188da4c7f813d86d0b144f193	

**Item**	**Authors' judgement**	**Support for judgement**
Study participation	No	See ‘Notes’ above for link to risk of bias assessment
Study attrition	Unclear	See ‘Notes’ above for link to risk of bias assessment
Prognostic factor measurement	Yes	See ‘Notes’ above for link to risk of bias assessment
Outcome measurement	Yes	See ‘Notes’ above for link to risk of bias assessment
Adjustment for other prognostic factors	No	See ‘Notes’ above for link to risk of bias assessment
Statistical analysis and reporting	No	See ‘Notes’ above for link to risk of bias assessment

**Table:** Miki 1969

***Study characteristics***		
Methods	Study design: prospective cohort longitudinal Location: Tokyo, Japan Time period: 1961 to 1967 Outcome: PDR Outcome measurement: incidence	
Participants	Number of participants: 364 Diabetes type: unclear Inclusion criteria: ambulatory participants followed regularly for more than 2 years Exclusion criteria: not reported	
Notes	Link to data extraction table, risk of bias assessment, and results: osf.io/zqtjp/?view_only=aec8c2150af54750b293894ae53284b4	

**Item**	**Authors' judgement**	**Support for judgement**
Study participation	Unclear	See ‘Notes’ above for link to risk of bias assessment
Study attrition	No	See ‘Notes’ above for link to risk of bias assessment
Prognostic factor measurement	Unclear	See ‘Notes’ above for link to risk of bias assessment
Outcome measurement	Unclear	See ‘Notes’ above for link to risk of bias assessment
Adjustment for other prognostic factors	No	See ‘Notes’ above for link to risk of bias assessment
Statistical analysis and reporting	No	See ‘Notes’ above for link to risk of bias assessment

**Table:** Nelson 1989

***Study characteristics***		
Methods	Study design: registry data Location: USA Time period: recruitment: 13 October 1983 to 30 November 1987 Outcome: PDR Outcome measurement: incidence, RR, and incidence rate ratio	
Participants	Number of participants: 953 Diabetes type: T2D Inclusion criteria: all diabetic people who lived in the Gila River Indian Community at any time between 13 October 1983 and 30 November 1987; whose heritage was at least 50% Pima, Papago, or a mixture of these closely related tribes; and who had undergone biennial research examinations Exclusion criteria: not reported	
Notes	Link to data extraction table, risk of bias assessment, and results: osf.io/dc2bf/?view_only=e50fc6f6134c4f26b635768d365a4ef1	

**Item**	**Authors' judgement**	**Support for judgement**
Study participation	Unclear	See ‘Notes’ above for link to risk of bias assessment
Study attrition	No	See ‘Notes’ above for link to risk of bias assessment
Prognostic factor measurement	Unclear	See ‘Notes’ above for link to risk of bias assessment
Outcome measurement	Unclear	See ‘Notes’ above for link to risk of bias assessment
Adjustment for other prognostic factors	Unclear	See ‘Notes’ above for link to risk of bias assessment
Statistical analysis and reporting	Unclear	See ‘Notes’ above for link to risk of bias assessment

**Table:** Nielsen 1984

***Study characteristics***		
Methods	Study design: prospective cohort longitudinal Location: Denmark Time period: recruitment: 1980 to 1982 Outcome: PDR Outcome measurement: incidence	
Participants	Number of participants: 227 Diabetes type: T1D Inclusion criteria: insulin‐treated diabetes, identified from prescription registrations for insulin; 1 year follow‐up Exclusion criteria: not reported	
Notes	Link to data extraction table, risk of bias assessment, and results: osf.io/dw5hu/?view_only=95205550ab53454c903a2e3b4178aa49	

**Item**	**Authors' judgement**	**Support for judgement**
Study participation	Unclear	See ‘Notes’ above for link to risk of bias assessment
Study attrition	Unclear	See ‘Notes’ above for link to risk of bias assessment
Prognostic factor measurement	Unclear	See ‘Notes’ above for link to risk of bias assessment
Outcome measurement	Unclear	See ‘Notes’ above for link to risk of bias assessment
Adjustment for other prognostic factors	No	See ‘Notes’ above for link to risk of bias assessment
Statistical analysis and reporting	Unclear	See ‘Notes’ above for link to risk of bias assessment

**Table:** Nordwall 2015

***Study characteristics***		
Methods	Study design: retrospective cohort longitudinal Location: Sweden Time period: diagnosed 1983 to 1987 and followed until 2005 to 2008 Outcome: PDR Outcome measurement: incidence and mean difference	
Participants	Number of participants: 451 Diabetes type: T1D Inclusion criteria: T1D diagnosed 1983 to 1987 in Southeast Sweden, < 35 years of age and on insulin < 6 months from diagnosis Exclusion criteria: not reported	
Notes	Link to data extraction table, risk of bias assessment, and results: osf.io/d4k5n/?view_only=414f9ee474174fc8a81333ffb9967a5f	

**Item**	**Authors' judgement**	**Support for judgement**
Study participation	Unclear	See ‘Notes’ above for link to risk of bias assessment
Study attrition	Unclear	See ‘Notes’ above for link to risk of bias assessment
Prognostic factor measurement	Unclear	See ‘Notes’ above for link to risk of bias assessment
Outcome measurement	Unclear	See ‘Notes’ above for link to risk of bias assessment
Adjustment for other prognostic factors	Unclear	See ‘Notes’ above for link to risk of bias assessment
Statistical analysis and reporting	Unclear	See ‘Notes’ above for link to risk of bias assessment

**Table:** Okudaira 2000

***Study characteristics***		
Methods	Study design: prospective cohort longitudinal Location: Japan Time period: first visited clinic 1980 to 1989 Outcome: PDR Outcome measurement: incidence, and HR	
Participants	Number of participants: 527 Diabetes type: T2D Inclusion criteria: first visited the outpatient clinic between 1980 and 1989; exhibited neither proteinuria nor PDR at the first visit; were seen at the clinic for at least 1 year; underwent fundus examination through dilated pupils by ophthalmologists at least once a year during the follow‐up Exclusion criteria: only one fundus examination during follow‐up	
Notes	Link to data extraction table, risk of bias assessment, and results: osf.io/hdz59/?view_only=e8e573cc08e441d088dc630e8adf5093	

**Item**	**Authors' judgement**	**Support for judgement**
Study participation	Unclear	See ‘Notes’ above for link to risk of bias assessment
Study attrition	Unclear	See ‘Notes’ above for link to risk of bias assessment
Prognostic factor measurement	Unclear	See ‘Notes’ above for link to risk of bias assessment
Outcome measurement	Unclear	See ‘Notes’ above for link to risk of bias assessment
Adjustment for other prognostic factors	Unclear	See ‘Notes’ above for link to risk of bias assessment
Statistical analysis and reporting	Yes	See ‘Notes’ above for link to risk of bias assessment

**Table:** Pambianco 2006

***Study characteristics***		
Methods	Study design: prospective cohort longitudinal Location: USA Time period: baselines 1986 to 1988; follow‐up to December 2000 Outcome: PDR Outcome measurement: cumulative incidence and incidence density per 100 person‐years	
Participants	Number of participants: 1124 Diabetes type: T1D Inclusion criteria: T1D diabetes; diagnosed or seen within 1 year of diagnosis at Children’s Hospital of Pittsburgh between 1 January 1950 to 31 May 1980 and living within 100 miles or 2.5 hours from Pittsburgh Exclusion criteria: no death certificate; no follow‐up for the relevant durations	
Notes	Link to data extraction table, risk of bias assessment, and results: osf.io/mh9f3/?view_only=145b0dc33f5b4679ae2cad5ccabed10b	

**Item**	**Authors' judgement**	**Support for judgement**
Study participation	Unclear	See ‘Notes’ above for link to risk of bias assessment
Study attrition	Unclear	See ‘Notes’ above for link to risk of bias assessment
Prognostic factor measurement	Unclear	See ‘Notes’ above for link to risk of bias assessment
Outcome measurement	Unclear	See ‘Notes’ above for link to risk of bias assessment
Adjustment for other prognostic factors	No	See ‘Notes’ above for link to risk of bias assessment
Statistical analysis and reporting	Unclear	See ‘Notes’ above for link to risk of bias assessment

**Table:** Pirart 1977

***Study characteristics***		
Methods	Study design: prospective cohort longitudinal Location: Belgium Time period: 1947 to 1973 Outcome: PDR Outcome measurement: incidence, mean, median and percentage difference, OR, and standardised regression estimates	
Participants	Number of participants: 3250 Diabetes type: not reported Inclusion criteria: proven diabetes for 2 to 25 years – documented hyperglycaemia found on several occasions of true blood sugar above 140 mg per decilitre either fasting or at least 2 hours after ingestion of 50 g of glucose, fasting; consulted for a complication of or instability of diabetes; both retinas visible Exclusion criteria: diabetes duration of more than 25 years	
Notes	Link to data extraction table, risk of bias assessment, and results: osf.io/zuqfa/?view_only=7d8d92459e3f45f8ac13d00717b7b263	

**Item**	**Authors' judgement**	**Support for judgement**
Study participation	Unclear	See ‘Notes’ above for link to risk of bias assessment
Study attrition	No	See ‘Notes’ above for link to risk of bias assessment
Prognostic factor measurement	Unclear	See ‘Notes’ above for link to risk of bias assessment
Outcome measurement	Unclear	See ‘Notes’ above for link to risk of bias assessment
Adjustment for other prognostic factors	No	See ‘Notes’ above for link to risk of bias assessment
Statistical analysis and reporting	No	See ‘Notes’ above for link to risk of bias assessment

**Table:** Porta 2001

***Study characteristics***		
Methods	Study design: prospective cohort longitudinal Location: Europe Time period: baseline data 1989 to 1991 Outcome: PDR Outcome measurement: incidence, mean, median and percentage difference, OR, and standardised regression estimates	
Participants	Number of participants: 3250 Diabetes type: T1D Inclusion criteria: diagnosed T1D < 36 years; insulin within 1 year of onset; age 15 to 60 years Exclusion criteria: centre drop‐out; no retinal photo at baseline or at follow‐up; PDR at baseline	
Notes	Link to data extraction table, risk of bias assessment, and results: osf.io/hrp89/?view_only=96d5233896414d3a928a28b4e71441d7	

**Item**	**Authors' judgement**	**Support for judgement**
Study participation	Unclear	See ‘Notes’ above for link to risk of bias assessment
Study attrition	Unclear	See ‘Notes’ above for link to risk of bias assessment
Prognostic factor measurement	Yes	See ‘Notes’ above for link to risk of bias assessment
Outcome measurement	Yes	See ‘Notes’ above for link to risk of bias assessment
Adjustment for other prognostic factors	Yes	See ‘Notes’ above for link to risk of bias assessment
Statistical analysis and reporting	Yes	See ‘Notes’ above for link to risk of bias assessment

**Table:** Rodriguez‐Villalobos 2005

***Study characteristics***		
Methods	Study design: prospective cohort longitudinal Location: Mexico Time period: April 1992 to July 2004 Outcome: PDR Outcome measurement: incidence	
Participants	Number of participants: 100 Diabetes type: T2D Inclusion criteria: adults with T2D detected in random home visits in suburbs with different socioeconomic areas, who voluntarily accepted participation in the study Exclusion criteria: participants (from initial cohort) who could not be evaluated to determine whether they developed DR or progressed, due to change of address, refusal to participate, or death	
Notes	Link to data extraction table, risk of bias assessment, and results: osf.io/hxe2a/?view_only=6efd4af757c84fe397e8bba5246032b0	

**Item**	**Authors' judgement**	**Support for judgement**
Study participation	No	See ‘Notes’ above for link to risk of bias assessment
Study attrition	No	See ‘Notes’ above for link to risk of bias assessment
Prognostic factor measurement	No	See ‘Notes’ above for link to risk of bias assessment
Outcome measurement	Unclear	See ‘Notes’ above for link to risk of bias assessment
Adjustment for other prognostic factors	No	See ‘Notes’ above for link to risk of bias assessment
Statistical analysis and reporting	Unclear	See ‘Notes’ above for link to risk of bias assessment

**Table:** Roy 2006

***Study characteristics***		
Methods	Study design: prospective cohort longitudinal Location: USA Time period: original cohort 1993 to 1998; follow‐up after 6 years Outcome: PDR and HRC‐PDR Outcome measurement: incidence and OR	
Participants	Number of participants: 725 Diabetes type: T1D Inclusion criteria: African Americans with T1D; treated with insulin before 30 years of age, and who were receiving insulin at time of study; participated in the New Jersey 725 study 1993 to 1998 Exclusion criteria: T2D; diagnosed after 30 years; maturity‐onset diabetes of youth	
Notes	Link to data extraction table, risk of bias assessment, and results: osf.io/z84e5/?view_only=0192b34ce2654bd2ac33e8c8b02e5754	

**Item**	**Authors' judgement**	**Support for judgement**
Study participation	Unclear	See ‘Notes’ above for link to risk of bias assessment
Study attrition	Unclear	See ‘Notes’ above for link to risk of bias assessment
Prognostic factor measurement	Unclear	See ‘Notes’ above for link to risk of bias assessment
Outcome measurement	Yes	See ‘Notes’ above for link to risk of bias assessment
Adjustment for other prognostic factors	Unclear	See ‘Notes’ above for link to risk of bias assessment
Statistical analysis and reporting	Yes	See ‘Notes’ above for link to risk of bias assessment

**Table:** Rudnisky 2017

***Study characteristics***		
Methods	Study design: registry data Location: Canada Time period: November 1999 to November 2009 Outcome: PDR Outcome measurement: incidence	
Participants	Number of participants: 2842 Diabetes type: not reported Inclusion criteria: DM (not defined, includes self‐diagnosis, lab‐confirmed, taking diabetic medications); underwent at least two tele‐ophthalmology examinations between November 1999 and November 2009 and first visit occurred before 31 December 2007 Exclusion criteria: absent or ungradable retinal photographs	
Notes	Link to data extraction table, risk of bias assessment, and results: osf.io/jdzc8/?view_only=3ba4db20f11d4ea9aa0696d61170b15e	

**Item**	**Authors' judgement**	**Support for judgement**
Study participation	Unclear	See ‘Notes’ above for link to risk of bias assessment
Study attrition	Unclear	See ‘Notes’ above for link to risk of bias assessment
Prognostic factor measurement	Unclear	See ‘Notes’ above for link to risk of bias assessment
Outcome measurement	Unclear	See ‘Notes’ above for link to risk of bias assessment
Adjustment for other prognostic factors	No	See ‘Notes’ above for link to risk of bias assessment
Statistical analysis and reporting	Unclear	See ‘Notes’ above for link to risk of bias assessment

**Table:** Silva 2015

***Study characteristics***		
Methods	Study design: prospective cohort longitudinal Location: USA Time period: not reported Outcome: PDR and HRC‐PDR Outcome measurement: incidence	
Participants	Number of participants: 100 Diabetes type: T1D and T2D Inclusion criteria: age 18 years or older, diagnosis of T1D or T2D as defined by the American Diabetes Association; willingness to comply with the study imaging procedures, and willingness to sign the institutionally approved informed consent form for the study Exclusion criteria: no history of diabetes; history of a condition in either eye that might preclude pupil dilation, or using eye drops (mydriatic or miotic) that would alter pupil size or reactivity	
Notes	Link to data extraction table, risk of bias assessment, and results: osf.io/ngjbx/?view_only=bfc4ccc7f0f547c3813e51fdb59c209c	

**Item**	**Authors' judgement**	**Support for judgement**
Study participation	Unclear	See ‘Notes’ above for link to risk of bias assessment
Study attrition	No	See ‘Notes’ above for link to risk of bias assessment
Prognostic factor measurement	Unclear	See ‘Notes’ above for link to risk of bias assessment
Outcome measurement	Yes	See ‘Notes’ above for link to risk of bias assessment
Adjustment for other prognostic factors	Unclear	See ‘Notes’ above for link to risk of bias assessment
Statistical analysis and reporting	Yes	See ‘Notes’ above for link to risk of bias assessment

**Table:** Simonsen 1980

***Study characteristics***		
Methods	Study design: prospective cohort longitudinal Location: Denmark Time period: initial examination: 1964 to 1966; final re‐examination: 1972 to 1979 Outcome: PDR Outcome measurement: incidence	
Participants	Number of participants: 141 Diabetes type: T1D Inclusion criteria: T1D; age 17 to 50 years; controlled at regular intervals at the Steno Memorial Hospital; DM diagnosed prior to 40 years Exclusion criteria: refractive errors exceeding 3D; eye diseases other than DR; consumption of drugs known to influence electroretinogram; pregnant	
Notes	Link to data extraction table, risk of bias assessment, and results: osf.io/ryvfc/?view_only=25209546db9e497a9008cd7e44de5ac6	

**Item**	**Authors' judgement**	**Support for judgement**
Study participation	Unclear	See ‘Notes’ above for link to risk of bias assessment
Study attrition	No	See ‘Notes’ above for link to risk of bias assessment
Prognostic factor measurement	Unclear	See ‘Notes’ above for link to risk of bias assessment
Outcome measurement	Unclear	See ‘Notes’ above for link to risk of bias assessment
Adjustment for other prognostic factors	No	See ‘Notes’ above for link to risk of bias assessment
Statistical analysis and reporting	No	See ‘Notes’ above for link to risk of bias assessment

**Table:** Skrivarhaug 2006

***Study characteristics***		
Methods	Study design: prospective cohort longitudinal Location: Norway Time period: examined for DR at baseline between 1989 and 1990 and at follow‐up from 2002 to 2003 Outcome: PDR Outcome measurement: incidence and RR (95% CI)	
Participants	Number of participants: 368 Diabetes type: T1D Inclusion criteria: all new‐onset cases of T1D in Norway in children below 15 years of age, between 1973 and 1982, examined for DR at baseline between 1989 and 1990 and at follow‐up from 2002 to 2003 Exclusion criteria: not reported	
Notes	Link to data extraction table, risk of bias assessment, and results: osf.io/hyqwt/?view_only=fc20c8d029cc4953838052dd1b77a1fa	

**Item**	**Authors' judgement**	**Support for judgement**
Study participation	Unclear	See ‘Notes’ above for link to risk of bias assessment
Study attrition	Unclear	See ‘Notes’ above for link to risk of bias assessment
Prognostic factor measurement	Unclear	See ‘Notes’ above for link to risk of bias assessment
Outcome measurement	Unclear	See ‘Notes’ above for link to risk of bias assessment
Adjustment for other prognostic factors	Unclear	See ‘Notes’ above for link to risk of bias assessment
Statistical analysis and reporting	Unclear	See ‘Notes’ above for link to risk of bias assessment

**Table:** Styles 2000

***Study characteristics***		
Methods	Study design: retrospective case‐control longitudinal Location: UK Time period: initial examination: not reported Outcome: PDR Outcome measurement: mean, median, and percentage difference	
Participants	Number of participants: 52 Diabetes type: T1D Inclusion criteria: individuals with T1D for 40 years or more; T1D defined as occurring in those in whom the diagnosis was made before age 30 years and insulin therapy commenced at onset or within 1 year of diagnosis Exclusion criteria: not reported	
Notes	Link to data extraction table, risk of bias assessment, and results: osf.io/fqam5/?view_only=8a524b3baa3b4a5abb238d66a7782463	

**Item**	**Authors' judgement**	**Support for judgement**
Study participation	Unclear	See ‘Notes’ above for link to risk of bias assessment
Study attrition	No	See ‘Notes’ above for link to risk of bias assessment
Prognostic factor measurement	Unclear	See ‘Notes’ above for link to risk of bias assessment
Outcome measurement	Unclear	See ‘Notes’ above for link to risk of bias assessment
Adjustment for other prognostic factors	Unclear	See ‘Notes’ above for link to risk of bias assessment
Statistical analysis and reporting	Unclear	See ‘Notes’ above for link to risk of bias assessment

**Table:** Teuscher 1988

***Study characteristics***		
Methods	Study design: prospective cohort longitudinal Location: Switzerland Time period: baseline 1974 to 1975; follow‐up 1982 to 1983 Outcome: PDR Outcome measurement: incidence	
Participants	Number of participants: 534 Diabetes type: T1D and T2D Inclusion criteria: age range 35 to 54 years; diagnosed at least 1 year earlier and under treatment for the disorder by the physicians of the participating centre at a defined date Exclusion criteria: specialist clinics known to attract patients referred, for example, because of particular complications of diabetes	
Notes	Eligibility criteria extracted from 'Prevalence of small vessel and large vessel disease in diabetic patients from 14 centres' (Teuscher 1988, secondary reference). Link to data extraction table, risk of bias assessment, and results: osf.io/bvgws/?view_only=cd78263931c044808acad3e64538ce3c	

**Item**	**Authors' judgement**	**Support for judgement**
Study participation	Unclear	See ‘Notes’ above for link to risk of bias assessment
Study attrition	Unclear	See ‘Notes’ above for link to risk of bias assessment
Prognostic factor measurement	Unclear	See ‘Notes’ above for link to risk of bias assessment
Outcome measurement	Yes	See ‘Notes’ above for link to risk of bias assessment
Adjustment for other prognostic factors	No	See ‘Notes’ above for link to risk of bias assessment
Statistical analysis and reporting	Unclear	See ‘Notes’ above for link to risk of bias assessment

**Table:** Valone 1981

***Study characteristics***		
Methods	Study design: retrospective cohort longitudinal Location: USA Time period: 1980 Outcome: PDR Outcome measurement: incidence	
Participants	Number of participants: 203 Diabetes type: not reported Inclusion criteria: PDR in one eye, NPDR in fellow eye Exclusion criteria: no prior treatment of DR; no other retinal disease at baseline	
Notes	Link to data extraction table, risk of bias assessment, and results: osf.io/pbuwn/?view_only=33735ad697a14fa787b4f047918ffa56	

**Item**	**Authors' judgement**	**Support for judgement**
Study participation	Unclear	See ‘Notes’ above for link to risk of bias assessment
Study attrition	Unclear	See ‘Notes’ above for link to risk of bias assessment
Prognostic factor measurement	Unclear	See ‘Notes’ above for link to risk of bias assessment
Outcome measurement	Unclear	See ‘Notes’ above for link to risk of bias assessment
Adjustment for other prognostic factors	No	See ‘Notes’ above for link to risk of bias assessment
Statistical analysis and reporting	Unclear	See ‘Notes’ above for link to risk of bias assessment

**Table:** Varma 2010

***Study characteristics***		
Methods	Study design: prospective cohort longitudinal Location: USA Time period: baseline clinical examination 2000 to 2003; 4‐year follow‐up examination 2004 to 2008 Outcome: PDR and HRC‐PDR Outcome measurement: incidence	
Participants	Number of participants: 904 Diabetes type: T1D and T2D Inclusion criteria: self‐identification as Latino or of Latino heritage; age 40 years or older on the day of the household screening for the Los Angeles Latino Eye Study; residency in one of the selected La Puente census tracts; definite DM diagnosis Exclusion criteria: not reported	
Notes	Link to data extraction table, risk of bias assessment, and results: osf.io/e5h24/?view_only=9d4bde926ce64592a109b012f79d3ffb	

**Item**	**Authors' judgement**	**Support for judgement**
Study participation	Unclear	See ‘Notes’ above for link to risk of bias assessment
Study attrition	Unclear	See ‘Notes’ above for link to risk of bias assessment
Prognostic factor measurement	Unclear	See ‘Notes’ above for link to risk of bias assessment
Outcome measurement	Yes	See ‘Notes’ above for link to risk of bias assessment
Adjustment for other prognostic factors	No	See ‘Notes’ above for link to risk of bias assessment
Statistical analysis and reporting	Unclear	See ‘Notes’ above for link to risk of bias assessment

**Table:** Verdaguer 2009

***Study characteristics***		
Methods	Study design: retrospective cohort longitudinal Location: Chile Time period: 1971 to 2008 Outcome: PDR Outcome measurement: incidence, means and percentages of progressors versus non‐progressors to PDR, OR	
Participants	Number of participants: 39 Diabetes type: T1D Inclusion criteria: T1D Exclusion criteria: not reported	
Notes	Link to data extraction table, risk of bias assessment, and results: osf.io/vxnk2/?view_only=e3605c9776844cd2b6449037457ad5fd	

**Item**	**Authors' judgement**	**Support for judgement**
Study participation	No	See ‘Notes’ above for link to risk of bias assessment
Study attrition	No	See ‘Notes’ above for link to risk of bias assessment
Prognostic factor measurement	No	See ‘Notes’ above for link to risk of bias assessment
Outcome measurement	Unclear	See ‘Notes’ above for link to risk of bias assessment
Adjustment for other prognostic factors	No	See ‘Notes’ above for link to risk of bias assessment
Statistical analysis and reporting	No	See ‘Notes’ above for link to risk of bias assessment

**Table:** Vesteinsdottir 2010

***Study characteristics***		
Methods	Study design: retrospective cohort longitudinal Location: Iceland Time period: not reported Outcome: PDR Outcome measurement: incidence	
Participants	Number of participants: 76 Diabetes type: T1D and T2D Inclusion criteria: diabetes; having regular retinopathy screening; PDR in one or both eyes Exclusion criteria: not reported	
Notes	Link to data extraction table, risk of bias assessment, and results: osf.io/crwxa/?view_only=2a40812ea9bd4739baa4dae8f8f597bb	

**Item**	**Authors' judgement**	**Support for judgement**
Study participation	Unclear	See ‘Notes’ above for link to risk of bias assessment
Study attrition	No	See ‘Notes’ above for link to risk of bias assessment
Prognostic factor measurement	Unclear	See ‘Notes’ above for link to risk of bias assessment
Outcome measurement	Unclear	See ‘Notes’ above for link to risk of bias assessment
Adjustment for other prognostic factors	No	See ‘Notes’ above for link to risk of bias assessment
Statistical analysis and reporting	Unclear	See ‘Notes’ above for link to risk of bias assessment

**Table:** Voigt 2018

***Study characteristics***		
Methods	Study design: retrospective cohort longitudinal Location: Germany Time period: 1987 to 2014 Outcome: PDR Outcome measurement: incidence	
Participants	Number of participants: 2272 Diabetes type: T2D Inclusion criteria: fundoscopy reports of three consecutive years available Exclusion criteria: clinically significant macular oedema	
Notes	Link to data extraction table, risk of bias assessment, and results: osf.io/w3dvg/?view_only=70d6e41449714f8ab22dd70306e3b936	

**Item**	**Authors' judgement**	**Support for judgement**
Study participation	Unclear	See ‘Notes’ above for link to risk of bias assessment
Study attrition	No	See ‘Notes’ above for link to risk of bias assessment
Prognostic factor measurement	Unclear	See ‘Notes’ above for link to risk of bias assessment
Outcome measurement	Unclear	See ‘Notes’ above for link to risk of bias assessment
Adjustment for other prognostic factors	Unclear	See ‘Notes’ above for link to risk of bias assessment
Statistical analysis and reporting	Unclear	See ‘Notes’ above for link to risk of bias assessment

**Table:** WESDR

***Study characteristics***		
Methods	Study design: prospective cohort longitudinal Location: USA Time period: baseline: 1980 to 1982; follow‐up 1984 to 1986 Outcome: PDR Outcome measurement: incidence, OR, and RR	
Participants	Number of participants: 2366 Diabetes type: T1DM and T2DM Inclusion criteria: younger‐onset group: IDD before 30 years. Older‐onset group: diagnosed with DM at 30 years or older and diagnosis confirmed by a casual or a postprandial serum glucose level of at least 11.1 mmol/L or a fasting serum glucose level of 7.8 mmol/L or greater on at least two occasions. Exclusion criteria: not reported	
Notes	Link to data extraction table, risk of bias assessment, and results: osf.io/uh5sx/?view_only=8ed60b81079b4f8dab6a352967b07b4a	

**Item**	**Authors' judgement**	**Support for judgement**
Study participation	Yes	See ‘Notes’ above for link to risk of bias assessment
Study attrition	Unclear	See ‘Notes’ above for link to risk of bias assessment
Prognostic factor measurement	Unclear	See ‘Notes’ above for link to risk of bias assessment
Outcome measurement	Yes	See ‘Notes’ above for link to risk of bias assessment
Adjustment for other prognostic factors	Unclear	See ‘Notes’ above for link to risk of bias assessment
Statistical analysis and reporting	Yes	See ‘Notes’ above for link to risk of bias assessment

**Table:** Yokoyama 1994

***Study characteristics***		
Methods	Study design: retrospective cohort longitudinal Location: Japan Time period: 1951 to 1984 Outcome: PDR Outcome measurement: incidence	
Participants	Number of participants: 373 Diabetes type: T1D Inclusion criteria: all patients who visited the Diabetes Centre, Tokyo Women’s Medical College, in whom IDDM had been diagnosed between 1951 and 1984 and before the age of 30 years Exclusion criteria: incomplete registration; PDR	
Notes	Link to data extraction table, risk of bias assessment, and results: osf.io/j5ufs/?view_only=747af7be9845495aa647787470791f85	

**Item**	**Authors' judgement**	**Support for judgement**
Study participation	Unclear	See ‘Notes’ above for link to risk of bias assessment
Study attrition	No	See ‘Notes’ above for link to risk of bias assessment
Prognostic factor measurement	Unclear	See ‘Notes’ above for link to risk of bias assessment
Outcome measurement	Unclear	See ‘Notes’ above for link to risk of bias assessment
Adjustment for other prognostic factors	No	See ‘Notes’ above for link to risk of bias assessment
Statistical analysis and reporting	Unclear	See ‘Notes’ above for link to risk of bias assessment

**Table:** Zavrelova 2011

***Study characteristics***		
Methods	Study design: retrospective cohort longitudinal Location: the Netherlands Time period: 1998 to 2005 Outcome: PDR Outcome measurement: mean, median, and percentage difference	
Participants	Number of participants: 3343 Diabetes type: T2D Inclusion criteria: T2D Exclusion criteria: T1D; no visit or one visit only for graded fundus photographs	
Notes	Link to data extraction table, risk of bias assessment, and results: osf.io/srwv8/?view_only=e42486a93e6249f080791756a0a515e0	

**Item**	**Authors' judgement**	**Support for judgement**
Study participation	Unclear	See ‘Notes’ above for link to risk of bias assessment
Study attrition	No	See ‘Notes’ above for link to risk of bias assessment
Prognostic factor measurement	Unclear	See ‘Notes’ above for link to risk of bias assessment
Outcome measurement	Unclear	See ‘Notes’ above for link to risk of bias assessment
Adjustment for other prognostic factors	No	See ‘Notes’ above for link to risk of bias assessment
Statistical analysis and reporting	Unclear	See ‘Notes’ above for link to risk of bias assessment

**DM:** diabetes mellitus; **DBP:** diastolic blood pressure; **DR:** diabetic retinopathy; **HbA1c:** glycated haemoglobin; **HR:** hazard ratio; **IDDM:** insulin‐dependent diabetes mellitus; **NIDDM:** non‐insulin‐dependent diabetes mellitus; **NPDR:** non‐proliferative diabetic retinopathy; **OR:** odds ratio; **PDR:** proliferative diabetic retinopathy; **RR:** risk ratio; **T1D:** type 1 diabetes; **T2D:** type 2 diabetes; **WHO:** World Health Organization

### Characteristics of excluded studies [ordered by study ID]

**Table:**

Study	Reason for exclusion
Abougalambou 2015	Cross‐sectional study
Adderley 2020	No data on development of PDR
Adnitt 1970	No data on development of PDR
Advance Collaborative Group 2008	No data on development of PDR
Alattas 2022	Ineligible study design
Altaf 2013	Cross‐sectional study
Ashakiran 2011	Unable to access publication ‐ no contact details for author
Askarishahi 2011	Cross‐sectional study
Barr 2001	RCT
Beulens 2009	RCT
Borch‐Johnsen 1987	No data on development of PDR
Bresnick 1987	Ineligible patient population
Brinchmann‐Hansen 1985	RCT
Burton 2000	Case report
Cantagallo 1989	Review article
Chahal 1985	No data on development of PDR
Chaturvedi 1998	RCT
Chawla 2015	Cross‐sectional study
Chen 2017	No data on development of PDR
Chen 2020	Abstract ‐ no corresponding publication found
Chew 2014	RCT
Chittimoju 2013	Cross‐sectional study
Chung 2019	No data on development of PDR
Cikamatana 2007	No data on development of PDR
Cohen 1991	Rsk factors not given in a way that can be used to determine risk of PDR
Colin 2012	Abstract ‐ no corresponding publication found
Cuadras 2017	Cross‐sectional study, no data on development of PDR, and an abstract only
Danielsen 1983	Cross‐sectional study
DCCT 1993	RCT
Ding 2020	Ineligible study design ‐ DR self‐reported
Dizdarevic 2012	Cross‐sectional study
Duckworth 2009	RCT
Dujic 1998	Cross‐sectional study
Elshafei 2011	Cross‐sectional study
Estacio 2000	RCT
ETDRS 1991c	Ineligible study design (RCT)
Foshati 2019	Cross‐sectional study
Friberg 1985	No data on development of PDR
Fung 2011	Cross‐sectional study
Fusi‐Rubiano 2015	Cross‐sectional study
Gallagher 2018	No data on development of PDR
Gao 2014	Cross‐sectional study
Garcia‐Medina 2011	No data on development of PDR
Garmo 2018	No data on development of PDR
Geetha 2019	Ineligible study design
González‐Villalpando 1994	Cross‐sectional study
Grauslund 2011	Review article
Gupta 2019	Survey, no data on development of PDR, and abstract only
Haffner 1993	No data on development of PDR
Hamman 1989	Cross‐sectional study
Hautala 2018	No data on development of PDR
Henricsson 1997	No data on development of PDR
Hoang 2020	Cross‐sectional study
Holman 2008	No data on development of PDR
Holt 2019	No data on development of PDR
Hwang 2019	No data on development of PDR
Jacobsen 2003	Unable to access publication ‐ author contacted but no response
Janka 1989	No data on development of PDR
Jin 2018	Cross‐sectional study
Jonas 2017	No data on development of PDR
Kawasaki 2005	Review article
Keech 2007	RCT
Kingsley 1988	No data on development of PDR
Klein 1988	No data on development of PDR
Kohner 1986	RCT
Kornerup 1955	No data on development of PDR
Kroc Collaborative Study 1984	RCT
Krolewski 1986	Unspecified number of patient population less than 18 years of age
Kulenovic 2006	No data on risk factors
Kyari 2014	Cross‐sectional study
Lamoureux 2018	No data on development of PDR
LaPorte 1986	No data on development of PDR
Lauritzen 1983	RCT
Lee 2010	No data on development of PDR
Lee 2011	Cross‐sectional study
Leese 2004	Correspondence author contacted to provide clarification on definition of outcome. Outcome may have included participants with pre‐PDR, so not included in review
Leske 2003	No data on risk factors
Li 2019	Ineligible outcomes
Lim 2015	Abstract
Lima 2016	Ineligible study design
Lin 2010	Ineligible outcomes
Liu 2019	Cross‐sectional study, and abstract only
Lorenzi 1983	Ineligible patient population ‐ adolescents
Low 2021	No data on development of PDR
Lu 2012	Cross‐sectional study
Lu 2013	Cross‐sectional study
Lv 2020	Cross‐sectional study
Malik 2005	Cross‐sectional study
Malik 2018	Cross‐sectional study
Man 2018	No data on development of PDR
Marshall 1993	No data on development of PDR
Mathur 2016	Correspondence author contacted to provide clarification on definition of outcome. Supplementary material provided which confirmed outcome of 'severe retinopathy' included participants with diabetic macular oedema
Mehlsen 2011	No data on development of PDR
Mehta 2019	RCT
Minuto 2009	Cross‐sectional study
Mitchell 1985	No data on development of PDR
Mohd Ali 2016	Cross‐sectional study
Moon 2018	Abstract
Mowatt 2013	Cross‐sectional study
Muhlhauser 1986	Cross‐sectional study
Muqit 2014	No data on risk factors
Nguyen 2009	Cross‐sectional study
Nicolucci 1993	Cross‐sectional study
Nordwall 2004	No data on development of PDR
Ohtani 1985	Ineligible study design
Padmajeya 2019	Abstract
Paetkau 1977	Cross‐sectional study
Pardhan 2004	Cross‐sectional study
Park 2015	Cross‐sectional study
Parving 1988	Cross‐sectional study
Pettitt 1980	No data on development of PDR
Pugliese 2012	Cross‐sectional study
Rahul 2015	Cross‐sectional study
Rasoulinejad 2022	Cross‐sectional study
Raum 2015	Cross‐sectional study
Ravi Theja 2017	Cross‐sectional study, and abstract
Reichard 1991	RCT
Resman 1993	Ineligible study design
Romero 2007	No DR at baseline
Romero‐Aroca 2016	No data on development of PDR
Rudnisky 2012	No data on development of PDR
Schellhase 2003	No data on development of PDR
Shriwas 1996	Cross‐sectional study
Singh 2020	No data on development of PDR
Sjolie 1985	No data on risk factors
Srikanth 2015	Ineligible participant population
Takaike 2018	Definition of outcome unclear and unable to contact authors to clarify
Tapp 2006	No data on risk factors
Taylor 1997	Cross‐sectional study
Thapa 2015	Cross‐sectional study
Tripathi 2010	No data on risk factors
Tseng 2012	No data on development of PDR
Tu 2011	No data on development of PDR
UKPDS Group 1996	No data on risk factors
Van Leiden 2003	No data on development of PDR
Villena 2011	Cross‐sectional study
Wang 2006	No data on risk factors
Xie 2009	Cross‐sectional study
Yokoyama 1997	Cross‐sectional study
Zhou 2006	No data on development of PDR, and abstract only

**DR:** diabetic retinopathy; **PDR:** proliferative diabetic retinopathy; **RCT:** randomised controlled trial

## Differences between protocol and review

On completion of screening of potentially eligible studies, we decided to exclude RCTs. Initially, our preference had been to include only prospective or retrospective cohort and case‐control longitudinal studies, as these are the most appropriate study designs for the evaluation of the development and progression of diseases and associated prognostic factors. We considered including RCTs, where there was a controlled, untreated arm, as a precaution should there have been insufficient prospective or retrospective cohort and case‐control longitudinal studies available to effectively address our review objectives. However, we identified 59 of these types of studies (87 reports) as relevant, and we therefore decided to exclude RCTs from further consideration, to avoid combining different types of study designs. 

Originally, we had intended to include only studies consisting of cohorts of participants with NPDR or PDR at baseline, to evaluate progression to PDR and HRC‐PDR, respectively. However, on evaluation of potentially eligible studies and prior to commencing data extraction, we made the decision to incorporate those in which a proportion had NPDR or PDR at baseline. This is because it became apparent that most studies incorporated assorted populations of participants with and without DR at baseline, even the larger, long‐term, population‐based studies, such as the Wisconsin Epidemiologic Study of Diabetic Retinopathy, which we considered important to include. We had also intended to review the mean time period for the development of PDR, but this was reported very infrequently in studies, and we therefore decided not to evaluate it.   

In a further clarification of the protocol, we decided to exclude studies which reported on the development and progression of PDR during pregnancy. This is because significant prognostic factors relating to deterioration of DR in pregnancy may be influenced by hormonal changes, alterations in systemic vasculature, and retinal auto regulatory mechanisms. As such, pregnant participants are not comparable to the general population of people with diabetes. We also decided to exclude genetic studies as these were generally cross‐sectional in design.  
